# Global burden of lower respiratory infections attributable to cytomegalovirus, 1990–2021: a systematic analysis from the MICROBE database

**DOI:** 10.3389/fmicb.2025.1693635

**Published:** 2025-11-18

**Authors:** Wanwan Zhang, Min Liu, Haoyu Ji, Emmanuel Mensah, Shirong Li, Hanli Wang, Zhiwei Lu, Shuoshuo Wei, Yusheng Cheng, Lei Zha

**Affiliations:** 1 Department of Pulmonary and Critical Care Medicine, The First Affiliated Hospital of Wannan Medical College (Yijishan Hospital of Wannan Medical College), Wuhu, Anhui, China; 2 Department of Pulmonary and Critical Care Medicine, The Second People's Hospital of Wuhu, Wuhu, Anhui, China

**Keywords:** cytomegalovirus, deaths, disability-adjusted life years, global burden of disease, lower respiratory infection

## Abstract

**Objectives:**

This study aimed to investigate the global epidemiological characteristics and disease burden of lower respiratory infections (LRIs) attributable to cytomegalovirus (CMV) from 1990 to 2021.

**Methods:**

We systematically assessed the global burden and temporal trends of CMV-associated LRIs across different ages, sexes, geographic regions, and socioeconomic statuses using data from the MICROBE database spanning 1990–2021. Key metrics included mortality, disability-adjusted life years (DALYs), and their corresponding age-standardized rates (ASRs)

**Results:**

Globally, the number of DALYs due to CMV-attributed LRI decreased from an estimated 734,208 (95% UI: 612,175–856,241) in 1990 to 530,465 (95% UI: 469,046–591,884) in 2021, while the number of deaths increased from 16,141 (95% UI: 14,247–18,034) to 19,235 (95% UI: 17,204–21,266) over the same period. The age-standardized DALY rate (ASDR) declined from 13.89 (95% UI: 11.86–15.93) in 1990 to 6.95 (95% UI: 6.08–7.83) in 2021. Similarly, the age-standardized mortality rate (ASMR) dropped from 0.40 (95% UI: 0.35–0.44) to 0.24 (95% UI: 0.21–0.27). In 2021, the disease burden was highest in regions with low Socio-demographic Index (SDI). From 1990 to 2021, both ASMR and ASDR for CMV-attributable LRI decreased as SDI increased, and projections indicate a continued decline over the next 30 years.

**Conclusions:**

The global burden of CMV-attributable LRI has declined significantly from 1990 to 2021. However, targeted and cost-effective interventions are urgently needed to prevent and reduce the burden of CMV-associated LRI, particularly in low-SDI regions, children, and the elderly.

## 1 Introduction

Cytomegalovirus (CMV), a ubiquitous β-herpesvirus, is increasingly recognized as a neglected contributor to global morbidity and mortality. It exhibits broad host tropism, establishes lifelong latency, and possesses sophisticated immune evasion mechanisms that enable persistent infection across diverse populations. Globally, 70–90% of individuals are infected with at least one herpesvirus, including CMV (Chan et al., 2022). Seroprevalence is near-universal in many low- and middle-income countries (LMICs), compared with 40–60% in high-income regions, with immunocompetent adults demonstrating 40–100% prevalence globally (Melendez and Razonable, 2015; Forte et al., 2020; Pontes and Araujo Júnior, 2024). While most infections in immunocompetent hosts are clinically silent, CMV establishes latency in monocytes, lymphocytes, and other cells, with potential for periodic reactivation throughout life (Ljungman et al., 2011). In immunocompromised populations, including organ transplant recipients, people living with HIV, and intensive care unit (ICU) patients, reactivation can precipitate severe disease, particularly lower respiratory infections (LRIs; Adewuyi et al., 2012; Ramanan and Razonable, 2013).

CMV pneumonia is well described in high-risk cohorts. Among lung transplant recipients, despite prophylaxis, CMV pneumonia occurs in 20–50% of cases and is associated with impaired graft survival and long-term morbidity. Allogeneic hematopoietic stem cell transplant recipients exhibit reactivation in 30–40% of cases, while people with advanced HIV infection demonstrate CMV seropositivity exceeding 90%, with pneumonitis typically developing when CD4^+^ T-cell counts fall below 50 cells/mm3. These data underscore CMV's clinical importance in immunocompromised hosts (Abedi et al., 2018; Styczynski, 2018; Baburao et al., 2020).

Despite extensive study in high-risk groups, the global epidemiological contribution of CMV to LRI burden remains poorly defined. LRIs are the leading infectious cause of mortality worldwide, causing over 2.3 million deaths annually, predominantly affecting children under five and adults over 70 (Calderaro et al., 2022; Bulata-Pop et al., 2024). Established pathogens include Streptococcus pneumoniae, Haemophilus influenzae, respiratory syncytial virus, and influenza virus, yet CMV is rarely accounted for in large-scale burden assessments (Jain, 2023). The Global Burden of Disease (GBD) 2021 study estimated 344 million LRI episodes and 2.18 million deaths in 2021, but CMV was excluded from the 18-pathogen model, revealing a critical knowledge gap in global LRI epidemiology (Bender et al., 2024). Emerging evidence suggests CMV may synergize with other pathogens, exacerbate respiratory morbidity, and contribute substantially to all-cause LRI mortality (Clausen and Zaffiri, 2020).

Recent studies have highlighted CMV reactivation in critically ill patients, including those with COVID-19. In one ICU cohort, 20.4% of patients experienced CMV blood reactivation, correlating with higher unadjusted mortality and secondary bacterial infections (Gatto et al., 2022). Another prospective study reported 14.8% of critically ill COVID-19 patients with moderate-to-severe ARDS had CMV co-infection, which independently increased ICU mortality (OR 4.91; 95% CI 2.76–8.75), prolonged hospital stays, and was associated with higher rates of ICU-acquired infections and probable invasive pulmonary aspergillosis (López-Olivencia et al., 2025). A systematic review and meta-analysis of 22 studies in immunocompetent critically ill patients found CMV reactivation in 20–71% of cases, linked to a 2.55-fold increase in ICU mortality, longer mechanical ventilation, higher nosocomial infection rates, and prolonged ICU stay (Lachance et al., 2017). Collectively, these findings indicate CMV significantly contributes to adverse outcomes in critical illness, extending beyond traditionally defined high-risk groups.

The COVID-19 pandemic has further emphasized CMV's underappreciated role, suggesting it may act as an amplifier of disease severity through direct organ damage, immune down-regulation, and perpetuation of hyper-inflammatory responses in conditions such as ARDS (Gatto et al., 2022; López-Olivencia et al., 2025; Lachance et al., 2017). Yet global health metrics, including the GBD study, do not quantify CMV-attributable LRI burden, underscoring a pressing public health gap and the need for comprehensive global estimates.

Existing literature is limited by three key gaps. First, epidemiological studies are heavily skewed toward transplant and HIV cohorts, which constitute only a fraction of global LRI cases (Stojcevic-Maletic et al., 2020). Second, most studies are concentrated in high-income countries, with limited systematic data from LMICs, where CMV seroprevalence and LRI mortality are highest. Third, no prior global synthesis has quantified CMV's contribution to LRI burden across general and high-risk populations, constraining evidence-based policy, prevention strategies, and antiviral prioritization. Hence a comprehensive reassessment is urgently needed. CMV's near-universal prevalence, coupled with its role as an opportunistic and potentially synergistic respiratory pathogen, likely underpins a substantial yet underappreciated contribution to global LRI mortality. Systematic quantification of CMV-attributable LRI burden will clarify its relative importance among respiratory pathogens, guide targeted interventions, including antiviral prophylaxis and therapy, and inform vaccine development strategies.

In this study, we present the first systematic global analysis of LRIs attributable to CMV from 1990 to 2021 using the MICROBE database. We examine temporal trends, age- and sex-specific patterns, and geographical heterogeneity to provide a robust assessment of CMV's global impact, compared with other established LRI pathogens. This work addresses a critical knowledge gap, offering new insights into CMV epidemiology and laying the foundation for improved prevention and control strategies aimed at reducing CMV-associated mortality.

## 2 Methods

### 2.1 Data sources

The MICROBE database, developed by the Institute for Health Metrics and Evaluation at the University of Washington, is an open-access resource that provides comprehensive estimates of the burden of cytomegalovirus (CMV)-associated lower respiratory infections (LRIs) across 204 countries and territories. As of 2021, the database contains data on 23 pathogens and 88 pathogen–drug combinations spanning 12 major infectious syndromes (Murray et al., 2022). Disease burden is quantified as both the number and rate of deaths and disability-adjusted life years (DALYs), with 95% uncertainty intervals (UI) based on 1,000 draws from posterior distributions. Details of the statistical methodology can be found in previous publications (Murray et al., 2022). For this study, we extracted estimates of deaths and DALYs related to CMV-associated LRIs from the MICROBE database. The database is freely available for non-commercial use at https://vizhub.healthdata.org/microbe/ following registration and acceptance of the Free-of-Charge Non-commercial User Agreement. The extracted data were aggregated and analyzed at the global level, as well as across the 21 Global Burden of Disease (GBD) regions and 7 GBD super-regions.

### 2.2 Definitions

DALYs and deaths were utilized to assess the burden of LRIs attributable to CMV. DALYs represent the sum of years of life lost (YLL) due to premature mortality and years lived with disability (YLD), thereby capturing both fatal and non-fatal outcomes associated with disease burden. Years of life lost (YLL) refer to the years of potential life lost due to death occurring before the standard life expectancy, while years lived with disability (YLD) are calculated by multiplying the number of affected individuals by a disability weight that quantifies the severity of health loss. Disability weights range from 0 (indicating perfect health) to 1 (equivalent to death; Li et al., 2022; Vos et al., 2020). Socio-demographic index (SDI), reflecting a country's socio-economic status, is computed as the geometric mean of average years of education among individuals aged 15 years and older, total fertility rate for those under age 25, and lag-distributed per capita income. Based on SDI values, countries and territories are classified into five categories: low SDI (0 < SDI < 0.46), low-middle SDI (0.46 ≤ SDI < 0.61), middle SDI (0.61 ≤ SDI < 0.69), high-middle SDI (0.69 ≤ SDI < 0.81), and high SDI (0.81 ≤ SDI ≤ 1.00). Additionally, 204 countries and territories were included in this analysis, which are further grouped geographically into 21 regions.

### 2.3 Statistical analyses

The burden of LRIs attributable to CMV was quantified using age-standardized rates (ASR), estimated annual percentage change (EAPC), and the number and temporal changes in deaths and DALYs. These metrics were analyzed by global, age, sex, geographic, and socioeconomic strata. ASR was calculated using the reference population and is expressed per 1,000 person-years (Wang et al., 2019). The EAPC is a summary of ASR trends over a period of time. If both the EAPC estimate and the lower bound of its 95% confidence interval (CI) are greater than 0, ASR shows an upward trend. Conversely, if both the EAPC estimate and the upper bound of its 95% CI are less than 0, ASR shows a downward trend. Otherwise, ASR is a long term stable trend. In addition, we analyzed the association between LRI burden attributable to CMV and SDI, the association between SDI and age-standardized DALY and death rates was fitted using local estimated scatter plot smoothing regression in 21 GBD regions. We also employed age-period-cohort (APC) modeling to assess the associations between age, period, birth cohort, and CMV-associated LRI mortality (Rosenberg et al., 2014). Bayesian Age-Period-Cohort (BAPC) models were used to project future trends, utilizing integrated nested Laplace approximations (INLA) for full Bayesian inference. Critical features of BAPC models include the generation of age-specific and age-standardized projected rates and the automatic incorporation of Poisson noise for predictive distributions (Chen et al., 2024; Li et al., 2021). All statistical analyses and visualizations were conducted using the R statistical software program (version 4.4.3) and JD_GBDR (V2.31, Jing ding Medical Technology Co., Ltd). *p* < 0.05 was considered statistically significant.

## 3 Results

### 3.1 Trends in the burden of LRI attributable to CMV from 1990 to 2021

In 2021, the global number of DALYs due to CMV-associated LRIs was 530,465 (95% UI: 469,046–591,884), and the number of deaths reached 19,235 (95% UI: 17,204–21,266). Compared to 2019, this represents a moderate decline in both metrics. From 1990 to 2021, the global DALYs attributable to CMV-associated LRIs decreased from 734,208 (95% UI: 612,175–856,241) to 530,465 (95% UI: 469,046–591,884). During the same period, the number of deaths increased from 16,141 (95% UI: 14,247–18,034) to 19,235 (95% UI: 17,204–21,266). Notably, the age-standardized DALY rate declined from 13.89 (95% UI: 11.86–15.93) per 1,000 person-years in 1990 to 6.95 (95% UI: 6.08–7.83) per 1,000 person-years in 2021, while the age-standardized death rate decreased from 0.40 (95% UI: 0.35–0.44) to 0.24 (95% UI: 0.21–0.27) per 1,000 person-years (Tables 1, 2). Regionally, all areas except Southern Latin America and Southern Sub-Saharan Africa showed a negative EAPC for age-standardized DALY and death rates, indicating overall declines in these measures.

**Table 1:** The number and ASR of DALYs for CMV-associated LRI burden in 1990 and 2021, and its temporal trend from 1990 to 2021.

**Location**	**1990**		**2021**		**1990–2021**
	**Number (95% UI)**	**ASR (95% UI)**	**Number (95% UI)**	**ASR (95% UI)**	**EAPC (95% CI)**
Global	734,208	13.89	530,465	6.95	−2.14
	(612,175–856,241)	(11.86–15.93)	(469,046–591,884)	(6.08–7.83)	(−2.23, −2.05)
Sex					
Male	403,033	15.79	297,713	8.11	−2.08
	(333,341–472,726)	(13.52–18.06)	(264,419–331,007)	(7.15–9.08)	(−2.17, −1.99)
Female	331,175	12.32	232,752	5.93	−2.23
	(274,357–387,993)	(10.37–14.27)	(202,316–263,188)	(5.1–6.77)	(−2.32, −2.13)
Central Europe, Eastern	29,072	13.84	20,793	4.43	−2.21
Europe, and Central Asia	(25,590–32,554)	(12.12–15.57)	(18,824–22,761)	(3.91–4.95)	(−2.53, −1.90)
Central Asia	13,681	13.44	6,705	7.49	−2.79
	(11,254–16,108)	(11.66–15.21)	(5,646–7,763)	(6.37–8.61)	(−3.12, −2.46)
Central Europe	6,716	14.86	5,980	3.03	−1.77
	(6,138–7,294)	(12.81–16.92)	(5,411–6,550)	(2.76–3.31)	(−2.05, −1.49)
Eastern Europe	8,648	21.10	8,100	2.73	−1.65
	(7,766–9,531)	(16.9–25.3)	(7,201–8,999)	(2.44–3.02)	(−2.34, −0.96)
High-income	36,009	7.70	38,326	1.81	−1.51
	(32,341–39,677)	(6.69–8.72)	(34,010–42,642)	(1.64–1.98)	(−1.64, −1.37)
Australasia	472	16.26	553	1.06	−1.97
	(421–523)	(13.63–18.89)	(485–622)	(0.94–1.17)	(−2.20, −1.73)
High-income Asia Pacific	7,840	5.21	7,477	1.47	−3.19
	(7,045–8,634)	(4.76–5.66)	(6,362–8,593)	(1.3–1.63)	(−3.40, −2.98)
High-income North America	9,869	3.89	11,983	2.00	−1.07
	(8,818–10,921)	(3.47–4.31)	(10,793–13,172)	(1.82–2.18)	(−1.18, −0.96)
Southern Latin America	2,765	3.14	5,080	5.93	1.01
	(2,551–2,980)	(2.82–3.46)	(4,591–5,568)	(5.38–6.49)	(0.73, 1.29)
Western Europe	15,061	4.29	13,231	1.39	−1.99
	(13,442–16,680)	(3.84–4.75)	(11,586–14,875)	(1.25–1.53)	(−2.19, −1.79)
Latin America and Caribbean	33,586	2.14	39,438	6.61	−0.88
	(29,884–37,287)	(1.91–2.37)	(35,359–43,517)	(5.9–7.31)	(−1.17, −0.60)
Andean Latin America	6,676	2.69	5,033	8.49	−2.02
	(5,586–7,765)	(2.41–2.97)	(4,037–6,030)	(6.82–10.17)	(−2.28, −1.75)
Caribbean	4,134	6.12	3,946	8.17	−0.84
	(3,367–4,901)	(5.63–6.61)	(3,241–4,652)	(6.6–9.73)	(−1.02, −0.66)
Central Latin America	12,540	2.87	14,187	5.82	−1.05
	(11,237–13,842)	(2.57–3.17)	(12,439–15,935)	(5.08–6.55)	(−1.37, −0.72)
Tropical Latin America	10,264	10.64	16,288	6.56	−0.3
	(9,258–11,269)	(9.63–11.65)	(14,889–17,688)	(5.98–7.13)	(−0.62, 0.03)
North Africa and Middle East	47,068	12.17	27,756	5.74	−2.3
	(37,785–56,350)	(10.25–14.09)	(23,654–31,859)	(4.93–6.54)	(−2.49, −2.11)
South Asia	230,299	18.95	141,801	9.76	−2.13
	(180,287–280,311)	(16.42–21.48)	(122,687–160,915)	(8.45–11.07)	(−2.22, −2.04)
Southeast Asia,	186,934	9.44	101,843	4.47	−3.88
East Asia, and Oceania	(161,113–212,754)	(8.65–10.24)	(88,723–114,963)	(3.89–5.04)	(−4.00, −3.75)
East Asia	123,412	9.29	50,991	2.85	−5.52
	(106,149–140,675)	(8.43–10.14)	(40,882–61,099)	(2.33–3.37)	(−5.71, −5.32)
Oceania	1,401	13.34	2,087	16.12	−0.43
	(1,060–1,742)	(11.35–15.34)	(1,575–2,599)	(12.92–19.31)	(−0.55, −0.32)
Southeast Asia	62,165	19.70	48,871	8.12	−1.75
	(51,971–72,359)	(16.27–23.13)	(43,013–54,728)	(7.13–9.11)	(−1.83, −1.67)
Sub-Saharan Africa	171,050	30.10	160,416	19.01	−1.35
	(135,769–206,331)	(25.51–34.69)	(130,036–190,796)	(16.15–21.87)	(−1.46, −1.23)
Central Sub-Saharan Africa	20,295	33.98	17,774	22.12	−1.45
	(14,804–25,786)	(26.81–41.14)	(13,978–21,570)	(17.15–27.09)	(−1.57, −1.34)
Eastern Sub-Saharan Africa	68,527	34.24	52,291	18.92	−1.94
	(54,370–82,685)	(29.12–39.36)	(43,063–61,520)	(16.25–21.59)	(−2.14, −1.75)
Southern Sub-Saharan Africa	7,411	16.72	11,047	16.88	0.75
	(6,404–8,418)	(14.7–18.74)	(9,538–12,555)	(14.68–19.08)	(0.22, 1.28)
Western Sub-Saharan Africa	74,818	29.01	79,299	18.56	−1.24
	(58,696–90,940)	(24.18–33.83)	(60,672–97,925)	(14.99–22.12)	(−1.36, −1.12)

ASR, age-standardized rate; CI, confidence interval; UI, uncertainty interval; EAPC, estimated annual percentage change.

**Table 2:** The number and ASR of deaths for CMV-associated LRI burden in 1990 and 2021, and its temporal trends from 1990 to 2021.

**Location**	**1990**		**2021**		**1990–2021**
	**Number (95% UI)**	**ASR (95% UI)**	**Number (95% UI)**	**ASR (95% UI)**	**EAPC (95% CI)**
Global	16,141	0.4	19,235	0.24	−1.55
	(14,247–18,034)	(0.35–0.44)	(17,204–21,266)	(0.21–0.27)	(−1.64, −1.47)
Sex					
Male	8,663	0.49	10,468	0.3	−1.56
	(7,657–9,670)	(0.44–0.54)	(9,472–11,464)	(0.27–0.33)	(−1.64, −1.48)
Female	7,478	0.33	8,767	0.2	−1.6
	(6,504–8,452)	(0.29–0.38)	(7,608–9,926)	(0.17–0.22)	(−1.69, −1.51)
Central Europe, Eastern Europe, and Central Asia	824	0.2	841	0.14	−1.51
	(732–916)	(0.18–0.23)	(752–931)	(0.13–0.16)	(−1.79, −1.23)
Central Asia	203	0.3	167	0.22	−1.29
	(174–233)	(0.27–0.34)	(146–187)	(0.19–0.25)	(−1.55, −1.02)
Central Europe	292	0.22	326	0.14	−1.47
	(262–322)	(0.2–0.25)	(289–362)	(0.13–0.16)	(−1.75, −1.19)
Eastern Europe	329	0.14	349	0.1	−1.37
	(285–373)	(0.12–0.16)	(303–396)	(0.09–0.12)	(−1.96, −0.77)
High-income	2,095	0.18	2,472	0.1	−1.7
	(1,844–2,346)	(0.15–0.2)	(2,108–2,837)	(0.08–0.11)	(−1.85, −1.55)
Australasia	26	0.12	37	0.06	−1.87
	(23–29)	(0.11–0.14)	(31–43)	(0.05–0.07)	(−2.14, −1.60)
High-income Asia Pacific	438	0.25	564	0.09	−3.05
	(386–490)	(0.22–0.28)	(461–667)	(0.07–0.1)	(−3.26, −2.85)
High-income North America	552	0.15	646	0.09	−1.49
	(481–623)	(0.13–0.17)	(561–731)	(0.08–0.11)	(−1.63, −1.35)
Southern Latin America	130	0.31	295	0.33	1.3
	(118–142)	(0.28–0.34)	(260–330)	(0.29–0.36)	(0.99,1.61)
Western Europe	949	0.16	930	0.08	−2.1
	(832–1,066)	(0.14–0.18)	(786–1,075)	(0.07–0.09)	(−2.33, −1.88)
Latin America and Caribbean	899	0.42	1,765	0.3	−0.45
	(816–981)	(0.38–0.46)	(1,564–1,967)	(0.26–0.33)	(−0.73, −0.17)
Andean Latin America	158	0.7	235	0.42	−1.21
	(138–178)	(0.61–0.79)	(189–281)	(0.34–0.5)	(−1.42, −1.01)
Caribbean	106	0.4	157	0.3	−0.7
	(93–119)	(0.36–0.45)	(134–181)	(0.25–0.34)	(−0.82, −0.57)
Central Latin America	323	0.38	587	0.25	−0.97
	(296–350)	(0.35–0.41)	(516–658)	(0.22–0.28)	(−1.29, −0.65)
Tropical Latin America	312	0.39	785	0.32	0.34
	(283–340)	(0.35–0.43)	(694–877)	(0.28–0.36)	(−0.01,0.69)
North Africa and Middle East	847	0.41	926	0.24	−1.37
	(726–968)	(0.36–0.46)	(802–1,049)	(0.21–0.27)	(−1.57, −1.16)
South Asia	3,946	0.55	4,744	0.38	−1.15
	(3,292–4,600)	(0.47–0.63)	(4,152–5,336)	(0.33–0.43)	(−1.28, −1.02)
Southeast Asia, East Asia, and Oceania	4,631	0.5	4,796	0.21	−3.03
	(4,096–5,166)	(0.44–0.56)	(4,089–5,504)	(0.17–0.24)	(−3.13, −2.92)
East Asia	3,331	0.52	2,785	0.15	−4.3
	(2,905–3,757)	(0.45–0.59)	(2,212–3,357)	(0.12–0.19)	(−4.48, −4.12)
Oceania	24	0.67	41	0.54	−0.43
	(19–28)	(0.55–0.79)	(33–49)	(0.45–0.63)	(−0.52, −0.33)
Southeast Asia	1,277	0.47	1,973	0.37	−0.51
	(1,112–1,442)	(0.41–0.53)	(1,729–2,217)	(0.32–0.42)	(−0.58, −0.43)
Sub-Saharan Africa	2,894	0.95	3,687	0.75	−0.63
	(2,427–3,362)	(0.82–1.07)	(3,134–4,240)	(0.65–0.85)	(−0.72, −0.53)
Central Sub-Saharan Africa	342	1.16	466	0.97	−0.59
	(267–417)	(0.9–1.43)	(362–570)	(0.74–1.2)	(−0.68, −0.50)
Eastern Sub-Saharan Africa	1,194	1.14	1,306	0.79	−1.21
	(1,001–1,386)	(0.98–1.31)	(1,121–1,490)	(0.68–0.9)	(−1.35, −1.08)
Southern Sub-Saharan Africa	172	0.58	338	0.64	0.97
	(151–193)	(0.51–0.66)	(296–380)	(0.56–0.72)	(0.43,1.52)
Western Sub-Saharan Africa	1,186	0.86	1,578	0.69	−0.53
	(981–1,392)	(0.74–0.98)	(1,274–1,882)	(0.58–0.81)	(−0.62, −0.44)

ASR, age-standardized rate; CI, confidence interval; UI, uncertainty interval; EAPC, estimated annual percentage change.

### 3.2 Age and gender patterns

**Figure 1:**
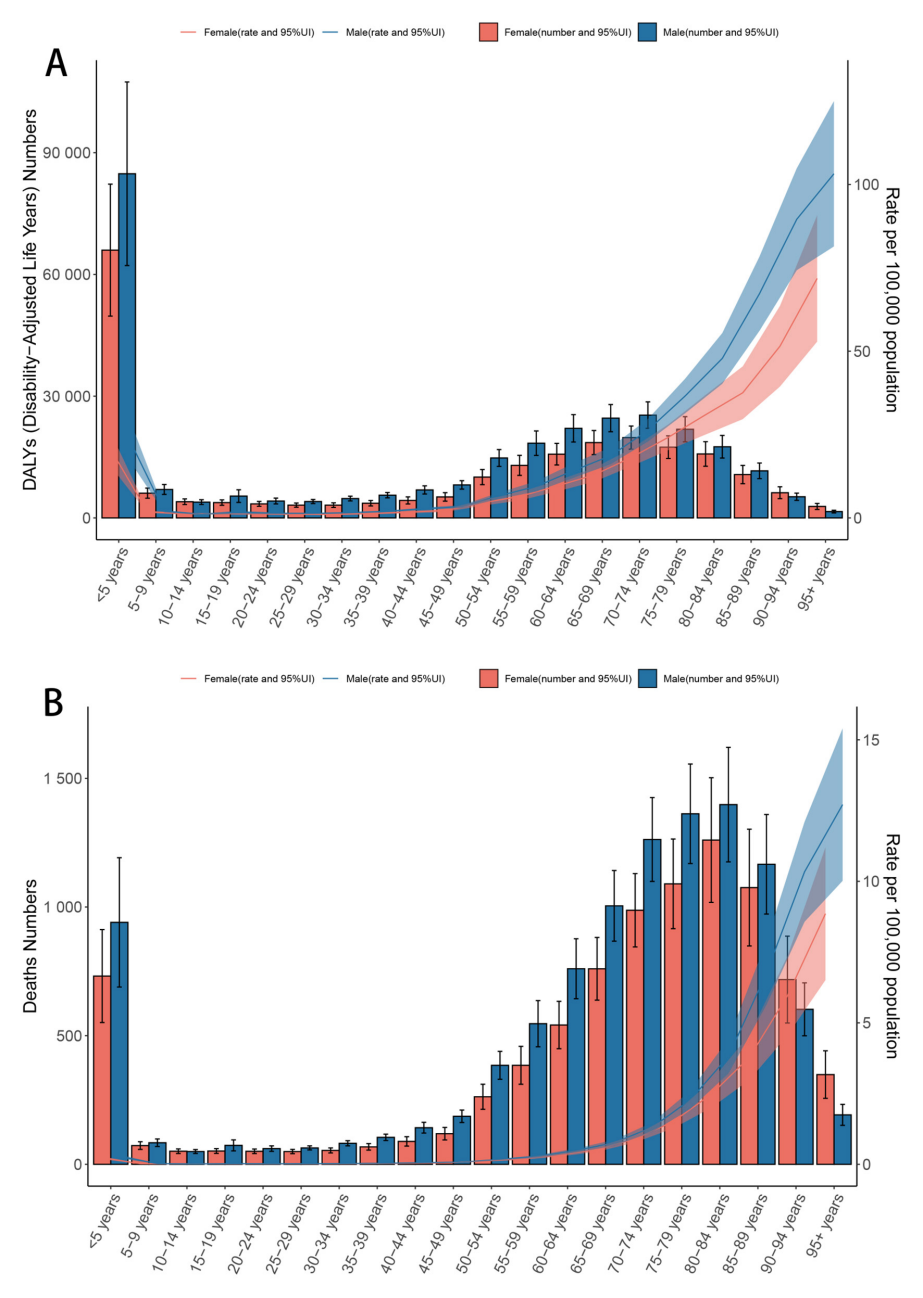
Age-specific numbers and rates of DALYs **(A)** and deaths **(B)** for CMV-associated LRI burden by sex, 2021. Error bars indicate the 95% uncertainty interval for numbers. Shading indicates the 95% uncertainty interval for rates. ASR, Age-standardized rate; DALYs, Disability-adjusted life years; CMV, Cytomegalovirus; LRI, Lower respiratory infections; EAPC, Estimated annual percentage change; SDI, Socio-demographic index; CI, Confidence interval; UI, Uncertainty interval; RR, Relative rate.

Higher age-standardized rates and numbers of DALYs and deaths were observed among both males and females in the under-5 and over-70 age groups (Figure 1, Supplementary Figure S1). For individuals aged over 45 years, DALYs and deaths increased progressively with advancing age. The 70–74 age group recorded the highest number of DALYs (Figure 1A), while the 80–84 group had the highest number of deaths (Figure 1B). Males exhibited higher DALY and death counts than females up to the age of 90 years, after which females accounted for more cases (Figure 1).

### 3.3 Geographical patterns

**Figure 2:**
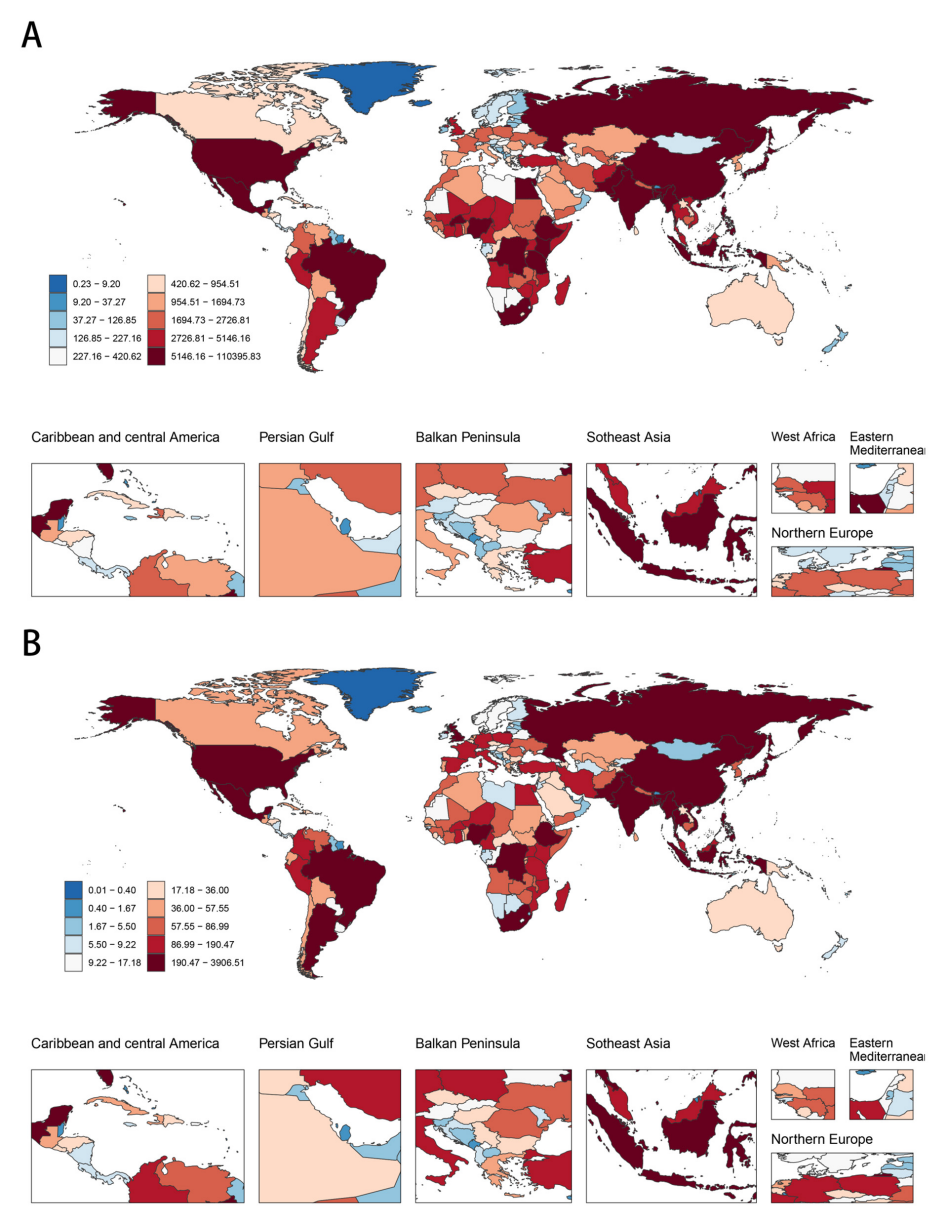
The global disease burden of CMV-associated LRI for both sexes in 204 countries and territories. **(A)** The number of DALYs in 2021. **(B)** The number of deaths in 2021. ASR, Age-standardized rate; DALYs, Disability-adjusted life years; CMV, Cytomegalovirus; LRI, Lower respiratory infections; EAPC, Estimated annual percentage change; SDI, Socio-demographic index; CI, Confidence interval; UI, Uncertainty interval; RR, Relative rate.

In 2021, Oceania and Western Sub-Saharan Africa exhibited the highest age-standardized DALY and death rates attributable to CMV-associated LRIs, whereas Australasia, Western Europe, High-income North America, and the High-income Asia Pacific regions had the lowest rates (Tables 1, 2). In terms of absolute numbers, Western Sub-Saharan Africa and South Asia, particularly countries such as India, China, and Nigeria, bore the greatest burden (Figure 2, Supplementary Table S1).

### 3.4 SDI pattern

**Figure 3:**
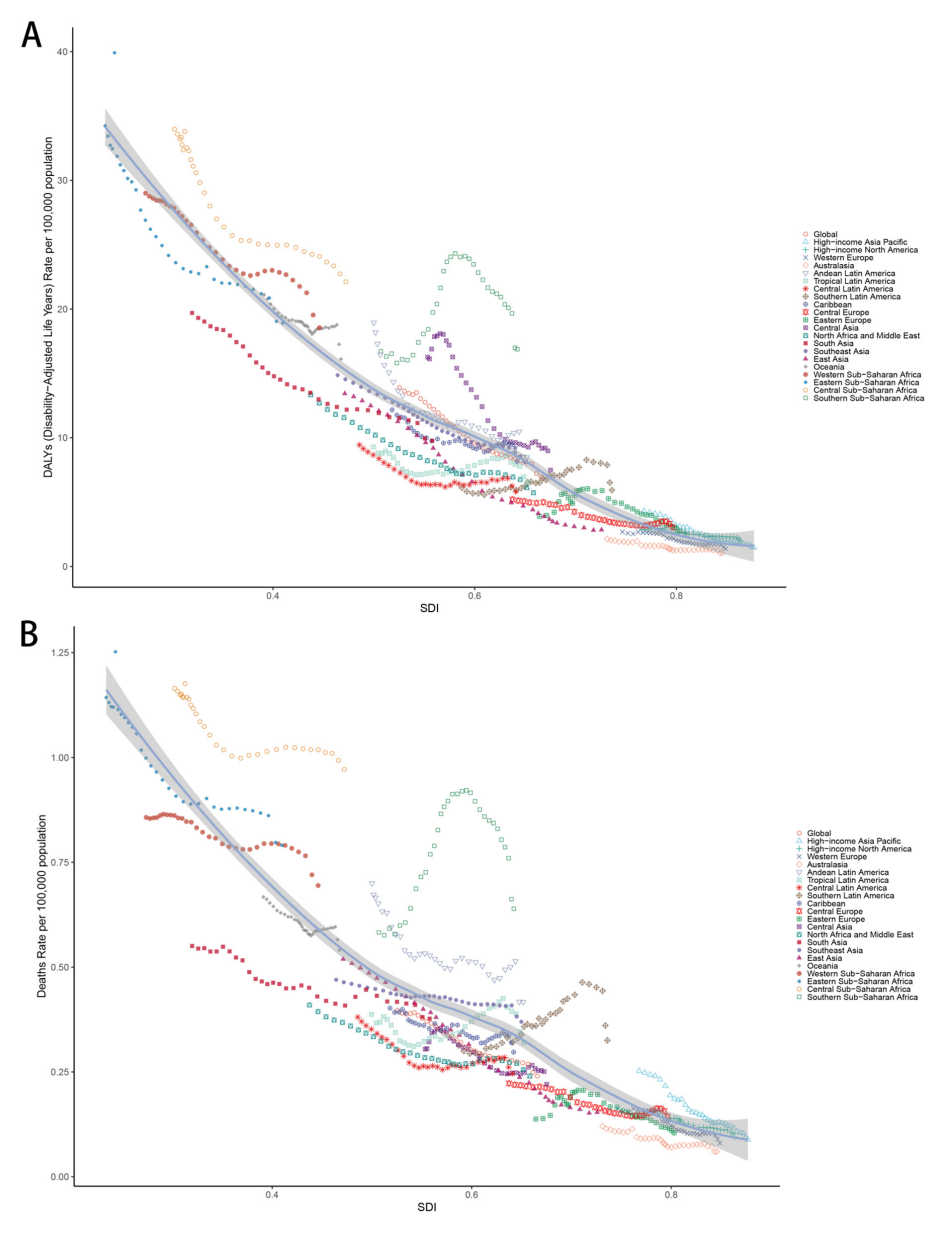
**(A)** Age-standardized DALYs rate and SDI across all regions between 1990 and 2021. **(B)** Age-standardized deaths rate and SDI across all regions between 1990 and 2021. ASR, Age-standardized rate; DALYs, Disability-adjusted life years; CMV, Cytomegalovirus; LRI, Lower respiratory infections; EAPC, Estimated annual percentage change; SDI, Socio-demographic index; CI, Confidence interval; UI, Uncertainty interval; RR, Relative rate.

Age-standardized DALY and death rates were inversely related to SDI levels across all regions from 1990 to 2021 (Figure 3, Supplementary Table S2). In 2021, the highest rates occurred in low SDI regions, while the lowest rates were observed in high SDI regions.

### 3.5 Age, period, and cohort effects

**Figure 4:**
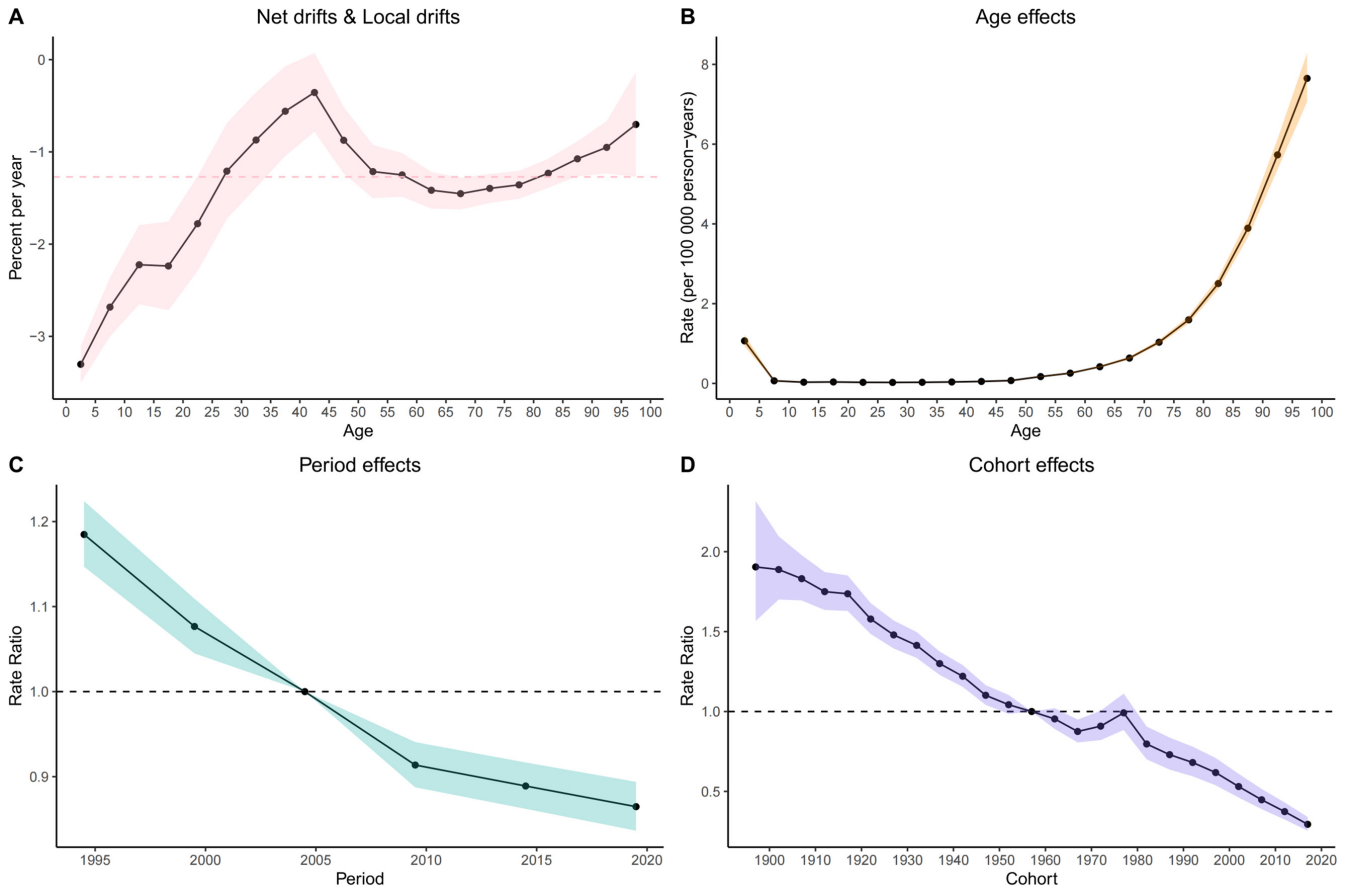
Age, Period, and Cohort Effects on global LRI caused by CMV Mortality: Relative Risk Analysis. **(A)** Net drifts and Local drifts on mortality relative risk. **(B)** Age effects on incidence relative risk. **(C)** Period effects on incidence relative risk. **(D)** Cohort effects on incidence relative risk. ASR, Age-standardized rate; DALYs, Disability-adjusted life years; CMV, Cytomegalovirus; LRI, Lower respiratory infections; EAPC, Estimated annual percentage change; SDI, Socio-demographic index; CI, Confidence interval; UI, Uncertainty interval; RR, Relative rate.

Age-period-cohort (APC) analysis demonstrated a net drift of −1.271 −1.271 (95% CI: −1.429 to −1.114) for mortality rates and −1.278 (95% CI: −1.453 to −1.103) for DALY rates between 1990 and 2021, indicating an overall decreasing trend (Figure 4A; Supplementary Figures S2). After adjustment for period and cohort effects, mortality rates increased steadily with age from the 5–10 year group, with a slow rise under age 59 and a more pronounced exponential increase thereafter (e.g., RR at age 55–60 = 0.26, 95% CI: 0.24–0.27; Figure 4B, Supplementary Table S3). The mortality rate declined from 1994 (RR = 1.18, 95% CI: 1.15–1.22) to 2019 (RR = 0.86, 95% CI: 0.34–0.89), with a notable turning point in 2009 (RR = 0.91, 95% CI: 0.89–0.94), after which the decline was gradual (Figure 4C, Supplementary Table S3). Mortality risk remained in decline until the 1960–1965 birth cohort (RR = 0.86, 95% CI: 0.81–0.95), showed a slight increase from 1965 to 1976, and then returned to a declining trend (Figure 4D, Supplementary Table S3).

### 3.6 Projected burden of CMV-associated LRI from 2021 to 2050

**Figure 5:**
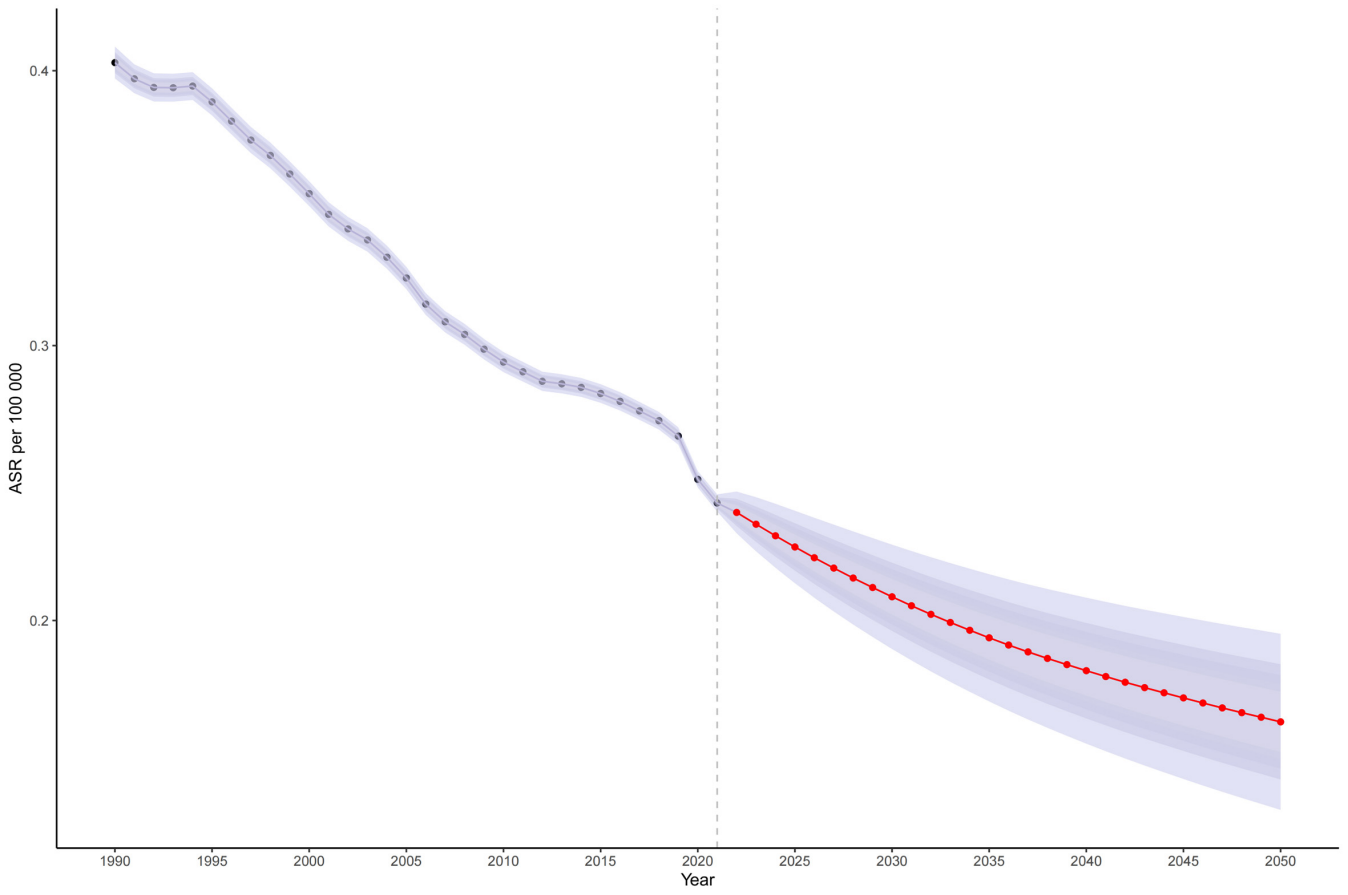
The temporal trend and forecast of LRI caused by CMV globally from 1990 to 2021: Projected age-standardized mortality rate; Red dot lines and shaded regions represent the predicted trend and 95% CI. ASR, Age-standardized rate; DALYs, Disability-adjusted life years; CMV, Cytomegalovirus; LRI, Lower respiratory infections; EAPC, Estimated annual percentage change; SDI, Socio-demographic index; CI, Confidence interval; UI, Uncertainty interval; RR, Relative rate.

Based on projections using the BAPC model and MICROBE database data (1990–2021), the global ASMR for CMV-associated LRIs is expected to decline modestly, from 0.243 per 100,000 in 2021 to 0.163 per 100,000 by 2050 (Figure 5, Supplementary Table S4). The annual number of deaths is projected to continue decreasing after 2021 (Supplementary Figures S2, S3).

## 4 Discussion

Using the latest MICROBE database, we systematically evaluated the global burden and temporal trends of CMV-associated LRIs from 1990 to 2021, stratified by age, sex, geographic location, and socioeconomic status. Although the global burden decreased significantly during this period, the 19,235 deaths (95% UI: 17,204–21,266) recorded in 2021 indicate that CMV-associated LRIs remain an important public health challenge, particularly in low SDI regions and among vulnerable populations such as children and the elderly. These findings provide critical evidence for policymakers to develop targeted prevention and intervention strategies.

In this study, we observed a significant decline in the burden of CMV-associated LRIs from 1990 to 2021, a trend that is likely attributable to advances in both diagnostic and therapeutic modalities for CMV. Over recent decades, real-time quantitative PCR (qPCR) and CRISPR-Cas technologies have greatly enhanced the sensitivity and specificity of CMV detection, particularly in organ transplant recipients and for newborn screening; for example, PCR testing of saliva and urine now achieves sensitivity of 98.8% and specificity of 99.9%, and has become the gold standard for neonatal screening (Ross et al., 2014). Additionally, viral load monitoring has enabled more effective preemptive therapy by guiding the initiation and duration of antiviral treatment, while CMV-specific T-cell immunoassessment allows for individualized treatment regimens and reduced overtreatment (Cui et al., 2022). Therapeutically, the development of novel antiviral agents such as Maribavir, a UL97 kinase inhibitor effective against CMV strains resistant to conventional drugs, has significantly improved outcomes; in phase III trials, 67% of patients with refractory or drug-resistant CMV infections achieved viral clearance (Piret and Boivin, 2024; Papanicolaou et al., 2019). These new agents also offer reduced risks of myelosuppression and nephrotoxicity, improving adherence and safety compared to older treatments such as ganciclovir or phosphonoformate (Piret and Boivin, 2024). Collectively, these diagnostic and therapeutic advances have led to a marked reduction in CMV-related mortality among high-risk populations, enhanced long-term survival, and fewer adverse effects and treatment interruptions associated with traditional antiviral regimens.

Our findings indicate notable population and subgroup differences in the risk factors and burden of CMV-associated LRIs. Both children and older adults experience a disproportionately higher burden of infection, with women over the age of 90 exhibiting greater disease burden than their male counterparts. In children, principal risk factors include inadequate breastfeeding, poor hand hygiene, zinc deficiency, exposure to secondhand smoke, malnutrition, and environmental particulate pollution. For older adults, smoking, alcohol consumption, exposure to secondhand smoke, household solid fuel use, suboptimal hand hygiene, and comorbidities are the predominant risk factors (Troeger et al., 2017). Among individuals aged 45 and older, the increase in DALYs and deaths is closely associated with the age-related decline in T-cell responses caused by immunosenescence (Wang et al., 2022; Lee et al., 2022). The excess burden observed in women after age 90 may be explained by less robust CMV-specific T-cell responses and higher levels of inflammatory markers such as C-reactive protein, which together could impair tissue repair and resilience (Reus et al., 2021). These results highlight the need for targeted public health interventions tailored to high-risk groups, ongoing evaluation of their effectiveness, and continuous monitoring of evolving risk profiles to inform precise, evidence-based strategies for prevention and control.

The burden of CMV-associated LRIs was found to be significantly and inversely correlated with the SDI, highlighting the profound impact of socioeconomic factors on global disease distribution. Regions with lower SDI exhibited markedly higher age-standardized DALY and mortality rates in 2021, and from 1990 to 2021, increases in SDI were associated with steady declines in both metrics. SDI, a composite measure reflecting educational attainment, per capita income, and fertility rates, serves as a broad indicator of a country's socioeconomic status. High SDI regions typically benefit from advanced healthcare infrastructure, which facilitates early detection and timely intervention for CMV infection. Moreover, standardized antiviral therapy (e.g., valganciclovir) has substantially reduced progression rates in these areas, and proactive measures such as newborn CMV screening and maternal antibody testing have decreased vertical transmission rates (Madden et al., 2022; Liberati et al., 2024). Greater educational attainment in high SDI countries also enhances public awareness regarding CMV transmission, in particular mother-to-child and body fluid routes, thereby encouraging healthier behaviors (Aiello et al., 2017). In contrast, low SDI regions frequently experience delayed diagnosis and suboptimal treatment due to resource limitations, which increases the risk of secondary LRI following CMV infection (Sundi et al., 2024; Walabh et al., 2022). Overcrowded living conditions, which is prevalent in many low SDI settings, further amplify the risk of contact transmission of CMV (Aiello et al., 2017). For instance, pediatric liver transplant patients in South African public hospitals demonstrated significantly higher CMV infection rates than those in private facilities, largely attributable to overcrowding (Walabh et al., 2022). Collectively, these findings underscore that low SDI regions bear the greatest burden of CMV-associated LRIs. Effective strategies from high SDI settings, including early screening, precision pharmacotherapy, and health education, must be adapted to the resource constraints and infrastructural challenges characteristic of low SDI regions. Achieving substantial reductions in CMV-related LRI burden, particularly in the most affected areas, will require targeted, collaborative, and multisectoral interventions aimed at achieving greater global health equity.

Projections suggest a continued decline in mortality from CMV-associated LRIs over the next 30 years, a trend that is likely to be accelerated by the broad implementation of new antiviral agents, vaccine development, and public health interventions. Notably, letermovir, a novel antiviral that targets the CMV DNA terminase complex, has been shown to reduce the incidence of clinically significant CMV infection by 83% and decrease the need for antiviral therapy in hematopoietic stem cell transplantation (HSCT) patient (Song, 2024). Furthermore, Moderna's mRNA vaccine V160 has demonstrated robust immunogenicity in phase I clinical trials, eliciting both neutralizing antibody and T-cell responses with a favorable safety profile (Fierro et al., 2024). Additionally, the COVID-19 pandemic highlighted the effectiveness of combined public health measures, such as face mask use, enhanced hand hygiene, and isolation, in significantly reducing respiratory virus transmission (Nunes-Silva et al., 2022). These advances underscore the promise of current pharmaceutical innovation, vaccine technology, and fundamental prevention strategies in reducing the threat of CMV-associated LRIs and moving toward a more controllable disease burden. During the COVID-19 pandemic, the risk of CMV reactivation has been significantly heightened. This reactivation may be associated with COVID-19-induced immunosuppression (Talan et al., 2022), leading to a persistently high rate of CMV infection. Nevertheless, despite these positive projections, certain vulnerable populations, including transplant recipients, cancer patients, and individuals with HIV, may continue to experience a sustained or even increased disease burden. Consequently, it remains essential to strengthen surveillance and targeted management efforts to further decrease mortality in these high-risk groups.

This study has several limitations. First, the limited number of available datasets and the absence of new experimental data constrain the evaluation and reliability of our predictive models (Zou et al., 2017). Additionally, inconsistencies in database management standards and insufficient cross-domain data integration further restrict the depth and scope of our analyses (Gao and Chu, 2020). Second, in regions with low SDI, deficits in medical facilities and human resources, compounded by geographic barriers and inadequate infrastructure, may contribute to a higher incidence of missed or misdiagnosed cases (Shu and Jin, 2023; Osei and Mashamba-Thompson, 2021). Despite these limitations, this study may still provide valuable insights into the temporal trends and overall burden of LRI due to CMV globally, providing an important basis for future research and public health planning.

## 5 Conclusion

This study provides a comprehensive assessment of the global epidemiological patterns and temporal trends of CMV-associated LRIs from 1990 to 2021. Our findings reveal a significant decline in both mortality and DALYs related to CMV-LRIs worldwide, with the most pronounced reductions observed in high SDI regions. Nevertheless, marked disparities persist, particularly affecting children, the elderly, and populations in low SDI regions, where the disease burden remains substantial. These results underscore the critical need to reinforce public health strategies, such as early screening, targeted interventions for high-risk groups, and robust surveillance systems, especially in resource-limited settings, to further reduce the global impact of CMV-associated LRIs and promote greater health equity.

## Data Availability

The datasets presented in this study can be found in online repositories. The names of the repository/repositories and accession number(s) can be found in the article/Supplementary material.
