# Supplementary material for: Global burden of lower respiratory infections attributable to cytomegalovirus, 1990–2021: a systematic analysis from the MICROBE database
Source: Front Microbiol. 2025 Nov 18;16:1693635. doi: 10.3389/fmicb.2025.1693635 (PMC12669215; doi:10.3389/fmicb.2025.1693635)
Supplement: Supplementary file 1 [file Table_1.docx]

Table S1 The global disease burden ofCMV-related LRI for DALYs and deaths number in 204 countries and territories in 2021.

| Location | Measure | Metr ic | Pathogen | Yea r | Value | Upper | Lower |
| --- | --- | --- | --- | --- | --- | --- | --- |
| Afghanistan | DALYs (Disability-Adjusted Life Years) | Numb er | Cytomegalo virus | 202  1 | 4604.81099240 124 | 5643.52934963 0028 | 3566.09263517 2451 |
| Albania | DALYs (Disability-Adjusted Life Years) | Numb er | Cytomegalo virus | 202  1 | 108.231436690 78549 | 129.345897317 01448 | 87.1169760645 5645 |
| Algeria | DALYs (Disability-Adjusted Life Years) | Numb er | Cytomegalo virus | 202  1 | 1280.24164723 92315 | 1508.89572388 50169 | 1051.58757059 3446 |
| American Samoa | DALYs (Disability-Adjusted Life Years) | Numb er | Cytomegalo virus | 202  1 | 3.74616751333 83134 | 4.47023652107 8133 | 3.02209850559 84952 |
| Andorra | DALYs (Disability-Adjusted Life Years) | Numb er | Cytomegalo virus | 202  1 | 1.44632912723 97636 | 1.93840729329 69243 | 0.95425096118 26028 |
| Angola | DALYs (Disability-Adjusted Life Years) | Numb er | Cytomegalo virus | 202  1 | 3383.78209481 5224 | 4291.86425569 8143 | 2475.69993393 23047 |
| Antigua and Barbuda | DALYs (Disability-Adjusted Life Years) | Numb er | Cytomegalo virus | 202  1 | 5.43242491591 0373 | 5.82559643044 0635 | 5.03925340138 011 |
| Argentina | DALYs (Disability-Adjusted Life Years) | Numb er | Cytomegalo virus | 202  1 | 4157.38859929 7834 | 4569.75316895 1053 | 3745.02402964 4614 |
| Armenia | DALYs (Disability-Adjusted Life Years) | Numb er | Cytomegalo virus | 202  1 | 159.849055303 1634 | 180.892337681 75075 | 138.805772924 57605 |
| Australia | DALYs (Disability-Adjusted Life Years) | Numb er | Cytomegalo virus | 202  1 | 445.694421884 7301 | 502.017137844 2303 | 389.371705925 2298 |
| Austria | DALYs (Disability-Adjusted Life Years) | Numb er | Cytomegalo virus | 202  1 | 136.845192450 58456 | 156.201413392 24102 | 117.488971508 92807 |
| Azerbaijan | DALYs (Disability-Adjusted | Numb | Cytomegalo | 202 | 718.592036097 | 845.791127401 | 591.392944793 |

|  | Life Years) | er | virus | 1 | 3273 | 3056 | 349 |
| --- | --- | --- | --- | --- | --- | --- | --- |
| Bahamas | DALYs (Disability-Adjusted Life Years) | Numb er | Cytomegalo virus | 202  1 | 23.8574190305 05213 | 29.0885217604 25863 | 18.6263163005 84563 |
| Bahrain | DALYs (Disability-Adjusted Life Years) | Numb er | Cytomegalo virus | 202  1 | 30.5809516423 17885 | 35.5814837138 118 | 25.5804195708 2397 |
| Bangladesh | DALYs (Disability-Adjusted Life Years) | Numb er | Cytomegalo virus | 202  1 | 10407.1538984 9486 | 12783.6390370 17028 | 8030.66875997 2695 |
| Barbados | DALYs (Disability-Adjusted Life Years) | Numb er | Cytomegalo virus | 202  1 | 24.2399825453 15165 | 29.7149657309 7352 | 18.7649993596 5681 |
| Belarus | DALYs (Disability-Adjusted Life Years) | Numb er | Cytomegalo virus | 202  1 | 368.323128330 2938 | 444.130623459 4383 | 292.515633201 1493 |
| Belgium | DALYs (Disability-Adjusted Life Years) | Numb er | Cytomegalo virus | 202  1 | 430.222583008 3157 | 491.486217425 0148 | 368.958948591 6167 |
| Belize | DALYs (Disability-Adjusted Life Years) | Numb er | Cytomegalo virus | 202  1 | 22.5888729881 9812 | 25.5485222089 01456 | 19.6292237674 94786 |
| Benin | DALYs (Disability-Adjusted Life Years) | Numb er | Cytomegalo virus | 202  1 | 1988.58567526 70093 | 2499.51981973 1616 | 1477.65153080 2404 |
| Bermuda | DALYs (Disability-Adjusted Life Years) | Numb er | Cytomegalo virus | 202  1 | 2.66591008165 04475 | 3.18718769347 3676 | 2.14463246982 7219 |
| Bhutan | DALYs (Disability-Adjusted Life Years) | Numb er | Cytomegalo virus | 202  1 | 34.8534885974 76755 | 44.9042895538 75856 | 24.8026876410 7765 |
| Bolivia (Plurinational State of) | DALYs (Disability-Adjusted Life Years) | Numb er | Cytomegalo virus | 202  1 | 1170.37106151 08012 | 1466.72117002 2157 | 874.020952999 4456 |
| Bosnia and Herzegovina | DALYs (Disability-Adjusted Life Years) | Numb er | Cytomegalo virus | 202  1 | 93.6408641916 6549 | 112.925811036 57932 | 74.3559173467 5164 |
| Botswana | DALYs (Disability-Adjusted | Numb | Cytomegalo | 202 | 230.097844806 | 290.124377796 | 170.071311817 |

|  | Life Years) | er | virus | 1 | 8568 | 53266 | 18086 |
| --- | --- | --- | --- | --- | --- | --- | --- |
| Brazil | DALYs (Disability-Adjusted Life Years) | Numb er | Cytomegalo virus | 202  1 | 15970.9397003 2764 | 17344.7232683 5132 | 14597.1561323 03957 |
| Brunei Darussalam | DALYs (Disability-Adjusted Life Years) | Numb er | Cytomegalo virus | 202  1 | 15.7898373714 72974 | 17.9520616506 4064 | 13.6276130923 05303 |
| Bulgaria | DALYs (Disability-Adjusted Life Years) | Numb er | Cytomegalo virus | 202  1 | 393.166971216 0314 | 454.183032219 1774 | 332.150910212 88544 |
| Burkina Faso | DALYs (Disability-Adjusted Life Years) | Numb er | Cytomegalo virus | 202  1 | 5178.45709857 8256 | 6510.11575812 0839 | 3846.79843903 5672 |
| Burundi | DALYs (Disability-Adjusted Life Years) | Numb er | Cytomegalo virus | 202  1 | 1937.40981222 94183 | 2432.57050205 4494 | 1442.24912240 43429 |
| Cabo Verde | DALYs (Disability-Adjusted Life Years) | Numb er | Cytomegalo virus | 202  1 | 41.3228491849 6956 | 49.9056904521 5975 | 32.7400079177 79366 |
| Cambodia | DALYs (Disability-Adjusted Life Years) | Numb er | Cytomegalo virus | 202  1 | 2554.78531446 9232 | 3199.67340490 5172 | 1909.89722403 32905 |
| Cameroon | DALYs (Disability-Adjusted Life Years) | Numb er | Cytomegalo virus | 202  1 | 3911.52424378 64354 | 4989.78497929 0759 | 2833.26350828 2112 |
| Canada | DALYs (Disability-Adjusted Life Years) | Numb er | Cytomegalo virus | 202  1 | 928.775671855 6108 | 1024.98913448 5229 | 832.562209225 9928 |
| Central African Republic | DALYs (Disability-Adjusted Life Years) | Numb er | Cytomegalo virus | 202  1 | 1457.47125591 45491 | 1863.48490385 22584 | 1051.45760797 684 |
| Chad | DALYs (Disability-Adjusted Life Years) | Numb er | Cytomegalo virus | 202  1 | 4578.14076048 127 | 5910.70847000 3996 | 3245.57305095 85446 |
| Chile | DALYs (Disability-Adjusted Life Years) | Numb er | Cytomegalo virus | 202  1 | 695.099776563 2819 | 758.072640341 2073 | 632.126912785 3566 |
| China | DALYs (Disability-Adjusted | Numb | Cytomegalo | 202 | 48155.9554777 | 58124.3151444 | 38187.5958111 |

|  |  |  | Life Years) | er | virus | 1 | 974 | 74 | 208 |
| --- | --- | --- | --- | --- | --- | --- | --- | --- | --- |
| Colombia |  |  | DALYs (Disability-Adjusted Life Years) | Numb er | Cytomegalo virus | 202  1 | 1949.69767462 9832 | 2301.61473077 98697 | 1597.78061847 97932 |
| Comoros |  |  | DALYs (Disability-Adjusted Life Years) | Numb er | Cytomegalo virus | 202  1 | 91.8263811094 7012 | 111.459419373 8921 | 72.1933428450 4816 |
| Congo |  |  | DALYs (Disability-Adjusted Life Years) | Numb er | Cytomegalo virus | 202  1 | 537.822166729 2778 | 679.530570527 0237 | 396.113762931 532 |
| Cook Islands |  |  | DALYs (Disability-Adjusted Life Years) | Numb er | Cytomegalo virus | 202  1 | 1.35841520176 34537 | 1.59633067687 97958 | 1.12049972664 71115 |
| Costa Rica |  |  | DALYs (Disability-Adjusted Life Years) | Numb er | Cytomegalo virus | 202  1 | 180.267255886 2372 | 203.295605961 85863 | 157.238905810 61576 |
| Croatia |  |  | DALYs (Disability-Adjusted Life Years) | Numb er | Cytomegalo virus | 202  1 | 150.839047077 82594 | 172.231210136 52582 | 129.446884019 12606 |
| Cuba |  |  | DALYs (Disability-Adjusted Life Years) | Numb er | Cytomegalo virus | 202  1 | 954.513703232 4044 | 1083.57346284 31092 | 825.453943621 6998 |
| Cyprus |  |  | DALYs (Disability-Adjusted Life Years) | Numb er | Cytomegalo virus | 202  1 | 27.9626838545 37623 | 32.2364536734 808 | 23.6889140355 94447 |
| Czechia |  |  | DALYs (Disability-Adjusted Life Years) | Numb er | Cytomegalo virus | 202  1 | 452.304336765 8093 | 512.802529239 1649 | 391.806144292 4536 |
| C 么 te d'Ivoire |  |  | DALYs (Disability-Adjusted Life Years) | Numb er | Cytomegalo virus | 202  1 | 4062.47413937 49455 | 5226.50452348 735 | 2898.44375526 25413 |
| Democratic People's Republic of Korea |  |  | DALYs (Disability-Adjusted Life Years) | Numb er | Cytomegalo virus | 202  1 | 1626.72061922 67008 | 1997.81054517 8952 | 1255.63069327 44496 |
| Democratic Republic | of | the | DALYs (Disability-Adjusted | Numb | Cytomegalo | 202 | 12145.0686985 | 15111.0988254 | 9179.03857161 |
| Congo |  |  | Life Years) | er | virus | 1 | 07976 | 01255 | 47 |
| Denmark |  |  | DALYs (Disability-Adjusted | Numb | Cytomegalo | 202 | 215.605222522 | 243.450419006 | 187.760026039 |

|  | Life Years) | er | virus | 1 | 86265 | 6834 | 04183 |
| --- | --- | --- | --- | --- | --- | --- | --- |
| Djibouti | DALYs (Disability-Adjusted Life Years) | Numb er | Cytomegalo virus | 202  1 | 123.309764583 41148 | 161.869703263 09637 | 84.7498259037 266 |
| Dominica | DALYs (Disability-Adjusted Life Years) | Numb er | Cytomegalo virus | 202  1 | 5.38108237685 3588 | 6.30611094980 8733 | 4.45605380389 8442 |
| Dominican Republic | DALYs (Disability-Adjusted Life Years) | Numb er | Cytomegalo virus | 202  1 | 424.506888003 2314 | 521.951927278 1667 | 327.061848728 29607 |
| Ecuador | DALYs (Disability-Adjusted Life Years) | Numb er | Cytomegalo virus | 202  1 | 944.300702306 311 | 1135.17700129 78511 | 753.424403314 7709 |
| Egypt | DALYs (Disability-Adjusted Life Years) | Numb er | Cytomegalo virus | 202  1 | 6054.49832006 5143 | 7429.47178788 7895 | 4679.52485224 2392 |
| El Salvador | DALYs (Disability-Adjusted Life Years) | Numb er | Cytomegalo virus | 202  1 | 389.746140451 8387 | 475.155953473 6184 | 304.336327430 059 |
| Equatorial Guinea | DALYs (Disability-Adjusted Life Years) | Numb er | Cytomegalo virus | 202  1 | 89.6269835412 2918 | 123.892930418 44569 | 55.3610366640 1268 |
| Eritrea | DALYs (Disability-Adjusted Life Years) | Numb er | Cytomegalo virus | 202  1 | 1220.12042957 75265 | 1785.07971753 40124 | 655.161141621 0405 |
| Estonia | DALYs (Disability-Adjusted Life Years) | Numb er | Cytomegalo virus | 202  1 | 43.5884064243 36455 | 49.2660918597 01056 | 37.9107209889 7185 |
| Eswatini | DALYs (Disability-Adjusted Life Years) | Numb er | Cytomegalo virus | 202  1 | 179.312594449 95084 | 234.518801605 3608 | 124.106387294 5409 |
| Ethiopia | DALYs (Disability-Adjusted Life Years) | Numb er | Cytomegalo virus | 202  1 | 10442.7995062 31757 | 12151.7259251 85625 | 8733.87308727 789 |
| Fiji | DALYs (Disability-Adjusted Life Years) | Numb er | Cytomegalo virus | 202  1 | 72.4575998529 7335 | 91.1549426957 0728 | 53.7602570102 3941 |
| Finland | DALYs (Disability-Adjusted | Numb | Cytomegalo | 202 | 112.680153003 | 127.084161272 | 98.2761447339 |

|  | Life Years) | er | virus | 1 | 24484 | 52964 | 6004 |
| --- | --- | --- | --- | --- | --- | --- | --- |
| France | DALYs (Disability-Adjusted Life Years) | Numb er | Cytomegalo virus | 202  1 | 1730.49332037 3681 | 1965.85946022 8125 | 1495.12718051 92368 |
| Gabon | DALYs (Disability-Adjusted Life Years) | Numb er | Cytomegalo virus | 202  1 | 160.210186813 76302 | 210.813585624 59107 | 109.606788002 93496 |
| Gambia | DALYs (Disability-Adjusted Life Years) | Numb er | Cytomegalo virus | 202  1 | 328.013805288 4985 | 397.637854697 1928 | 258.389755879 8041 |
| Georgia | DALYs (Disability-Adjusted Life Years) | Numb er | Cytomegalo virus | 202  1 | 327.259197533 7889 | 363.865881326 6002 | 290.652513740 97755 |
| Germany | DALYs (Disability-Adjusted Life Years) | Numb er | Cytomegalo virus | 202  1 | 2320.90467730 37344 | 2626.38443570 1144 | 2015.42491890 63248 |
| Ghana | DALYs (Disability-Adjusted Life Years) | Numb er | Cytomegalo virus | 202  1 | 3772.11552067 9735 | 4680.04312423 2183 | 2864.18791712 72867 |
| Greece | DALYs (Disability-Adjusted Life Years) | Numb er | Cytomegalo virus | 202  1 | 528.353452995 5259 | 597.622915920 9141 | 459.083990070 1377 |
| Greenland | DALYs (Disability-Adjusted Life Years) | Numb er | Cytomegalo virus | 202  1 | 2.52427189544 6008 | 2.93535773466 2292 | 2.11318605622 9724 |
| Grenada | DALYs (Disability-Adjusted Life Years) | Numb er | Cytomegalo virus | 202  1 | 8.70621143436 0837 | 9.78843009895 368 | 7.62399276976 7993 |
| Guam | DALYs (Disability-Adjusted Life Years) | Numb er | Cytomegalo virus | 202  1 | 6.47697663520 5704 | 7.37245074970 5781 | 5.58150252070 5627 |
| Guatemala | DALYs (Disability-Adjusted Life Years) | Numb er | Cytomegalo virus | 202  1 | 1488.13749962 2558 | 1749.35575021 54606 | 1226.91924902 96552 |
| Guinea | DALYs (Disability-Adjusted Life Years) | Numb er | Cytomegalo virus | 202  1 | 2726.80776736 9227 | 3523.77472319 9788 | 1929.84081153 8666 |
| Guinea-Bissau | DALYs (Disability-Adjusted | Numb | Cytomegalo | 202 | 321.733032021 | 388.442004205 | 255.024059838 |

|  |  |  | Life Years) | er | virus | 1 | 7379 | 1272 | 34865 |
| --- | --- | --- | --- | --- | --- | --- | --- | --- | --- |
| Guyana |  |  | DALYs (Disability-Adjusted Life Years) | Numb er | Cytomegalo virus | 202  1 | 72.0836044957 2196 | 91.7536785716 1616 | 52.4135304198 27765 |
| Haiti |  |  | DALYs (Disability-Adjusted Life Years) | Numb er | Cytomegalo virus | 202  1 | 1822.85426182 49503 | 2334.30549746 25948 | 1311.40302618 73058 |
| Honduras |  |  | DALYs (Disability-Adjusted Life Years) | Numb er | Cytomegalo virus | 202  1 | 655.966935710 2014 | 796.939808545 6495 | 514.994062874 7532 |
| Hungary |  |  | DALYs (Disability-Adjusted Life Years) | Numb er | Cytomegalo virus | 202  1 | 347.343242728 14547 | 394.335216876 1041 | 300.351268580 18685 |
| Iceland |  |  | DALYs (Disability-Adjusted Life Years) | Numb er | Cytomegalo virus | 202  1 | 8.09752695590 182 | 9.37288364534 982 | 6.82217026645 3819 |
| India |  |  | DALYs (Disability-Adjusted Life Years) | Numb er | Cytomegalo virus | 202  1 | 109302.834874 1684 | 125068.971584 9914 | 93536.6981633 4538 |
| Indonesia |  |  | DALYs (Disability-Adjusted Life Years) | Numb er | Cytomegalo virus | 202  1 | 15447.9947596 90442 | 18177.8843690 9234 | 12718.1051502 88548 |
| Iran (Islamic | Republic | of) | DALYs (Disability-Adjusted Life Years) | Numb er | Cytomegalo virus | 202  1 | 1982.05982529 83068 | 2173.58947945 783 | 1790.53017113 87838 |
| Iraq |  |  | DALYs (Disability-Adjusted Life Years) | Numb er | Cytomegalo virus | 202  1 | 1108.67320828 2326 | 1350.47431495 54497 | 866.872101609 2023 |
| Ireland |  |  | DALYs (Disability-Adjusted Life Years) | Numb er | Cytomegalo virus | 202  1 | 109.565014761 52124 | 124.669332076 30878 | 94.4606974467 3372 |
| Israel |  |  | DALYs (Disability-Adjusted Life Years) | Numb er | Cytomegalo virus | 202  1 | 166.866028170 45664 | 187.697075355 78684 | 146.034980985 12645 |
| Italy |  |  | DALYs (Disability-Adjusted Life Years) | Numb er | Cytomegalo virus | 202  1 | 1544.09027569 6671 | 1749.80990521 41615 | 1338.37064617 918 |
| Jamaica |  |  | DALYs (Disability-Adjusted | Numb | Cytomegalo | 202 | 126.845495316 | 157.782092261 | 95.9088983714 |

|  |  | Life Years) | er | virus | 1 | 35722 | 29576 | 1866 |
| --- | --- | --- | --- | --- | --- | --- | --- | --- |
| Japan |  | DALYs (Disability-Adjusted Life Years) | Numb er | Cytomegalo virus | 202  1 | 5961.96590974 88 | 6882.20262782 272 | 5041.72919167 488 |
| Jordan |  | DALYs (Disability-Adjusted Life Years) | Numb er | Cytomegalo virus | 202  1 | 236.866967356 2601 | 284.086095728 00555 | 189.647838984 51452 |
| Kazakhstan |  | DALYs (Disability-Adjusted Life Years) | Numb er | Cytomegalo virus | 202  1 | 978.369193090 1578 | 1093.89146245 84902 | 862.846923721 8254 |
| Kenya |  | DALYs (Disability-Adjusted Life Years) | Numb er | Cytomegalo virus | 202  1 | 5504.46786070 9414 | 6557.40846310 939 | 4451.52725830 94375 |
| Kiribati |  | DALYs (Disability-Adjusted Life Years) | Numb er | Cytomegalo virus | 202  1 | 11.1298256716 21427 | 13.7531383741 88756 | 8.50651296905 4102 |
| Kuwait |  | DALYs (Disability-Adjusted Life Years) | Numb er | Cytomegalo virus | 202  1 | 80.0981511736 2783 | 95.3133263897 0304 | 64.8829759575 526 |
| Kyrgyzstan |  | DALYs (Disability-Adjusted Life Years) | Numb er | Cytomegalo virus | 202  1 | 236.754682944 34492 | 269.458885248 3467 | 204.050480640 34327 |
| Lao People's | Democratic | DALYs (Disability-Adjusted | Numb | Cytomegalo | 202 | 721.185610356 | 904.412194192 | 537.959026521 |
| Republic |  | Life Years) | er | virus | 1 | 9037 | 5696 | 2377 |
| Latvia |  | DALYs (Disability-Adjusted Life Years) | Numb er | Cytomegalo virus | 202  1 | 92.1481811734 4193 | 103.931193202 52714 | 80.3651691443 5668 |
| Lebanon |  | DALYs (Disability-Adjusted Life Years) | Numb er | Cytomegalo virus | 202  1 | 258.921195889 21764 | 299.882703813 9008 | 217.959687964 53448 |
| Lesotho |  | DALYs (Disability-Adjusted Life Years) | Numb er | Cytomegalo virus | 202  1 | 428.180196163 62113 | 531.724648702 786 | 324.635743624 45627 |
| Liberia |  | DALYs (Disability-Adjusted Life Years) | Numb er | Cytomegalo virus | 202  1 | 534.240818424 0381 | 707.284067947 4846 | 361.197568900 5917 |
| Libya |  | DALYs (Disability-Adjusted | Numb | Cytomegalo | 202 | 237.812713665 | 294.827225208 | 180.798202123 |

|  | Life Years) | er | virus | 1 | 85656 | 2542 | 4589 |
| --- | --- | --- | --- | --- | --- | --- | --- |
| Lithuania | DALYs (Disability-Adjusted Life Years) | Numb er | Cytomegalo virus | 202  1 | 114.598814443 28668 | 130.658995704 2122 | 98.5386331823 6114 |
| Luxembourg | DALYs (Disability-Adjusted Life Years) | Numb er | Cytomegalo virus | 202  1 | 12.6355170422 32117 | 14.2580767902 29172 | 11.0129572942 3506 |
| Madagascar | DALYs (Disability-Adjusted Life Years) | Numb er | Cytomegalo virus | 202  1 | 5146.16148725 7556 | 6426.96452503 5237 | 3865.35844947 9874 |
| Malawi | DALYs (Disability-Adjusted Life Years) | Numb er | Cytomegalo virus | 202  1 | 2661.32128039 11188 | 3315.75003859 0485 | 2006.89252219 1752 |
| Malaysia | DALYs (Disability-Adjusted Life Years) | Numb er | Cytomegalo virus | 202  1 | 3212.57439280 6729 | 3630.86061278 19694 | 2794.28817283 14883 |
| Maldives | DALYs (Disability-Adjusted Life Years) | Numb er | Cytomegalo virus | 202  1 | 9.19969479407 8418 | 10.9120178833 18695 | 7.48737170483 8139 |
| Mali | DALYs (Disability-Adjusted Life Years) | Numb er | Cytomegalo virus | 202  1 | 3308.45797812 5989 | 4035.33837547 14814 | 2581.57758078 04966 |
| Malta | DALYs (Disability-Adjusted Life Years) | Numb er | Cytomegalo virus | 202  1 | 15.2667880481 77355 | 17.5372654593 3583 | 12.9963106370 1888 |
| Marshall Islands | DALYs (Disability-Adjusted Life Years) | Numb er | Cytomegalo virus | 202  1 | 6.45448839988 9637 | 8.07463849020 7215 | 4.83433830957 2059 |
| Mauritania | DALYs (Disability-Adjusted Life Years) | Numb er | Cytomegalo virus | 202  1 | 420.618147352 7792 | 515.252330809 952 | 325.983963895 6063 |
| Mauritius | DALYs (Disability-Adjusted Life Years) | Numb er | Cytomegalo virus | 202  1 | 72.0635322935 7435 | 77.6578025508 8876 | 66.4692620362 5995 |
| Mexico | DALYs (Disability-Adjusted Life Years) | Numb er | Cytomegalo virus | 202  1 | 7615.42835548 0217 | 8598.13996243 5882 | 6632.71674852 4553 |
| Micronesia (Federated | DALYs (Disability-Adjusted | Numb | Cytomegalo | 202 | 10.8986157772 | 13.6856528220 | 8.11157873252 |

| States of) | Life Years) | er | virus | 1 | 6294 | 015 | 438 |
| --- | --- | --- | --- | --- | --- | --- | --- |
| Monaco | DALYs (Disability-Adjusted Life Years) | Numb er | Cytomegalo virus | 202  1 | 1.50444615218 66332 | 1.80877209825 56243 | 1.20012020611 7642 |
| Mongolia | DALYs (Disability-Adjusted Life Years) | Numb er | Cytomegalo virus | 202  1 | 165.186947284 41994 | 194.340051897 01088 | 136.033842671 829 |
| Montenegro | DALYs (Disability-Adjusted Life Years) | Numb er | Cytomegalo virus | 202  1 | 21.5942065129 1022 | 25.0247192950 42367 | 18.1636937307 7807 |
| Morocco | DALYs (Disability-Adjusted Life Years) | Numb er | Cytomegalo virus | 202  1 | 1979.47382823 6993 | 2414.96971304 91936 | 1543.97794342 47928 |
| Mozambique | DALYs (Disability-Adjusted Life Years) | Numb er | Cytomegalo virus | 202  1 | 3967.52270122 72017 | 4873.01172137 1347 | 3062.03368108 3056 |
| Myanmar | DALYs (Disability-Adjusted Life Years) | Numb er | Cytomegalo virus | 202  1 | 5995.50074898 3272 | 7210.65884603 1033 | 4780.34265193 551 |
| Namibia | DALYs (Disability-Adjusted Life Years) | Numb er | Cytomegalo virus | 202  1 | 279.013951964 6624 | 374.431836189 28816 | 183.596067740 03663 |
| Nauru | DALYs (Disability-Adjusted Life Years) | Numb er | Cytomegalo virus | 202  1 | 1.30579532871 3801 | 1.65982531561 22875 | 0.95176534181 53148 |
| Nepal | DALYs (Disability-Adjusted Life Years) | Numb er | Cytomegalo virus | 202  1 | 2460.72916018 2062 | 3028.71758404 35825 | 1892.74073632 05416 |
| Netherlands | DALYs (Disability-Adjusted Life Years) | Numb er | Cytomegalo virus | 202  1 | 533.162523522 3972 | 598.525064556 1096 | 467.799982488 685 |
| New Zealand | DALYs (Disability-Adjusted Life Years) | Numb er | Cytomegalo virus | 202  1 | 107.524766818 34602 | 119.920999362 533 | 95.1285342741 5904 |
| Nicaragua | DALYs (Disability-Adjusted Life Years) | Numb er | Cytomegalo virus | 202  1 | 243.442455564 67908 | 284.936695437 55175 | 201.948215691 8064 |
| Niger | DALYs (Disability-Adjusted | Numb | Cytomegalo | 202 | 5108.17177548 | 6576.93814327 | 3639.40540768 |

|  |  | Life Years) | er | virus | 1 | 026 | 0633 | 9888 |
| --- | --- | --- | --- | --- | --- | --- | --- | --- |
| Nigeria |  | DALYs (Disability-Adjusted Life Years) | Numb er | Cytomegalo virus | 202  1 | 38659.1973114 77415 | 49261.7717477 2836 | 28056.6228752 2648 |
| Niue |  | DALYs (Disability-Adjusted Life Years) | Numb er | Cytomegalo virus | 202  1 | 0.23537350352 92946 | 0.26882090194 85157 | 0.20192610511 00736 |
| North Macedonia |  | DALYs (Disability-Adjusted Life Years) | Numb er | Cytomegalo virus | 202  1 | 80.7201777538 3084 | 97.1955000778 4368 | 64.2448554298 1799 |
| Northern Mariana | Islands | DALYs (Disability-Adjusted Life Years) | Numb er | Cytomegalo virus | 202  1 | 2.59447930551 96578 | 2.87189305481 7147 | 2.31706555622 21687 |
| Norway |  | DALYs (Disability-Adjusted Life Years) | Numb er | Cytomegalo virus | 202  1 | 134.682959096 24615 | 153.407405178 47493 | 115.958513014 01736 |
| Oman |  | DALYs (Disability-Adjusted Life Years) | Numb er | Cytomegalo virus | 202  1 | 87.4966426831 972 | 103.974897967 19256 | 71.0183873992 0183 |
| Pakistan |  | DALYs (Disability-Adjusted Life Years) | Numb er | Cytomegalo virus | 202  1 | 19595.1882186 39363 | 23484.2303968 5325 | 15706.1460404 25472 |
| Palau |  | DALYs (Disability-Adjusted Life Years) | Numb er | Cytomegalo virus | 202  1 | 1.71253822551 05626 | 2.05412903884 8011 | 1.37094741217 3114 |
| Palestine |  | DALYs (Disability-Adjusted Life Years) | Numb er | Cytomegalo virus | 202  1 | 156.315278323 9155 | 178.125726298 6362 | 134.504830349 19478 |
| Panama |  | DALYs (Disability-Adjusted Life Years) | Numb er | Cytomegalo virus | 202  1 | 149.849148961 09638 | 180.834721045 7065 | 118.863576876 48628 |
| Papua New Guinea |  | DALYs (Disability-Adjusted Life Years) | Numb er | Cytomegalo virus | 202  1 | 1694.72637329 2482 | 2148.88609788 4113 | 1240.56664870 08502 |
| Paraguay |  | DALYs (Disability-Adjusted Life Years) | Numb er | Cytomegalo virus | 202  1 | 317.313444208 15066 | 393.330806833 5367 | 241.296081582 7646 |
| Peru |  | DALYs (Disability-Adjusted | Numb | Cytomegalo | 202 | 2918.76361889 | 3611.92496002 | 2225.60227775 |

|  | Life Years) | er | virus | 1 | 4818 | 97912 | 9845 |
| --- | --- | --- | --- | --- | --- | --- | --- |
| Philippines | DALYs (Disability-Adjusted Life Years) | Numb er | Cytomegalo virus | 202  1 | 10502.8246517 68686 | 12111.0734548 312 | 8894.57584870 617 |
| Poland | DALYs (Disability-Adjusted Life Years) | Numb er | Cytomegalo virus | 202  1 | 2250.31803463 29145 | 2490.87220163 01504 | 2009.76386763 56787 |
| Portugal | DALYs (Disability-Adjusted Life Years) | Numb er | Cytomegalo virus | 202  1 | 576.817620901 7662 | 648.510855103 7962 | 505.124386699 7362 |
| Puerto Rico | DALYs (Disability-Adjusted Life Years) | Numb er | Cytomegalo virus | 202  1 | 168.962548632 00815 | 200.942442930 58333 | 136.982654333 43298 |
| Qatar | DALYs (Disability-Adjusted Life Years) | Numb er | Cytomegalo virus | 202  1 | 21.7118153332 3192 | 27.0497544754 4934 | 16.3738761910 14496 |
| Republic of Korea | DALYs (Disability-Adjusted Life Years) | Numb er | Cytomegalo virus | 202  1 | 1271.92753705 66486 | 1470.79084898 64433 | 1073.06422512 6854 |
| Republic of Moldova | DALYs (Disability-Adjusted Life Years) | Numb er | Cytomegalo virus | 202  1 | 224.543486710 16453 | 252.905742851 49373 | 196.181230568 83533 |
| Romania | DALYs (Disability-Adjusted Life Years) | Numb er | Cytomegalo virus | 202  1 | 1273.19877572 106 | 1422.01417098 98814 | 1124.38338045 22385 |
| Russian Federation | DALYs (Disability-Adjusted Life Years) | Numb er | Cytomegalo virus | 202  1 | 5460.73107642 1052 | 6095.31832876 2028 | 4826.14382408 0077 |
| Rwanda | DALYs (Disability-Adjusted Life Years) | Numb er | Cytomegalo virus | 202  1 | 1406.40123355 58973 | 1782.75267379 5069 | 1030.04979331 67255 |
| Saint Kitts and Nevis | DALYs (Disability-Adjusted Life Years) | Numb er | Cytomegalo virus | 202  1 | 4.23681533757 7039 | 4.94543258716 9154 | 3.52819808798 49244 |
| Saint Lucia | DALYs (Disability-Adjusted Life Years) | Numb er | Cytomegalo virus | 202  1 | 12.3709608055 093 | 14.8452006354 98166 | 9.89672097552 0434 |
| Saint Vincent and the | DALYs (Disability-Adjusted | Numb | Cytomegalo | 202 | 9.47699668657 | 10.7877193665 | 8.16627400659 |

| Grenadines | Life Years) | er | virus | 1 | 6877 | 5679 | 696 |
| --- | --- | --- | --- | --- | --- | --- | --- |
| Samoa | DALYs (Disability-Adjusted Life Years) | Numb er | Cytomegalo virus | 202  1 | 15.9741385505 07904 | 19.1237451015 90497 | 12.8245319994 25313 |
| San Marino | DALYs (Disability-Adjusted Life Years) | Numb er | Cytomegalo virus | 202  1 | 0.51557268444 6056 | 0.69940269372 61635 | 0.33174267516 59484 |
| Sao Tome and Principe | DALYs (Disability-Adjusted Life Years) | Numb er | Cytomegalo virus | 202  1 | 22.4112692033 5828 | 27.3582979501 8379 | 17.4642404565 3277 |
| Saudi Arabia | DALYs (Disability-Adjusted Life Years) | Numb er | Cytomegalo virus | 202  1 | 1117.39664661 64911 | 1407.92945491 87553 | 826.863838314 227 |
| Senegal | DALYs (Disability-Adjusted Life Years) | Numb er | Cytomegalo virus | 202  1 | 1873.43290316 54447 | 2284.30472913 3133 | 1462.56107719 77565 |
| Serbia | DALYs (Disability-Adjusted Life Years) | Numb er | Cytomegalo virus | 202  1 | 423.008809239 17634 | 491.338495422 9301 | 354.679123055 42256 |
| Seychelles | DALYs (Disability-Adjusted Life Years) | Numb er | Cytomegalo virus | 202  1 | 9.634985053 | 10.9338046667 04145 | 8.33616543976 1858 |
| Sierra Leone | DALYs (Disability-Adjusted Life Years) | Numb er | Cytomegalo virus | 202  1 | 1246.25549599 7966 | 1613.31977271 5491 | 879.191219280 441 |
| Singapore | DALYs (Disability-Adjusted Life Years) | Numb er | Cytomegalo virus | 202  1 | 227.762757826 42583 | 255.756224584 15245 | 199.769291068 6992 |
| Slovakia | DALYs (Disability-Adjusted Life Years) | Numb er | Cytomegalo virus | 202  1 | 240.640482946 47537 | 277.464876849 27723 | 203.816089043 6735 |
| Slovenia | DALYs (Disability-Adjusted Life Years) | Numb er | Cytomegalo virus | 202  1 | 58.3471499643 1423 | 67.1076603541 9809 | 49.5866395744 3036 |
| Solomon Islands | DALYs (Disability-Adjusted Life Years) | Numb er | Cytomegalo virus | 202  1 | 127.069355684 488 | 156.450151333 7027 | 97.6885600352 7333 |
| Somalia | DALYs (Disability-Adjusted | Numb | Cytomegalo | 202 | 4141.56010077 | 5345.36937252 | 2937.75082903 |

|  | Life Years) | er | virus | 1 | 8892 | 2289 | 5495 |
| --- | --- | --- | --- | --- | --- | --- | --- |
| South Africa | DALYs (Disability-Adjusted Life Years) | Numb er | Cytomegalo virus | 202  1 | 6622.52748288 9598 | 7322.71265593 6399 | 5922.34230984 27975 |
| South Sudan | DALYs (Disability-Adjusted Life Years) | Numb er | Cytomegalo virus | 202  1 | 1892.71943418 43544 | 2569.29741962 0789 | 1216.14144874 79192 |
| Spain | DALYs (Disability-Adjusted Life Years) | Numb er | Cytomegalo virus | 202  1 | 1277.58288370 32334 | 1449.17021163 79606 | 1105.99555576 85062 |
| Sri Lanka | DALYs (Disability-Adjusted Life Years) | Numb er | Cytomegalo virus | 202  1 | 790.117779585 6039 | 1052.62294299 59706 | 527.612616175 2372 |
| Sudan | DALYs (Disability-Adjusted Life Years) | Numb er | Cytomegalo virus | 202  1 | 1998.48517348 13792 | 2578.08356793 932 | 1418.88677902 34383 |
| Suriname | DALYs (Disability-Adjusted Life Years) | Numb er | Cytomegalo virus | 202  1 | 37.2749544627 4705 | 47.4950864497 2019 | 27.0548224757 739 |
| Sweden | DALYs (Disability-Adjusted Life Years) | Numb er | Cytomegalo virus | 202  1 | 223.452671010 438 | 255.232097796 3582 | 191.673244224 51784 |
| Switzerland | DALYs (Disability-Adjusted Life Years) | Numb er | Cytomegalo virus | 202  1 | 160.350334470 1146 | 183.053998535 4596 | 137.646670404 76965 |
| Syrian Arab Republic | DALYs (Disability-Adjusted Life Years) | Numb er | Cytomegalo virus | 202  1 | 592.797314213 6843 | 742.845084191 1073 | 442.749544236 2614 |
| Taiwan (Province of China) | DALYs (Disability-Adjusted Life Years) | Numb er | Cytomegalo virus | 202  1 | 1208.07207376 05516 | 1346.94856917 37916 | 1069.19557834 73115 |
| Tajikistan | DALYs (Disability-Adjusted Life Years) | Numb er | Cytomegalo virus | 202  1 | 1087.53576181 3677 | 1414.19730892 1567 | 760.874214705 7874 |
| Thailand | DALYs (Disability-Adjusted Life Years) | Numb er | Cytomegalo virus | 202  1 | 4817.00386853 6428 | 6058.88993982 616 | 3575.11779724 6696 |
| Timor-Leste | DALYs (Disability-Adjusted | Numb | Cytomegalo | 202 | 133.410464298 | 160.639937371 | 106.180991225 |

|  | Life Years) | er | virus | 1 | 6706 | 77242 | 5688 |
| --- | --- | --- | --- | --- | --- | --- | --- |
| Togo | DALYs (Disability-Adjusted Life Years) | Numb er | Cytomegalo virus | 202  1 | 1216.00439772 1186 | 1556.51834117 88307 | 875.490454263 5413 |
| Tokelau | DALYs (Disability-Adjusted Life Years) | Numb er | Cytomegalo virus | 202  1 | 0.13996644490 45427 | 0.16390889140 86141 | 0.11602399840 04712 |
| Tonga | DALYs (Disability-Adjusted Life Years) | Numb er | Cytomegalo virus | 202  1 | 8.91168679434 7446 | 11.0913358341 28 | 6.73203775456 6891 |
| Trinidad and Tobago | DALYs (Disability-Adjusted Life Years) | Numb er | Cytomegalo virus | 202  1 | 83.0431136896 1442 | 104.401747850 41098 | 61.6844795288 17846 |
| Tunisia | DALYs (Disability-Adjusted Life Years) | Numb er | Cytomegalo virus | 202  1 | 350.982358680 4794 | 453.564703005 4699 | 248.400014355 48896 |
| Turkmenistan | DALYs (Disability-Adjusted Life Years) | Numb er | Cytomegalo virus | 202  1 | 374.237930987 2289 | 442.631136032 7685 | 305.844725941 68926 |
| Tuvalu | DALYs (Disability-Adjusted Life Years) | Numb er | Cytomegalo virus | 202  1 | 1.20198288237 2315 | 1.41239997932 15871 | 0.99156578542 30436 |
| T 眉 rkiye | DALYs (Disability-Adjusted Life Years) | Numb er | Cytomegalo virus | 202  1 | 2858.09254395 9121 | 3386.39113580 1565 | 2329.79395211 6678 |
| Uganda | DALYs (Disability-Adjusted Life Years) | Numb er | Cytomegalo virus | 202  1 | 4070.08169009 7833 | 4956.29975054 4393 | 3183.86362965 12727 |
| Ukraine | DALYs (Disability-Adjusted Life Years) | Numb er | Cytomegalo virus | 202  1 | 1796.30178121 6015 | 2340.56518883 91703 | 1252.03837359 28588 |
| United Arab Emirates | DALYs (Disability-Adjusted Life Years) | Numb er | Cytomegalo virus | 202  1 | 130.180590595 19032 | 156.759797344 1109 | 103.601383846 26975 |
| United Kingdom | DALYs (Disability-Adjusted Life Years) | Numb er | Cytomegalo virus | 202  1 | 2949.82528994 4672 | 3284.65118492 8952 | 2614.99939496 0392 |
| United Republic of Tanzania | DALYs (Disability-Adjusted | Numb | Cytomegalo | 202 | 7299.59068581 | 9004.39989523 | 5594.78147638 |

|  | Life Years) | er | virus | 1 | 2445 | 8374 | 6517 |
| --- | --- | --- | --- | --- | --- | --- | --- |
| United States of America | DALYs (Disability-Adjusted Life Years) | Numb er | Cytomegalo virus | 202  1 | 11051.1012948 66416 | 12153.2524542 79693 | 9948.95013545 3144 |
| United States Virgin Islands | DALYs (Disability-Adjusted Life Years) | Numb er | Cytomegalo virus | 202  1 | 3.84047697744 5434 | 4.72630132576 3669 | 2.95465262912 71995 |
| Uruguay | DALYs (Disability-Adjusted Life Years) | Numb er | Cytomegalo virus | 202  1 | 227.161745773 70708 | 249.705840114 5133 | 204.617651432 90089 |
| Uzbekistan | DALYs (Disability-Adjusted Life Years) | Numb er | Cytomegalo virus | 202  1 | 2656.92217574 0554 | 3166.16783856 5045 | 2147.67651291 6063 |
| Vanuatu | DALYs (Disability-Adjusted Life Years) | Numb er | Cytomegalo virus | 202  1 | 26.9301862707 0699 | 31.4067304517 3743 | 22.4536420896 7655 |
| Venezuela (Bolivarian Republic of) | DALYs (Disability-Adjusted Life Years) | Numb er | Cytomegalo virus | 202  1 | 1514.73467581 83503 | 1925.43973187 90195 | 1104.02961975 76813 |
| Viet Nam | DALYs (Disability-Adjusted Life Years) | Numb er | Cytomegalo virus | 202  1 | 4536.22813297 7492 | 5457.17970006 5082 | 3615.27656588 9902 |
| Yemen | DALYs (Disability-Adjusted Life Years) | Numb er | Cytomegalo virus | 202  1 | 2562.86134466 40547 | 3169.95286729 6076 | 1955.76982203 20333 |
| Zambia | DALYs (Disability-Adjusted Life Years) | Numb er | Cytomegalo virus | 202  1 | 2340.53506820 18925 | 2993.19585401 67616 | 1687.87428238 70234 |
| Zimbabwe | DALYs (Disability-Adjusted Life Years) | Numb er | Cytomegalo virus | 202  1 | 3307.65073041 92714 | 4090.28978261 743 | 2525.01167822 1113 |
| Afghanistan | Deaths | Numb er | Cytomegalo virus | 202  1 | 83.8383207989 4175 | 99.9078957463 1056 | 67.7687458515 7294 |
| Albania | Deaths | Numb er | Cytomegalo virus | 202  1 | 5.90228227829 5591 | 7.06821296134 9596 | 4.73635159524 1585 |
| Algeria | Deaths | Numb | Cytomegalo | 202 | 55.2061592724 | 65.4702883641 | 44.9420301807 |

|  |  | er | virus | 1 | 3362 | 0443 | 6282 |
| --- | --- | --- | --- | --- | --- | --- | --- |
| American Samoa | Deaths | Numb er | Cytomegalo virus | 202  1 | 0.14896965177 14457 | 0.17456719056 12984 | 0.12337211298 1593 |
| Andorra | Deaths | Numb er | Cytomegalo virus | 202  1 | 0.08997006780 29348 | 0.12013077403 13835 | 0.05980936157 44861 |
| Angola | Deaths | Numb er | Cytomegalo virus | 202  1 | 83.6241022722 6543 | 103.740339713 274 | 63.5078648312 56846 |
| Antigua and Barbuda | Deaths | Numb er | Cytomegalo virus | 202  1 | 0.27192426887 2355 | 0.29608286612 52681 | 0.24776567161 94418 |
| Argentina | Deaths | Numb er | Cytomegalo virus | 202  1 | 241.878083746 2316 | 270.707702028 0821 | 213.048465464 38108 |
| Armenia | Deaths | Numb er | Cytomegalo virus | 202  1 | 8.07627045561 5457 | 9.23656996104 462 | 6.91597095018 62965 |
| Australia | Deaths | Numb er | Cytomegalo virus | 202  1 | 29.7426035417 87407 | 34.4781146853 21444 | 25.0070923982 5337 |
| Austria | Deaths | Numb er | Cytomegalo virus | 202  1 | 9.95355633275 9664 | 11.6439118897 87065 | 8.26320077573 2264 |
| Azerbaijan | Deaths | Numb er | Cytomegalo virus | 202  1 | 17.8740376537 7008 | 20.4705739096 41743 | 15.2775013978 98416 |
| Bahamas | Deaths | Numb er | Cytomegalo virus | 202  1 | 1.02396435152 0354 | 1.23021927663 1724 | 0.81770942640 89841 |
| Bahrain | Deaths | Numb er | Cytomegalo virus | 202  1 | 1.12269779179 1771 | 1.30779985458 323 | 0.93759572900 0312 |
| Bangladesh | Deaths | Numb er | Cytomegalo virus | 202  1 | 356.065128894 36004 | 433.423233881 4054 | 278.707023907 3147 |
| Barbados | Deaths | Numb | Cytomegalo | 202 | 1.38809196081 | 1.67284915031 | 1.10333477131 |

|  |  | er | virus | 1 | 41054 | 47649 | 34458 |
| --- | --- | --- | --- | --- | --- | --- | --- |
| Belarus | Deaths | Numb er | Cytomegalo virus | 202  1 | 15.2062397785 817 | 18.3740817458 1508 | 12.0383978113 4832 |
| Belgium | Deaths | Numb er | Cytomegalo virus | 202  1 | 30.3104105788 7956 | 35.6557364579 4508 | 24.9650846998 14035 |
| Belize | Deaths | Numb er | Cytomegalo virus | 202  1 | 0.90908997013 3665 | 1.03166520488 02182 | 0.78651473538 71118 |
| Benin | Deaths | Numb er | Cytomegalo virus | 202  1 | 44.4956236668 5805 | 54.2420379795 7206 | 34.7492093541 4404 |
| Bermuda | Deaths | Numb er | Cytomegalo virus | 202  1 | 0.16538891242 3017 | 0.19810024410 83653 | 0.13267758073 76686 |
| Bhutan | Deaths | Numb er | Cytomegalo virus | 202  1 | 1.42922170590 166 | 1.80693486585 92377 | 1.05150854594 40825 |
| Bolivia (Plurinational State of) | Deaths | Numb er | Cytomegalo virus | 202  1 | 46.3007554890 2998 | 57.2337714508 3729 | 35.3677395272 22675 |
| Bosnia and Herzegovina | Deaths | Numb er | Cytomegalo virus | 202  1 | 5.50260550618 4921 | 6.72185280889 7822 | 4.28335820347 202 |
| Botswana | Deaths | Numb er | Cytomegalo virus | 202  1 | 7.24162672166 5091 | 9.04307336512 1966 | 5.44018007820 8217 |
| Brazil | Deaths | Numb er | Cytomegalo virus | 202  1 | 770.752548642 9813 | 860.619003249 7748 | 680.886094036 1878 |
| Brunei Darussalam | Deaths | Numb er | Cytomegalo virus | 202  1 | 0.67558369133 6771 | 0.77406837617 99378 | 0.57709900649 36041 |
| Bulgaria | Deaths | Numb er | Cytomegalo virus | 202  1 | 21.5101576069 7153 | 25.0004592649 5372 | 18.0198559489 89346 |
| Burkina Faso | Deaths | Numb | Cytomegalo | 202 | 104.845756066 | 127.245783823 | 82.4457283105 |

|  |  |  | er | virus | 1 | 99164 | 41318 | 701 |
| --- | --- | --- | --- | --- | --- | --- | --- | --- |
| Burundi |  | Deaths | Numb er | Cytomegalo virus | 202  1 | 46.5644866088 854 | 56.8933335003 9315 | 36.2356397173 7766 |
| Cabo Verde |  | Deaths | Numb er | Cytomegalo virus | 202  1 | 2.02112553496 1792 | 2.38650378397 06784 | 1.65574728595 29043 |
| Cambodia |  | Deaths | Numb er | Cytomegalo virus | 202  1 | 83.3509221935 5725 | 102.030981671 2665 | 64.6708627158 48 |
| Cameroon |  | Deaths | Numb er | Cytomegalo virus | 202  1 | 91.7245011268 8377 | 116.796114463 66344 | 66.6528877901 0407 |
| Canada |  | Deaths | Numb er | Cytomegalo virus | 202  1 | 56.6856048939 5277 | 64.3484336749 049 | 49.0227761130 00646 |
| Central African | Republic | Deaths | Numb er | Cytomegalo virus | 202  1 | 30.3686594870 33945 | 38.2797488500 2455 | 22.4575701240 4334 |
| Chad |  | Deaths | Numb er | Cytomegalo virus | 202  1 | 80.9880699779 5434 | 101.787125359 61252 | 60.1890145962 9615 |
| Chile |  | Deaths | Numb er | Cytomegalo virus | 202  1 | 39.1839874301 3862 | 43.8470949304 7151 | 34.5208799298 0574 |
| China |  | Deaths | Numb er | Cytomegalo virus | 202  1 | 2630.70967946 23653 | 3192.02560013 1098 | 2069.39375879 36325 |
| Colombia |  | Deaths | Numb er | Cytomegalo virus | 202  1 | 88.9911639132 2355 | 104.968576934 50232 | 73.0137508919 4478 |
| Comoros |  | Deaths | Numb er | Cytomegalo virus | 202  1 | 2.87681377408 4275 | 3.57734807333 13896 | 2.17627947483 71608 |
| Congo |  | Deaths | Numb er | Cytomegalo virus | 202  1 | 16.9532485958 4776 | 21.0438257918 14832 | 12.8626713998 80687 |
| Cook Islands |  | Deaths | Numb | Cytomegalo | 202 | 0.07094707729 | 0.08352187935 | 0.05837227523 |

Costa Rica

Croatia

Cuba

Cyprus

Czechia

C 么 te d'Ivoire

Democratic People's Republic of Korea

Democratic Republic Congo

of

Denmark

Djibouti

Dominica

Dominican Republic

Ecuador

the

Deaths

Deaths

Deaths

Deaths

Deaths

Deaths

Deaths

Deaths

Deaths

Deaths

Deaths

Deaths

Deaths

er

Numb

er

Numb

er

Numb

er

Numb

er

Numb

er

Numb

er

Numb

er

Numb

er

Numb

er

Numb

er

Numb

er

Numb

er

Numb

virus

Cytomegalo virus

Cytomegalo virus

Cytomegalo virus

Cytomegalo virus

Cytomegalo virus

Cytomegalo virus

Cytomegalo virus

Cytomegalo virus

Cytomegalo virus

Cytomegalo virus

Cytomegalo virus

Cytomegalo virus

Cytomegalo

1

202

1

202

1

202

1

202

1

202

1

202

1

202

1

202

1

202

1

202

1

202

1

202

1

202

76037

8.29618769951 619

9.14199332238 8991

57.5549079890 618

1.66738700155 67536

26.8031027630 56017

86.9890712972 64

78.6795888433 4183

326.944189197 0963

15.0370941091 4745

3.63176225893 13454

0.26351159547 60342

19.2623282240 9549

44.2801487802

8141

9.43906903879 9357

10.5162509761 00237

65.8524303621 4139

1.94665066951 80668

30.6510973110 8684

109.558239605 94208

95.8457482591 4486

414.202401602 81605

17.3529102835 7317

4.74340447509 4572

0.30376740709 36411

24.1939624878 93906

53.1695100442

70665

7.15330636023 3025

7.76773566867 7746

49.2573856159 8221

1.38812333359 54404

22.9551082150 252

64.4199029885 8593

61.5134294275 3881

239.685976791 3766

12.7212779347 2173

2.52012004276 8119

0.22325578385 84273

14.3306939602 97074

35.3907875162

|  |  | er | virus | 1 | 7188 | 865 | 57255 |
| --- | --- | --- | --- | --- | --- | --- | --- |
| Egypt | Deaths | Numb er | Cytomegalo virus | 202  1 | 190.466103138 60705 | 229.051755646 3147 | 151.880450630 89936 |
| El Salvador | Deaths | Numb er | Cytomegalo virus | 202  1 | 17.5287574171 05798 | 21.3190275787 88862 | 13.7384872554 22733 |
| Equatorial Guinea | Deaths | Numb er | Cytomegalo virus | 202  1 | 2.59231318525 37336 | 3.47565933053 2008 | 1.70896703997 54593 |
| Eritrea | Deaths | Numb er | Cytomegalo virus | 202  1 | 31.6966680102 91443 | 46.5693486027 0044 | 16.8239874178 8245 |
| Estonia | Deaths | Numb er | Cytomegalo virus | 202  1 | 2.30942236675 3946 | 2.64750237232 0462 | 1.97134236118 743 |
| Eswatini | Deaths | Numb er | Cytomegalo virus | 202  1 | 4.86392062983 5678 | 6.28412326054 7773 | 3.44371799912 3584 |
| Ethiopia | Deaths | Numb er | Cytomegalo virus | 202  1 | 284.416933895 0785 | 325.305435154 2955 | 243.528432635 86152 |
| Fiji | Deaths | Numb er | Cytomegalo virus | 202  1 | 2.49992836650 458 | 3.09652109640 356 | 1.90333563660 55985 |
| Finland | Deaths | Numb er | Cytomegalo virus | 202  1 | 7.23259490157 1906 | 8.42150027483 3795 | 6.04368952831 0017 |
| France | Deaths | Numb er | Cytomegalo virus | 202  1 | 129.056740829 38356 | 150.664762611 00632 | 107.448719047 76078 |
| Gabon | Deaths | Numb er | Cytomegalo virus | 202  1 | 5.52311867520 5485 | 7.02188419440 2822 | 4.02435315600 8147 |
| Gambia | Deaths | Numb er | Cytomegalo virus | 202  1 | 9.49337714332 5018 | 11.6202728697 711 | 7.36648141687 8937 |
| Georgia | Deaths | Numb | Cytomegalo | 202 | 16.2194790105 | 18.3374088710 | 14.1015491501 |

|  |  | er virus 1 | 5468 | 0465 | 04706 |
| --- | --- | --- | --- | --- | --- |
| Germany | Deaths | Numb Cytomegalo 202  er virus 1 | 157.599001105 82507 | 182.915812908 0724 | 132.282189303 57772 |
| Ghana | Deaths | Numb Cytomegalo 202  er virus 1 | 112.918682221 8557 | 139.141660498 7553 | 86.6957039449 5612 |
| Greece | Deaths | Numb Cytomegalo 202  er virus 1 | 40.6058111586 3111 | 46.8820536938 8584 | 34.3295686233 76375 |
| Greenland | Deaths | Numb Cytomegalo 202  er virus 1 | 0.10774690548 82634 | 0.12676888052 11667 | 0.08872493045 53601 |
| Grenada | Deaths | Numb Cytomegalo 202  er virus 1 | 0.40400504949 0525 | 0.45429693388 90417 | 0.35371316509 20084 |
| Guam | Deaths | Numb Cytomegalo 202  er virus 1 | 0.24221049301 2041 | 0.27869848669 11999 | 0.20572249933 2882 |
| Guatemala | Deaths | Numb Cytomegalo 202  er virus 1 | 53.1133302904 03454 | 61.4558456566 7741 | 44.7708149241 29496 |
| Guinea | Deaths | Numb Cytomegalo 202  er virus 1 | 61.8465119212 3401 | 78.3202259030 8053 | 45.3727979393 87496 |
| Guinea-Bissau | Deaths | Numb Cytomegalo 202  er virus 1 | 8.0462996 | 9.68230319010 1006 | 6.41029601054 8994 |
| Guyana | Deaths | Numb Cytomegalo 202  er virus 1 | 2.63813275248 6144 | 3.29096247852 24127 | 1.98530302644 98737 |
| Haiti | Deaths | Numb Cytomegalo 202  er virus 1 | 45.1898525280 8728 | 57.8402170863 9448 | 32.5394879697 8008 |
| Honduras | Deaths | Numb Cytomegalo 202  er virus 1 | 24.4033558933 04543 | 28.5751531918 12284 | 20.2315585947 968 |
| Hungary | Deaths | Numb Cytomegalo 202 | 18.7098537798 | 21.3631907393 | 16.0565168203 |

|  |  |  |  | er | virus | 1 | 46 | 8625 | 05745 |
| --- | --- | --- | --- | --- | --- | --- | --- | --- | --- |
| Iceland |  |  | Deaths | Numb er | Cytomegalo virus | 202  1 | 0.55807489018 24842 | 0.66467449001 55643 | 0.45147529034 94041 |
| India |  |  | Deaths | Numb er | Cytomegalo virus | 202  1 | 3867.83282449 21977 | 4392.79225720 2161 | 3342.87339178 2234 |
| Indonesia |  |  | Deaths | Numb er | Cytomegalo virus | 202  1 | 601.897247785 0946 | 712.290069770 9154 | 491.504425799 27375 |
| Iran (Islamic | Republic | of) | Deaths | Numb er | Cytomegalo virus | 202  1 | 91.4550202406 6238 | 102.985227354 31877 | 79.9248131270 06 |
| Iraq |  |  | Deaths | Numb er | Cytomegalo virus | 202  1 | 36.0019458209 8692 | 43.9299178970 6606 | 28.0739737449 0777 |
| Ireland |  |  | Deaths | Numb er | Cytomegalo virus | 202  1 | 7.41432091991 5381 | 8.69267497812 4156 | 6.13596686170 6606 |
| Israel |  |  | Deaths | Numb er | Cytomegalo virus | 202  1 | 10.9467948939 84585 | 12.7256016725 47652 | 9.16798811542 1515 |
| Italy |  |  | Deaths | Numb er | Cytomegalo virus | 202  1 | 106.239088504 6508 | 124.171408120 57548 | 88.3067688887 261 |
| Jamaica |  |  | Deaths | Numb er | Cytomegalo virus | 202  1 | 6.33686721769 7929 | 7.78510381398 8464 | 4.88863062140 7393 |
| Japan |  |  | Deaths | Numb er | Cytomegalo virus | 202  1 | 462.081128646 9067 | 548.259826783 4851 | 375.902430510 3282 |
| Jordan |  |  | Deaths | Numb er | Cytomegalo virus | 202  1 | 7.92222533533 8368 | 9.58438501140 3327 | 6.26006565927 3408 |
| Kazakhstan |  |  | Deaths | Numb er | Cytomegalo virus | 202  1 | 36.1096392420 9477 | 40.7126007541 1998 | 31.5066777300 6957 |
| Kenya |  |  | Deaths | Numb | Cytomegalo | 202 | 162.400535154 | 194.674164927 | 130.126905381 |

Kiribati

Kuwait

Kyrgyzstan

Lao People's Republic

Latvia

Lebanon

Lesotho

Liberia

Libya

Lithuania

Luxembourg

Madagascar

Malawi

Deaths Deaths

Deaths

Democratic

Deaths

Deaths Deaths

Deaths Deaths

Deaths Deaths

Deaths

Deaths Deaths

er

Numb

er

Numb

er

Numb

er

Numb

er

Numb

er

Numb

er

Numb

er

Numb

er

Numb

er

Numb

er

Numb

er

Numb

er

Numb

virus

Cytomegalo virus

Cytomegalo virus

Cytomegalo virus

Cytomegalo virus

Cytomegalo virus

Cytomegalo virus

Cytomegalo virus

Cytomegalo virus

Cytomegalo virus

Cytomegalo virus

Cytomegalo virus

Cytomegalo virus

Cytomegalo

1

202

1

202

1

202

1

202

1

202

1

202

1

202

1

202

1

202

1

202

1

202

1

202

1

202

68338

0.32882241396 64588

3.21232840352 78457

6.52135349093 9086

22.0876533684 61403

4.67480993974 5549

14.9480842593 66144

11.8763363006 45772

13.8866719673 93529

9.22436086049 8168

5.88051546571 0504

0.82918123720 68321

106.782266170 1598

66.1870799819

92483

0.40106028418 76349

3.87037651568 66727

7.56443392085 1152

27.1792774000 6368

5.38293873154 4055

17.5419010733 5199

14.6868268155 44174

17.8931979931 97923

11.4658108112 9728

6.83181190135 9457

0.94946481089 72495

131.391839451 99767

79.8342246046

44193

0.25658454374 52827

2.55428029136 90186

5.47827306102 70205

16.9960293368 59127

3.96668114794 7043

12.3542674453 80298

9.06584578574 7372

9.88014594158 9134

6.98291090969 9055

4.92921903006 155

0.70889766351 64147

82.1726928883 219

52.5399353592

|  |  | er | virus | 1 | 5018 | 3137 | 6899 |
| --- | --- | --- | --- | --- | --- | --- | --- |
| Malaysia | Deaths | Numb er | Cytomegalo virus | 202  1 | 147.518671356 1257 | 168.549266770 15985 | 126.488075942 09156 |
| Maldives | Deaths | Numb er | Cytomegalo virus | 202  1 | 0.41874520975 77229 | 0.50089578241 84118 | 0.33659463709 70341 |
| Mali | Deaths | Numb er | Cytomegalo virus | 202  1 | 67.8761199022 6293 | 81.6697581750 7802 | 54.0824816294 4784 |
| Malta | Deaths | Numb er | Cytomegalo virus | 202  1 | 1.05931461613 7361 | 1.24647598587 7678 | 0.87215324639 70441 |
| Marshall Islands | Deaths | Numb er | Cytomegalo virus | 202  1 | 0.17987446743 31827 | 0.22305529993 72501 | 0.13669363492 91153 |
| Mauritania | Deaths | Numb er | Cytomegalo virus | 202  1 | 12.9036512555 61057 | 15.9140157502 48744 | 9.89328676087 3366 |
| Mauritius | Deaths | Numb er | Cytomegalo virus | 202  1 | 3.51377328272 621 | 3.87305122888 8296 | 3.15449533656 4124 |
| Mexico | Deaths | Numb er | Cytomegalo virus | 202  1 | 315.952960788 9684 | 357.460349705 3352 | 274.445571872 60153 |
| Micronesia (Federated States of) | Deaths | Numb er | Cytomegalo virus | 202  1 | 0.35264109612 31177 | 0.43838608375 61486 | 0.26689610849 00868 |
| Monaco | Deaths | Numb er | Cytomegalo virus | 202  1 | 0.11382348517 23901 | 0.13926742645 13688 | 0.08837954389 34115 |
| Mongolia | Deaths | Numb er | Cytomegalo virus | 202  1 | 4.41985348505 7267 | 5.05614262418 6906 | 3.78356434592 7628 |
| Montenegro | Deaths | Numb er | Cytomegalo virus | 202  1 | 1.15404815101 05254 | 1.34286018167 80994 | 0.96523612034 29514 |
| Morocco | Deaths | Numb | Cytomegalo | 202 | 82.3543756021 | 98.6012348877 | 66.1075163165 |

|  |  |  | er | virus | 1 | 9316 | 9352 | 9281 |
| --- | --- | --- | --- | --- | --- | --- | --- | --- |
| Mozambique |  | Deaths | Numb er | Cytomegalo virus | 202  1 | 100.234340554 34448 | 123.319486457 68368 | 77.1491946510 053 |
| Myanmar |  | Deaths | Numb er | Cytomegalo virus | 202  1 | 196.664851053 2929 | 236.039369993 47712 | 157.290332113 10866 |
| Namibia |  | Deaths | Numb er | Cytomegalo virus | 202  1 | 9.00086785233 4133 | 11.7829069497 53205 | 6.21882875491 5064 |
| Nauru |  | Deaths | Numb er | Cytomegalo virus | 202  1 | 0.03506273247 81473 | 0.04369362162 17652 | 0.02643184333 45293 |
| Nepal |  | Deaths | Numb er | Cytomegalo virus | 202  1 | 82.1032949812 9278 | 100.570592011 69218 | 63.6359979508 9336 |
| Netherlands |  | Deaths | Numb er | Cytomegalo virus | 202  1 | 36.6676507268 28725 | 42.1546757121 05046 | 31.1806257415 52404 |
| New Zealand |  | Deaths | Numb er | Cytomegalo virus | 202  1 | 7.02438179644 5185 | 8.07960633194 4504 | 5.96915726094 5867 |
| Nicaragua |  | Deaths | Numb er | Cytomegalo virus | 202  1 | 8.16474192663 6975 | 9.598567974 | 6.73091587944 8949 |
| Niger |  | Deaths | Numb er | Cytomegalo virus | 202  1 | 93.9508711293 2804 | 118.598901962 51104 | 69.3028402961 4504 |
| Nigeria |  | Deaths | Numb er | Cytomegalo virus | 202  1 | 670.029274235 3026 | 821.139155974 4704 | 518.919392496 1348 |
| Niue |  | Deaths | Numb er | Cytomegalo virus | 202  1 | 0.00897082650 58737 | 0.01035634557 62314 | 0.00758530743 5516 |
| North Macedonia |  | Deaths | Numb er | Cytomegalo virus | 202  1 | 4.19401769742 1444 | 5.06392425597 2175 | 3.32411113887 07125 |
| Northern Mariana | Islands | Deaths | Numb | Cytomegalo | 202 | 0.10200934767 | 0.11370787792 | 0.09031081742 |

|  |  | er | virus | 1 | 42345 | 69769 | 14922 |
| --- | --- | --- | --- | --- | --- | --- | --- |
| Norway | Deaths | Numb er | Cytomegalo virus | 202  1 | 10.3721341784 67109 | 12.0910441347 39526 | 8.65322422219 4687 |
| Oman | Deaths | Numb er | Cytomegalo virus | 202  1 | 2.94968786450 7802 | 3.48988063633 35764 | 2.40949509268 2029 |
| Pakistan | Deaths | Numb er | Cytomegalo virus | 202  1 | 436.648754669 17175 | 513.419754740 9055 | 359.877754597 438 |
| Palau | Deaths | Numb er | Cytomegalo virus | 202  1 | 0.06472690061 7875 | 0.07727166146 06492 | 0.05218213977 51009 |
| Palestine | Deaths | Numb er | Cytomegalo virus | 202  1 | 5.23333828589 019 | 5.95469874861 2347 | 4.51197782316 8032 |
| Panama | Deaths | Numb er | Cytomegalo virus | 202  1 | 6.72130058329 6619 | 8.12016342820 5613 | 5.32243773838 7626 |
| Papua New Guinea | Deaths | Numb er | Cytomegalo virus | 202  1 | 30.3036589365 1576 | 37.0113068304 8488 | 23.5960110425 4663 |
| Paraguay | Deaths | Numb er | Cytomegalo virus | 202  1 | 14.5539254771 49598 | 17.9245259893 26046 | 11.1833249649 73147 |
| Peru | Deaths | Numb er | Cytomegalo virus | 202  1 | 144.831561282 174 | 179.990093959 65774 | 109.673028604 69024 |
| Philippines | Deaths | Numb er | Cytomegalo virus | 202  1 | 415.846247680 1777 | 489.513025477 7649 | 342.179469882 5905 |
| Poland | Deaths | Numb er | Cytomegalo virus | 202  1 | 124.974318868 97952 | 140.157577828 7287 | 109.791059909 23031 |
| Portugal | Deaths | Numb er | Cytomegalo virus | 202  1 | 42.2893657554 3924 | 48.6818268574 8032 | 35.8969046533 9816 |
| Puerto Rico | Deaths | Numb | Cytomegalo | 202 | 9.71344240209 | 11.6126938770 | 7.81419092718 |

|  |  | er | virus | 1 | 8034 | 0928 | 6788 |
| --- | --- | --- | --- | --- | --- | --- | --- |
| Qatar | Deaths | Numb er | Cytomegalo virus | 202  1 | 0.64410857731 80272 | 0.80483063907 67005 | 0.48338651555 93538 |
| Republic of Korea | Deaths | Numb er | Cytomegalo virus | 202  1 | 86.2197667452 9586 | 102.702909608 30016 | 69.7366238822 9154 |
| Republic of Moldova | Deaths | Numb er | Cytomegalo virus | 202  1 | 8.38058679853 1601 | 9.41661555244 2128 | 7.34455804462 1074 |
| Romania | Deaths | Numb er | Cytomegalo virus | 202  1 | 63.4082422918 10666 | 71.5606505798 4297 | 55.2558340037 7837 |
| Russian Federation | Deaths | Numb er | Cytomegalo virus | 202  1 | 239.189958764 5031 | 272.147457886 3515 | 206.232459642 6547 |
| Rwanda | Deaths | Numb er | Cytomegalo virus | 202  1 | 39.6548888137 2602 | 50.4055294886 94725 | 28.9042481387 5731 |
| Saint Kitts and Nevis | Deaths | Numb er | Cytomegalo virus | 202  1 | 0.19679731911 15597 | 0.22531996844 49371 | 0.16827466977 81823 |
| Saint Lucia | Deaths | Numb er | Cytomegalo virus | 202  1 | 0.65935533796 31301 | 0.78246307480 68945 | 0.53624760111 93656 |
| Saint Vincent and the Grenadines | Deaths | Numb er | Cytomegalo virus | 202  1 | 0.47246169281 20971 | 0.53391954574 96146 | 0.41100383987 45796 |
| Samoa | Deaths | Numb er | Cytomegalo virus | 202  1 | 0.58926905921 52066 | 0.69616906633 5482 | 0.48236905209 49311 |
| San Marino | Deaths | Numb er | Cytomegalo virus | 202  1 | 0.03728599300 53081 | 0.05044212821 20024 | 0.02412985779 86138 |
| Sao Tome and Principe | Deaths | Numb er | Cytomegalo virus | 202  1 | 0.77708452449 48195 | 0.90279996237 65566 | 0.65136908661 30823 |
| Saudi Arabia | Deaths | Numb | Cytomegalo | 202 | 34.3857809801 | 42.2504533130 | 26.5211086472 |

|  |  | er | virus | 1 | 3116 | 1192 | 504 |
| --- | --- | --- | --- | --- | --- | --- | --- |
| Senegal | Deaths | Numb er | Cytomegalo virus | 202  1 | 50.7237929196 6274 | 61.2858934169 0423 | 40.1616924224 2125 |
| Serbia | Deaths | Numb er | Cytomegalo virus | 202  1 | 23.4351528362 0944 | 27.5505333455 84213 | 19.3197723268 3465 |
| Seychelles | Deaths | Numb er | Cytomegalo virus | 202  1 | 0.44836341563 88529 | 0.51551160282 87698 | 0.38121522844 8936 |
| Sierra Leone | Deaths | Numb er | Cytomegalo virus | 202  1 | 32.5167016095 6882 | 41.2503647441 1024 | 23.7830384750 27404 |
| Singapore | Deaths | Numb er | Cytomegalo virus | 202  1 | 15.1255568454 67024 | 17.4026101007 8319 | 12.8485035901 5086 |
| Slovakia | Deaths | Numb er | Cytomegalo virus | 202  1 | 12.6674396099 45353 | 14.6765658373 34032 | 10.6583133825 56669 |
| Slovenia | Deaths | Numb er | Cytomegalo virus | 202  1 | 3.60173854432 6027 | 4.19643679556 0807 | 3.00704029309 1247 |
| Solomon Islands | Deaths | Numb er | Cytomegalo virus | 202  1 | 3.40877595559 6832 | 4.21212262659 4471 | 2.60542928459 9193 |
| Somalia | Deaths | Numb er | Cytomegalo virus | 202  1 | 77.9045136609 3187 | 97.4137599338 1112 | 58.3952673880 5263 |
| South Africa | Deaths | Numb er | Cytomegalo virus | 202  1 | 222.738701760 0049 | 246.412395826 08023 | 199.065007693 92952 |
| South Sudan | Deaths | Numb er | Cytomegalo virus | 202  1 | 38.5837769036 8056 | 50.0541273692 6486 | 27.1134264380 9625 |
| Spain | Deaths | Numb er | Cytomegalo virus | 202  1 | 90.4714353994 5114 | 105.742170065 38372 | 75.2007007335 1854 |
| Sri Lanka | Deaths | Numb | Cytomegalo | 202 | 38.2992571789 | 50.4596092497 | 26.1389051081 |

|  |  | er | virus | 1 | 64496 | 88656 | 4034 |
| --- | --- | --- | --- | --- | --- | --- | --- |
| Sudan | Deaths | Numb er | Cytomegalo virus | 202  1 | 54.7795176866 9534 | 69.7325620383 0323 | 39.8264733350 8746 |
| Suriname | Deaths | Numb er | Cytomegalo virus | 202  1 | 1.62039873973 59063 | 2.11232122251 1059 | 1.12847625696 07534 |
| Sweden | Deaths | Numb er | Cytomegalo virus | 202  1 | 15.6982982787 30611 | 18.3128455657 0575 | 13.0837509917 55474 |
| Switzerland | Deaths | Numb er | Cytomegalo virus | 202  1 | 11.6706773740 68577 | 13.7122062084 06728 | 9.62914853973 0426 |
| Syrian Arab Republic | Deaths | Numb er | Cytomegalo virus | 202  1 | 22.7780466912 6038 | 28.6393255099 1811 | 16.9167678726 02648 |
| Taiwan (Province of China) | Deaths | Numb er | Cytomegalo virus | 202  1 | 75.1628774391 4425 | 85.8826422483 168 | 64.4431126299 7171 |
| Tajikistan | Deaths | Numb er | Cytomegalo virus | 202  1 | 18.9966781380 4324 | 23.2329503517 65915 | 14.7604059243 20564 |
| Thailand | Deaths | Numb er | Cytomegalo virus | 202  1 | 256.419967361 1379 | 323.432144055 9429 | 189.407790666 3329 |
| Timor-Leste | Deaths | Numb er | Cytomegalo virus | 202  1 | 4.19806919124 936 | 5.11853844106 0809 | 3.27759994143 791 |
| Togo | Deaths | Numb er | Cytomegalo virus | 202  1 | 31.8297908511 13767 | 40.1071320585 3331 | 23.5524496436 94228 |
| Tokelau | Deaths | Numb er | Cytomegalo virus | 202  1 | 0.00533718719 12421 | 0.00634813117 82412 | 0.00432624320 4243 |
| Tonga | Deaths | Numb er | Cytomegalo virus | 202  1 | 0.38030749073 02075 | 0.46708551315 23808 | 0.29352946830 80341 |
| Trinidad and Tobago | Deaths | Numb | Cytomegalo | 202 | 3.82389247972 | 4.75397069113 | 2.89381426831 |

|  |  | er | virus | 1 | 69993 | 4568 | 94306 |
| --- | --- | --- | --- | --- | --- | --- | --- |
| Tunisia | Deaths | Numb er | Cytomegalo virus | 202  1 | 17.1827187093 2076 | 22.1405299804 5948 | 12.2249074381 82049 |
| Turkmenistan | Deaths | Numb er | Cytomegalo virus | 202  1 | 8.20414536687 8096 | 9.79652682571 6056 | 6.61176390804 0137 |
| Tuvalu | Deaths | Numb er | Cytomegalo virus | 202  1 | 0.04377269675 19467 | 0.05077531340 91756 | 0.03677008009 47178 |
| T 眉 rkiye | Deaths | Numb er | Cytomegalo virus | 202  1 | 147.107970626 24715 | 175.016763202 9504 | 119.199178049 5439 |
| Uganda | Deaths | Numb er | Cytomegalo virus | 202  1 | 105.267833489 44474 | 126.748968618 49103 | 83.7866983603 9845 |
| Ukraine | Deaths | Numb er | Cytomegalo virus | 202  1 | 73.7457109458 1336 | 94.8610730812 0305 | 52.6303488104 2368 |
| United Arab Emirates | Deaths | Numb er | Cytomegalo virus | 202  1 | 4.05271247173 0338 | 4.88133870065 9487 | 3.22408624280 1189 |
| United Kingdom | Deaths | Numb er | Cytomegalo virus | 202  1 | 203.529245827 0243 | 232.838785137 158 | 174.219706516 89062 |
| United Republic of Tanzania | Deaths | Numb er | Cytomegalo virus | 202  1 | 180.189034456 7828 | 217.176142273 67264 | 143.201926639 89295 |
| United States of America | Deaths | Numb er | Cytomegalo virus | 202  1 | 589.101112110 1388 | 666.601315519 9232 | 511.600908700 3544 |
| United States Virgin Islands | Deaths | Numb er | Cytomegalo virus | 202  1 | 0.20806058523 59136 | 0.25450587806 11151 | 0.16161529241 07121 |
| Uruguay | Deaths | Numb er | Cytomegalo virus | 202  1 | 14.0764085690 24352 | 15.7855073560 7823 | 12.3673097819 70471 |
| Uzbekistan | Deaths | Numb | Cytomegalo | 202 | 50.1403032428 | 57.4425454434 | 42.8380610421 |

Vanuatu Deaths

Venezuela (Bolivarian

Deaths

Republic of)

| Viet Nam  Yemen  Zambia  Zimbabwe | Deaths  Deaths  Deaths  Deaths |
| --- | --- |

er

Numb

er

Numb

er

Numb

er

Numb

er

Numb

er

Numb

er

virus

Cytomegalo virus

Cytomegalo virus

Cytomegalo virus

Cytomegalo virus

Cytomegalo virus

Cytomegalo virus

1

202

1

202

1

202

1

202

1

202

1

202

1

3165

0.77882176965 67923

63.8936167702 98365

199.683679676 3394

60.0541608704 8563

58.0114079037 5702

81.9148171097 3147

92975

0.89518262238 85347

80.2738243085 1414

238.986413686 62032

75.1383562547 9747

72.3887591120 4684

100.759658452 94472

7032

0.66246091692 50498

47.5134092320 82594

160.380945666 0585

44.9699654861 7379

43.6340566954 672

63.0699757665 1823

Table S2 Age-standardized DALYs and deaths rate and SDI across all regions between1990 and 2021.

| measure_name | location_name | | age_name | cause_name | metric_n ame | year | val | upper | lower |
| --- | --- | --- | --- | --- | --- | --- | --- | --- | --- |
| DALYs (Disability-Adjusted Life Years) | Andean Latin America | | Age-standar dized | Cytomegalov irus | Rate | 1990 | 18.94608  275 | 21.47585  681 | 16.416308  7 |
| DALYs (Disability-Adjusted Life Years) | Andean Latin America | | Age-standar dized | Cytomegalov irus | Rate | 2021 | 8.493272  032 | 10.17090  624 | 6.8156378  21 |
| DALYs (Disability-Adjusted Life Years) | Australasia | | Age-standar dized | Cytomegalov irus | Rate | 1990 | 2.138036  893 | 2.370684  448 | 1.9053893  38 |
| DALYs (Disability-Adjusted Life Years) | Australasia | | Age-standar dized | Cytomegalov irus | Rate | 2021 | 1.056568  024 | 1.170665  194 | 0.9424708  55 |
| DALYs (Disability-Adjusted Life Years) | Caribbean | | Age-standar dized | Cytomegalov irus | Rate | 1990 | 12.16806  544 | 14.09095  744 | 10.245173  44 |
| DALYs (Disability-Adjusted Life Years) | Caribbean | | Age-standar dized | Cytomegalov irus | Rate | 2021 | 8.166427  44 | 9.733001  484 | 6.5998533  95 |
| DALYs (Disability-Adjusted Life Years) | Central | Asia | Age-standar dized | Cytomegalov irus | Rate | 1990 | 16.26166  6 | 18.89498  345 | 13.628348  54 |
| DALYs (Disability-Adjusted Life Years) | Central | Asia | Age-standar dized | Cytomegalov irus | Rate | 2021 | 7.489976  533 | 8.610889  069 | 6.3690639  97 |
| DALYs (Disability-Adjusted Life Years) | Central | Europe | Age-standar dized | Cytomegalov irus | Rate | 1990 | 5.207249  048 | 5.657702  004 | 4.7567960  92 |
| DALYs (Disability-Adjusted Life Years) | Central | Europe | Age-standar dized | Cytomegalov irus | Rate | 2021 | 3.034820  861 | 3.312566  103 | 2.7570756  19 |
| DALYs (Disability-Adjusted Life Years) | Central | Latin America | Age-standar dized | Cytomegalov irus | Rate | 1990 | 9.442643  905 | 10.23994  179 | 8.6453460  2 |
| DALYs (Disability-Adjusted Life Years) | Central | Latin America | Age-standar dized | Cytomegalov irus | Rate | 2021 | 5.817929  962 | 6.550919  527 | 5.0849403  96 |

DALYs (Disability-Adjusted Life Years)

DALYs (Disability-Adjusted Life Years)

DALYs (Disability-Adjusted Life Years)

DALYs (Disability-Adjusted Life Years)

DALYs (Disability-Adjusted Life Years)

DALYs (Disability-Adjusted Life Years)

DALYs (Disability-Adjusted Life Years)

DALYs (Disability-Adjusted Life Years)

DALYs (Disability-Adjusted Life Years)

DALYs (Disability-Adjusted Life Years)

DALYs (Disability-Adjusted Life Years)

DALYs (Disability-Adjusted Life Years)

DALYs (Disability-Adjusted Life Years)

Central Sub-Saharan Africa

Central Sub-Saharan Africa

East Asia

East Asia

Eastern Europe

Eastern Europe

Eastern Sub-Saharan Africa

Eastern Sub-Saharan Africa

Global

| Global  High-income Pacific  High-income Pacific  High-income America | Asia  Asia  North |
| --- | --- |

Age-standar dized

Age-standar dized

Age-standar dized

Age-standar dized

Age-standar dized

Age-standar dized

Age-standar dized

Age-standar dized

Age-standar dized

Age-standar dized

Age-standar dized

Age-standar dized

Age-standar dized

Cytomegalov

Rate irus

Cytomegalov

Rate irus

Cytomegalov

Rate irus

Cytomegalov

Rate irus

Cytomegalov

Rate irus

Cytomegalov

Rate irus

Cytomegalov

Rate irus

Cytomegalov

Rate irus

Cytomegalov

Rate irus

Cytomegalov

Rate irus

Cytomegalov

Rate irus

Cytomegalov

Rate irus

Cytomegalov

Rate irus

33.97816 1990

459

22.12057 2021

345

13.43673 1990

336

2 846977

2021 .

933

3 890679

1990 .

178

2.732411 2021

254

34.24124 1990

4

18.91732 2021

58

13.89441 1990

745

6 951881

2021 .

079

4 293986

1990 .

126

1.465152 2021

407

2.869074 1990

548

41.14370 615

27.08826 268

15.21041 478

3.365361 672

4.307640 339

3.024400 184

39.36042 396

21.58652 237

15.93279 293

7.828026 004

4.750634 154

1.634768 85

3.169853 341

26.812623 03

17.152884 23

11.663051 95

2.3285941 94

3.4737180 18

2.4404223 25

29.122064 03

16.248129 23

11.856041 96

6.0757361 53

3.8373380 98

1.2955359 65

2.5682957 54

DALYs (Disability-Adjusted Life Years)

DALYs (Disability-Adjusted Life Years)

DALYs (Disability-Adjusted Life Years)

DALYs (Disability-Adjusted Life Years)

DALYs (Disability-Adjusted Life Years)

DALYs (Disability-Adjusted Life Years)

DALYs (Disability-Adjusted Life Years)

DALYs (Disability-Adjusted Life Years)

DALYs (Disability-Adjusted Life Years)

DALYs (Disability-Adjusted Life Years)

DALYs (Disability-Adjusted Life Years)

DALYs (Disability-Adjusted Life Years)

DALYs (Disability-Adjusted Life Years)

High-income North America

North Africa and Middle East

North Africa and Middle East

Oceania

Oceania

South Asia

South Asia

Southeast Asia

Southeast Asia

Southern Latin America

Southern Latin America

Southern Sub-Saharan Africa

Southern Sub-Saharan Africa

Age-standar dized

Age-standar dized

Age-standar dized

Age-standar dized

Age-standar dized

Age-standar dized

Age-standar dized

Age-standar dized

Age-standar dized

Age-standar dized

Age-standar dized

Age-standar dized

Age-standar dized

Cytomegalov

Rate irus

Cytomegalov

Rate irus

Cytomegalov

Rate irus

Cytomegalov

Rate irus

Cytomegalov

Rate irus

Cytomegalov

Rate irus

Cytomegalov

Rate irus

Cytomegalov

Rate irus

Cytomegalov

Rate irus

Cytomegalov

Rate irus

Cytomegalov

Rate irus

Cytomegalov

Rate irus

Cytomegalov

Rate irus

2.000689 2021

498

13.34438 1990

471

5.735251 2021

976

21 10045

1990 .

423

16 11534

2021 .

724

19.70156 1990

834

9.764374 2021

467

14.86476 1990

266

8.116952 2021

548

6 119225

1990 .

588

5 932993

2021 .

619

16.71508 1990

451

16.87769 2021

122

2.177734 03

15.34161 8

6.542681 201

25.30281 753

19.30741 026

23.13017 657

11.07446 976

16.91515 951

9.108469 747

6.609637 571

6.487467 06

18.73505 854

19.07834 4

1.8236449 66

11.347151 43

4.9278227 51

16.898090 94

12.923284 22

16.272960 11

8.4542791 78

12.814365 82

7.1254353 5

5.6288136 06

5.3785201 79

14.695110 49

14.677038 43

| DALYs (Disability-Adjusted Life Years) | Tropical Latin America | Age-standar dized | Cytomegalov irus | Rate | 1990 | 9.286008  883 | 10.13829  914 | 8.4337186  21 |
| --- | --- | --- | --- | --- | --- | --- | --- | --- |
| DALYs (Disability-Adjusted Life Years) | Tropical Latin America | Age-standar dized | Cytomegalov irus | Rate | 2021 | 6.555474  868 | 7.131648  681 | 5.9793010  55 |
| DALYs (Disability-Adjusted Life Years) | Western Europe | Age-standar dized | Cytomegalov irus | Rate | 1990 | 2.689479  553 | 2.971980  837 | 2.4069782  7 |
| DALYs (Disability-Adjusted Life Years) | Western Europe | Age-standar dized | Cytomegalov irus | Rate | 2021 | 1.388283  145 | 1.528209  995 | 1.2483562  95 |
| DALYs (Disability-Adjusted Life Years) | Western Sub-Saharan Africa | Age-standar dized | Cytomegalov irus | Rate | 1990 | 29.00687  884 | 33.83120  735 | 24.182550  33 |
| DALYs (Disability-Adjusted Life Years) | Western Sub-Saharan Africa | Age-standar dized | Cytomegalov irus | Rate | 2021 | 18.55849  446 | 22.12296  932 | 14.994019  6 |
| measure_name | location_name | age_name | cause_name | metric_n ame | year | val | upper | lower |
| Deaths | Andean Latin America | Age-standar dized | Cytomegalov irus | Rate | 1990 | 0.699916  718 | 0.786953  106 | 0.6128803  29 |
| Deaths | Andean Latin America | Age-standar dized | Cytomegalov irus | Rate | 2021 | 0.416655  412 | 0.497985  217 | 0.3353256  08 |
| Deaths | Australasia | Age-standar dized | Cytomegalov irus | Rate | 1990 | 0.120402  96 | 0.135692  487 | 0.1051134  33 |
| Deaths | Australasia | Age-standar dized | Cytomegalov irus | Rate | 2021 | 0.060283  297 | 0.069004  503 | 0.0515620  9 |
| Deaths | Caribbean | Age-standar dized | Cytomegalov irus | Rate | 1990 | 0.402124  241 | 0.447331  006 | 0.3569174  76 |
| Deaths | Caribbean | Age-standar dized | Cytomegalov irus | Rate | 2021 | 0.297076  521 | 0.342239  803 | 0.2519132  4 |

Deaths

Deaths

Deaths

Deaths

Deaths

Deaths

Deaths

Deaths

Deaths

Deaths

Deaths

Deaths

Deaths

Central Asia

Central Asia

Central Europe

Central Europe

Central Latin America

Central Latin America

Central Sub-Saharan Africa

Central Sub-Saharan Africa

East Asia

East Asia

Eastern Europe

Eastern Europe

Eastern Sub-Saharan Africa

Age-standar dized

Age-standar dized

Age-standar dized

Age-standar dized

Age-standar dized

Age-standar dized

Age-standar dized

Age-standar dized

Age-standar dized

Age-standar dized

Age-standar dized

Age-standar dized

Age-standar dized

Cytomegalov

Rate irus

Cytomegalov

Rate irus

Cytomegalov

Rate irus

Cytomegalov

Rate irus

Cytomegalov

Rate irus

Cytomegalov

Rate irus

Cytomegalov

Rate irus

Cytomegalov

Rate irus

Cytomegalov

Rate irus

Cytomegalov

Rate irus

Cytomegalov

Rate irus

Cytomegalov

Rate irus

Cytomegalov

Rate irus

0.304317 1990

112

0.220662 2021

4

0.222747 1990

283

0 143508

2021 .

824

0 380671

1990 .

322

0.245935 2021

612

1.164520 1990

854

0.971491 2021

1

0.519029 1990

48

0 153730

2021 .

485

0 137468

1990 .

673

0.104530 2021

615

1.142972 1990

506

0.341462 251

0.247833 047

0.246147 11

0.158922 425

0.414209 535

0.275753 894

1.431086 344

1.204332 093

0.591568 351

0.185028 824

0.155958 387

0.117781 296

1.306472 529

0.2671719 73

0.1934917 53

0.1993474 55

0.1280952 23

0.3471331 09

0.2161173 3

0.8979553 64

0.7386501 07

0.4464906 08

0.1224321 46

0.1189789 58

0.0912799 34

0.9794724 82

Deaths

Deaths

Deaths

Deaths

Deaths

Deaths

Deaths

Deaths

Deaths

Deaths

Deaths

Deaths

Deaths

Eastern Sub-Saharan Africa

Global

Global

High-income Asia Pacific

High-income Asia Pacific

High-income North America

High-income North America

North Africa and Middle East

North Africa and Middle East

Oceania

Oceania

South Asia

South Asia

Age-standar dized

Age-standar dized

Age-standar dized

Age-standar dized

Age-standar dized

Age-standar dized

Age-standar dized

Age-standar dized

Age-standar dized

Age-standar dized

Age-standar dized

Age-standar dized

Age-standar dized

Cytomegalov

Rate irus

Cytomegalov

Rate irus

Cytomegalov

Rate irus

Cytomegalov

Rate irus

Cytomegalov

Rate irus

Cytomegalov

Rate irus

Cytomegalov

Rate irus

Cytomegalov

Rate irus

Cytomegalov

Rate irus

Cytomegalov

Rate irus

Cytomegalov

Rate irus

Cytomegalov

Rate irus

Cytomegalov

Rate irus

0.790603 2021

43

0.396862 1990

327

0.239994 2021

492

0 252128

1990 .

793

0 088476

2021 .

344

0.152177 1990

613

0.094742 2021

798

0.409773 1990

713

0.240461 2021

159

0 667263

1990 .

688

0 540591

2021 .

553

0.550397 1990

7

0.376947 2021

842

0.896724 707

0.440657 09

0.265987 985

0.284219 493

0.102161 018

0.171658 786

0.106128 551

0.456967 493

0.272614 343

0.789249 639

0.634961 057

0.631691 425

0.425571 202

0.6844821 53

0.3530675 63

0.2140009 99

0.2200380 93

0.0747916 71

0.1326964 41

0.0833570 46

0.3625799 32

0.2083079 74

0.5452777 38

0.4462220 49

0.4691039 75

0.3283244 82

Deaths

Deaths

Deaths

Deaths

Deaths

Deaths

Deaths

Deaths

Deaths

Deaths

Deaths

Deaths

Southeast Asia

Southeast Asia

| Southern  America  Southern  America  Southern Africa  Southern Africa  Tropical  America  Tropical | Latin  Latin  Sub-Saharan  Sub-Saharan  Latin  Latin |
| --- | --- |

America

Western Europe

Western Europe

Western Sub-Saharan Africa

Western Sub-Saharan Africa

Age-standar dized

Age-standar dized

Age-standar dized

Age-standar dized

Age-standar dized

Age-standar dized

Age-standar dized

Age-standar dized

Age-standar dized

Age-standar dized

Age-standar dized

Age-standar dized

Cytomegalov

Rate irus

Cytomegalov

Rate irus

Cytomegalov

Rate irus

Cytomegalov

Rate irus

Cytomegalov

Rate irus

Cytomegalov

Rate irus

Cytomegalov

Rate irus

Cytomegalov

Rate irus

Cytomegalov

Rate irus

Cytomegalov

Rate irus

Cytomegalov

Rate irus

Cytomegalov

Rate irus

0.469877 1990

027

0.369688 2021

053

0.312964 1990

704

0 325324

2021 .

309

0 582623

1990 .

787

0.639207 2021

778

0.387104 1990

317

0.320011 2021

832

0.162174 1990

763

0 080309

2021 .

762

0 857036

1990 .

776

0.694553 2021

675

0.533207 445

0.417935 69

0.342922 954

0.362774 322

0.657129 321

0.717472 941

0.428082 562

0.358172 934

0.182376 106

0.091369 46

0.977117 976

0.807988 757

0.4065466 08

0.3214404 16

0.2830064 53

0.2878742 96

0.5081182 53

0.5609426 15

0.3461260 71

0.2818507 3

0.1419734 21

0.0692500 64

0.7369555 75

0.5811185 94

Table S3 Age, Period, and Cohort Effects on global LRI caused by CMV Mortality: Relative Risk Analysis.

age val low up type sex_name location_name

1 2.5 96.62571581851401 84.42379261910675 110.5912050121187 Age effect Both Global

2 7.5 5.468991925566054 4.750605093263017 6.296013264567672 Age effect Both Global

3 12.5 2.39018517611065 2.063923342213537 2.768021979911319 Age effect Both Global

4 17.5 2.640881300112369 2.305736423610063 3.024740369223858 Age effect Both Global

5 22.5 1.829764225601937 1.595790851286764 2.098042559018916 Age effect Both Global

6 27.5 1.566460967229714 1.370218434212542 1.790809334180677 Age effect Both Global

7 32.5 1.622247140604736 1.431793553032463 1.838034386749731 Age effect Both Global

8 37.5 1.920158842648025 1.721365640123255 2.141909827324893 Age effect Both Global

9 42.5 2.399521524271917 2.176349198982014 2.645578911756158 Age effect Both Global

10 47.5 3.120124475056423 2.8546388481186 3.410300657213524 Age effect Both Global

11 52.5 6.561172721420762 6.105367254863229 7.051007037459584 Age effect Both Global

12 57.5 8.62558254272341 8.054220000530748 9.23747727234072 Age effect Both Global

13 62.5 12.06108716494701 11.2889531649274 12.88603305153186 Age effect Both Global

14 67.5 15.44829459089583 14.31228428368784 16.67447355270172 Age effect Both Global

15 72.5 20.57792570389829 19.02188358751345 22.26125632232995 Age effect Both Global

16 77.5 25.41625279816247 23.40910339283549 27.5954997275893 Age effect Both Global

17 82.5 31.24724936470119 28.62001596554676 34.11565507284181 Age effect Both Global

18 87.5 38.58442677782487 34.98302755527705 42.5565793989936 Age effect Both Global

19 92.5 49.5643562266846 43.9888955081193 55.84648988770937 Age effect Both Global

20 97.5 61.94262511659139 52.30219273665534 73.35999898997623 Age effect Both Global

21 1897 1.880680060626007 1.137002393148034 3.110774006942431 Cohort RR Both Global

22 1902 1.868910485543021 1.466212258945741 2.382210612182521 Cohort RR Both Global

23 1907 1.822209062680235 1.56132183011343 2.126688940148073 Cohort RR Both Global

24 1912 1.748084422461125 1.551729456295176 1.969286034787987 Cohort RR Both Global

| 25 | 1917 | 1.739102098307875 | 1.569076961333331 | 1.927551154513664 | Cohort | RR | Both | Global |
| --- | --- | --- | --- | --- | --- | --- | --- | --- |
| 26 | 1922 | 1.584845346712973 | 1.445108287809881 | 1.738094504187224 | Cohort | RR | Both | Global |
| 27 | 1927 | 1.485179816674456 | 1.362643413256878 | 1.618735368620868 | Cohort | RR | Both | Global |
| 28 | 1932 | 1.422049150247688 | 1.311539004282183 | 1.541870870113353 | Cohort | RR | Both | Global |
| 29 | 1937 | 1.306641740396021 | 1.208866714730191 | 1.412324962662406 | Cohort | RR | Both | Global |
| 30 | 1942 | 1.224282255432004 | 1.134832204288431 | 1.320782962716063 | Cohort | RR | Both | Global |
| 31 | 1947 | 1.106835644621701 | 1.026729742837609 | 1.193191443757463 | Cohort | RR | Both | Global |
| 32 | 1952 | 1.045596705342178 | 0.970695244025544 | 1.126277765293808 | Cohort | RR | Both | Global |
| 33 | 1957 | 1 | 1 | 1 | Cohort | RR | Both | Global |
| 34 | 1962 | 0.9509127128708782 | 0.8744935133343724 | 1.03400994256856 | Cohort | RR | Both | Global |
| 35 | 1967 | 0.8711033544062573 | 0.791461582322087 | 0.9587591754378164 | Cohort | RR | Both | Global |
| 36 | 1972 | 0.9068346228351258 | 0.8109683458418068 | 1.014033454436381 | Cohort | RR | Both | Global |
| 37 | 1977 | 1.002764490703717 | 0.8878214039734731 | 1.132588850996353 | Cohort | RR | Both | Global |
| 38 | 1982 | 0.7854375657490692 | 0.6882297444298647 | 0.8963753378617465 | Cohort | RR | Both | Global |
| 39 | 1987 | 0.7233056032081019 | 0.6304773327538061 | 0.829801435282572 | Cohort | RR | Both | Global |
| 40 | 1992 | 0.6659970208545676 | 0.5813460965207679 | 0.7629741292523049 | Cohort | RR | Both | Global |
| 41 | 1997 | 0.6212697469678921 | 0.5407149822384772 | 0.7138254185220957 | Cohort | RR | Both | Global |
| 42 | 2002 | 0.5348235745300557 | 0.4650709335400615 | 0.6150379119499773 | Cohort | RR | Both | Global |
| 43 | 2007 | 0.447894438994106 | 0.3893097276971793 | 0.5152951858369358 | Cohort | RR | Both | Global |
| 44 | 2012 | 0.3730358390601467 | 0.3240593137606859 | 0.4294144044447141 | Cohort | RR | Both | Global |
| 45 | 2017 | 0.2883647651318765 | 0.2499635776676002 | 0.3326654168798153 | Cohort | RR | Both | Global |
| 46 | 1994.5 | 1.228616631183089 | 1.174362058267314 | 1.285377721285409 | Period | RR | Both | Global |
| 47 | 1999.5 | 1.080974626802604 | 1.034758709972613 | 1.129254706947049 | Period | RR | Both | Global |
| 48 | 2004.5 | 1 | 1 | 1 | Period | RR | Both | Global |
| 49 | 2009.5 | 0.9207278453064619 | 0.8815139378252691 | 0.9616861727837096 | Period | RR | Both | Global |
| 50 | 2014.5 | 0.8974727372245707 | 0.8585625122743415 | 0.9381463813598128 | Period | RR | Both | Global |

| 51 | 2019.5 0.8902396068362547 0.8509607396268191 0.9313315182173088 | Period RR | Both | Global |
| --- | --- | --- | --- | --- |
| 52 | 2.5 -3.311332404 -3.469730131 -3.15267476 | Local Drifts | Both | Global |
| 53 | 7.5 -2.622595417 -2.902578062 -2.341805435 | Local Drifts | Both | Global |
| 54 | 12.5 -2.13886424 -2.525005284 -1.751193522 | Local Drifts | Both | Global |
| 55 | 17.5 -2.220053669 -2.657192236 -1.780952038 | Local Drifts | Both | Global |
| 56 | 22.5 -1.812557713 -2.289357318 -1.333431465 | Local Drifts | Both | Global |
| 57 | 27.5 -1.285966722 -1.783089372 -0.786327897 | Local Drifts | Both | Global |
| 58 | 32.5 -0.897635773 -1.411533515 -0.381059311 | Local Drifts | Both | Global |
| 59 | 37.5 -0.574396017 -1.077286767 -0.068948734 | Local Drifts | Both | Global |
| 60 | 42.5 -0.336679081 -0.800508174 0.1293187470566171 | Local Drifts | Both | Global |
| 61 | 47.5 -0.90705091 -1.320321163 -0.492049884 | Local Drifts | Both | Global |
| 62 | 52.5 -1.25031736 -1.604287223 -0.895074122 | Local Drifts | Both | Global |
| 63 | 57.5 -1.279147431 -1.589405123 -0.967911594 | Local Drifts | Both | Global |
| 64 | 62.5 -1.435292461 -1.715714355 -1.154070475 | Local Drifts | Both | Global |
| 65 | 67.5 -1.458756839 -1.724605683 -1.192188836 | Local Drifts | Both | Global |
| 66 | 72.5 -1.39536761 -1.663245918 -1.126759577 | Local Drifts | Both | Global |
| 67 | 77.5 -1.34943383 -1.639054989 -1.058959889 | Local Drifts | Both | Global |
| 68 | 82.5 -1.206413791 -1.544011145 -0.867658844 | Local Drifts | Both | Global |
| 69 | 87.5 -1.03550957 -1.485054879 -0.583912887 | Local Drifts | Both | Global |
| 70 | 92.5 -0.894794752 -1.615145939 -0.169169319 | Local Drifts | Both | Global |
| 71 | 97.5 -0.634107916 -2.117632011 0.8719007568444459 | Local Drifts | Both | Global |
| 72 | 1 -1.278310722 -1.452856334 -1.103455957 | Net drift | Both | Global |
|  | age val low up | type | sex_name | location_name |
| 1 | 2.5 1.066544089076988 0.9319675048305094 1.220553600902571 | Age effect | Both | Global |
| 2 | 7.5 0.06522134123305916 0.05650178100054817 0.07528653569695215 | Age effect | Both | Global |
| 3 | 12.5 0.03039702604782253 0.02613850154777646 0.0353493558482354 | Age effect | Both | Global |

| 4 | 17.5 0.03625374805865286 | 0.03166249017831499 | 0.04151076686951292 | Age effect | Both | Global |
| --- | --- | --- | --- | --- | --- | --- |
| 5 | 22.5 0.026729659516079 | 0.02333594873643304 | 0.03061691238334122 | Age effect | Both | Global |
| 6 | 27.5 0.02455552662609665 | 0.02154899422851152 | 0.02798153275697414 | Age effect | Both | Global |
| 7 | 32.5 0.02768958477637925 | 0.02459416708567869 | 0.03117459121170055 | Age effect | Both | Global |
| 8 | 37.5 0.03588013620302002 | 0.03243000886300278 | 0.03969731181344072 | Age effect | Both | Global |
| 9 | 42.5 0.04962320436032607 | 0.04550258055575971 | 0.05411698371632214 | Age effect | Both | Global |
| 10 | 47.5 0.07166119386409309 | 0.06644590817593303 | 0.07728582311539792 | Age effect | Both | Global |
| 11 | 52.5 0.171088394436878 | 0.1614691270421565 | 0.1812807144448524 | Age effect | Both | Global |
| 12 | 57.5 0.2569917575846449 | 0.2437673612177862 | 0.270933578377785 | Age effect | Both | Global |
| 13 | 62.5 0.4177204612328633 | 0.3976614055325279 | 0.4387913468719133 | Age effect | Both | Global |
| 14 | 67.5 0.6353476632004694 | 0.6006928579449193 | 0.6720017523020251 | Age effect | Both | Global |
| 15 | 72.5 1.0326130994416 | 0.9759578322757356 | 1.092557257983182 | Age effect | Both | Global |
| 16 | 77.5 1.594250784208554 | 1.505430658103585 | 1.688311281073102 | Age effect | Both | Global |
| 17 | 82.5 2.504155380638629 | 2.361458397580127 | 2.6554751829663 | Age effect | Both | Global |
| 18 | 87.5 3.891862644327002 | 3.661512656633653 | 4.1367042156379 | Age effect | Both | Global |
| 19 | 92.5 5.730177287013039 | 5.363036016324041 | 6.122452215621329 | Age effect | Both | Global |
| 20 | 97.5 7.649276525511289 | 7.063089317967568 | 8.284113187538587 | Age effect | Both | Global |
| 21 | 1897 1.904521222198058 | 1.566165559126228 | 2.315975513997652 | Cohort RR | Both | Global |
| 22 | 1902 1.888126390364992 | 1.700772669054875 | 2.096118623527639 | Cohort RR | Both | Global |
| 23 | 1907 1.831079279671028 | 1.694973409276709 | 1.978114411760196 | Cohort RR | Both | Global |
| 24 | 1912 1.7498686347461 | 1.63518804216888 | 1.872592117788944 | Cohort RR | Both | Global |
| 25 | 1917 1.736694806314725 | 1.629349341289678 | 1.85111244952123 | Cohort RR | Both | Global |
| 26 | 1922 1.578669429644598 | 1.485367397598955 | 1.677832145853578 | Cohort RR | Both | Global |
| 27 | 1927 1.479225788552279 | 1.394322905090012 | 1.569298564579527 | Cohort RR | Both | Global |
| 28 | 1932 1.413838477516044 | 1.335176891412225 | 1.497134389729134 | Cohort RR | Both | Global |
| 29 | 1937 1.299763774709611 | 1.228771864066144 | 1.374857220816409 | Cohort RR | Both | Global |

| 30 | 1942 | 1.220559179635539 | 1.154396130060861 | 1.290514297647583 | Cohort RR | Both | Global |
| --- | --- | --- | --- | --- | --- | --- | --- |
| 31 | 1947 | 1.101508418760033 | 1.041503123547808 | 1.164970866785434 | Cohort RR | Both | Global |
| 32 | 1952 | 1.042436199004589 | 0.9851091022422853 | 1.103099368914237 | Cohort RR | Both | Global |
| 33 | 1957 | 1 | 1 | 1 | Cohort RR | Both | Global |
| 34 | 1962 | 0.953741472975509 | 0.8909880340736507 | 1.020914717692272 | Cohort RR | Both | Global |
| 35 | 1967 | 0.8750540120937779 | 0.8063772102370836 | 0.9495798174359216 | Cohort RR | Both | Global |
| 36 | 1972 | 0.9082804069765407 | 0.8210322111830552 | 1.004800160652328 | Cohort RR | Both | Global |
| 37 | 1977 | 0.991670929368488 | 0.8844593392577124 | 1.111878396784067 | Cohort RR | Both | Global |
| 38 | 1982 | 0.7966981327610123 | 0.7007262229497568 | 0.9058144164677487 | Cohort RR | Both | Global |
| 39 | 1987 | 0.729809970828116 | 0.6365350919728587 | 0.8367529147047342 | Cohort RR | Both | Global |
| 40 | 1992 | 0.6810023211179915 | 0.5946021736178724 | 0.7799570569113579 | Cohort RR | Both | Global |
| 41 | 1997 | 0.6179475373623212 | 0.5383658575543981 | 0.7092930459349815 | Cohort RR | Both | Global |
| 42 | 2002 | 0.5305200528723598 | 0.4615439657965402 | 0.6098043682879869 | Cohort RR | Both | Global |
| 43 | 2007 | 0.4477148708370335 | 0.3889027224623874 | 0.5154209368848219 | Cohort RR | Both | Global |
| 44 | 2012 | 0.3735793821638103 | 0.3240646436875039 | 0.4306596152848864 | Cohort RR | Both | Global |
| 45 | 2017 | 0.294125751848043 | 0.2543814146377605 | 0.3400797107106543 | Cohort RR | Both | Global |
| 46 | 1994.5 | 1.184799440455411 | 1.146893138312088 | 1.223958594930116 | Period RR | Both | Global |
| 47 | 1999.5 | 1.076625266953112 | 1.044976365802308 | 1.109232709346411 | Period RR | Both | Global |
| 48 | 2004.5 | 1 | 1 | 1 | Period RR | Both | Global |
| 49 | 2009.5 | 0.9136771130841342 | 0.8874954479920972 | 0.9406311535033264 | Period RR | Both | Global |
| 50 | 2014.5 | 0.8890092381586113 | 0.8621792611776112 | 0.9166741316090912 | Period RR | Both | Global |
| 51 | 2019.5 | 0.8647240916394084 | 0.8365025024539079 | 0.893897809591791 | Period RR | Both | Global |
| 52 | 2.5 | -3.30269361 | -3.500436626 | -3.104545388 | Local Drifts | Both | Global |
| 53 | 7.5 | -2.682808134 | -3.000955525 | -2.363617251 | Local Drifts | Both | Global |
| 54 | 12.5 | -2.223779963 | -2.650963293 | -1.794722083 | Local Drifts | Both | Global |
| 55 | 17.5 | -2.236945564 | -2.715660077 | -1.755875404 | Local Drifts | Both | Global |

| 56 | 22.5 | -1.77879862 | -2.2904104 -1.264508017 Local Drifts | Both | Global |
| --- | --- | --- | --- | --- | --- |
| 57 | 27.5 | -1.20910055 | -1.725089078 -0.690402845 Local Drifts | Both | Global |
| 58 | 32.5 | -0.871400181 | -1.386709327 -0.35339826 Local Drifts | Both | Global |
| 59 | 37.5 | -0.559647272 | -1.043376423 -0.073553509 Local Drifts | Both | Global |
| 60 | 42.5 | -0.356127521 | -0.781519497 0.07108829162199015 Local Drifts | Both | Global |
| 61 | 47.5 | -0.874364351 | -1.233514021 -0.513908687 Local Drifts | Both | Global |
| 62 | 52.5 | -1.214046712 | -1.501749932 -0.925503141 Local Drifts | Both | Global |
| 63 | 57.5 | -1.249710143 | -1.485582606 -1.013272932 Local Drifts | Both | Global |
| 64 | 62.5 | -1.416182956 | -1.614107709 -1.217860033 Local Drifts | Both | Global |
| 65 | 67.5 | -1.453081459 | -1.625171439 -1.280690437 Local Drifts | Both | Global |
| 66 | 72.5 | -1.396029092 | -1.5528801 -1.238928181 Local Drifts | Both | Global |
| 67 | 77.5 | -1.35743594 | -1.508336641 -1.206304042 Local Drifts | Both | Global |
| 68 | 82.5 | -1.231711993 | -1.387972713 -1.075203661 Local Drifts | Both | Global |
| 69 | 87.5 | -1.075575716 | -1.264008734 -0.886783083 Local Drifts | Both | Global |
| 70 | 92.5 | -0.951364156 | -1.234694166 -0.667221352 Local Drifts | Both | Global |
| 71 | 97.5 | -0.702895207 | -1.268528596 -0.1340213 Local Drifts | Both | Global |
| 72 | 1 | -1.271451708 | -1.428778967 -1.113873342 Net drift | Both | Global |

Table S4 The temporal trend and forecast of LRI caused by CMV globally from 1990 to 2021:Projected age-standardized mortality rate

|  | val | sd | T  i  m  e | gr  ou  p | low_50 | up_50 | low_60 | up_60 | low_70 | up_70 | low_80 | up_80 | low_95 | up_95 |
| --- | --- | --- | --- | --- | --- | --- | --- | --- | --- | --- | --- | --- | --- | --- |
| 1 | 0.40294  3210923  5497 | 0.002955  24533807  8156 | 1  9  9  0 | AS  R | 0.40095  1375565  6851 | 0.40493  5046281  4144 | 0.40045  7849594  226 | 0.40542  8572252  8735 | 0.39988  1576753  3008 | 0.40600  4845093  7987 | 0.39915  4586400  1335 | 0.40673  1835446  966 | 0.39715  0930060  9166 | 0.40873  5491786  1829 |
| 2 | 0.39706  4136770  106 | 0.002678  79863291  3908 | 1  9  9  1 | AS  R | 0.39525  8626491  522 | 0.39886  9647048  69 | 0.39481  1267119  8254 | 0.39931  7006420  3866 | 0.39428  8901386  4072 | 0.39983  9372153  8048 | 0.39362  9916922  7104 | 0.40049  8356617  5016 | 0.39181  3691449  5947 | 0.40231  4582090  6173 |
| 3 | 0.39391  6083170  2351 | 0.002614  65013684  8702 | 1  9  9  2 | AS  R | 0.39215  3808977  9991 | 0.39567  8357362  4711 | 0.39171  7162405  1453 | 0.39611  5003935  3248 | 0.39120  7305628  4598 | 0.39662  4860712  0103 | 0.39056  4101694  7951 | 0.39726  8064645  6751 | 0.38879  1368902  0116 | 0.39904  0797438  4585 |
| 4 | 0.39381  6984418  9282 | 0.002586  01709187  2532 | 1  9  9  3 | AS  R | 0.39207  4008899  0061 | 0.39555  9959938  8502 | 0.39164  2144044  6634 | 0.39599  1824793  1929 | 0.39113  7870711  7482 | 0.39649  6098126  1081 | 0.39050  1710507  1475 | 0.39713  2258330  7088 | 0.38874  8390918  858 | 0.39888  5577918  9983 |
| 5 | 0.39441  2409385  1737 | 0.002578  30172381  3932 | 1  9  9  4 | AS  R | 0.39267  4634023  3231 | 0.39615  0184747  0243 | 0.39224  4057635  4462 | 0.39658  0761134  9012 | 0.39174  1288799  3025 | 0.39708  3529971  0449 | 0.39110  7026575  2442 | 0.39771  7792195  1032 | 0.38935  8938006  4984 | 0.39946  5880763  849 |
| 6 | 0.38865 | 0.002525 1 AS 0.38695 | | | | 0.39035 | 0.38653 | 0.39078 | 0.38604 | 0.39127 | 0.38541 | 0.39189 | 0.38370 | 0.39360 |

|  | 6388269  2068 | 43730265  6905 | 9  9  5  1 | R 4243527  216 | 8533011  1976 | 2495497  6723 | 0281040  7413 | 0035223  6542 | 2741314  7594 | 8777647  2006 | 3998891  213 | 6531155  9992 | 6245382  4143 |
| --- | --- | --- | --- | --- | --- | --- | --- | --- | --- | --- | --- | --- | --- |
|  | 0.38166 | 0.002476 |  | 0.37999 | 0.38333 | 0.37958 | 0.38375 | 0.37910 | 0.38423 | 0.37849 | 0.38484 | 0.37681 | 0.38652 |
|  |  |  | 9 | AS |  |  |  |  |  |  |  |  |  |
| 7 | 7495270 | 78617998 |  | 8141384 | 6849155 | 4518092 | 0472447 | 1544787 | 3445752 | 2255387 | 2735152 | 2994357 | 1996182 |
|  |  |  | 9 | R |  |  |  |  |  |  |  |  |  |
|  | 1667 | 9481 |  | 8538 | 4796 | 7955 | 5378 | 6976 | 6358 | 4202 | 9132 | 3873 | 9461 |
|  |  |  | 6  1 |  |  |  |  |  |  |  |  |  |  |
|  | 0.37478 | 0.002430 |  | 0.37314 | 0.37641 | 0.37273 | 0.37682 | 0.37226 | 0.37729 | 0.37166 | 0.37789 | 0.37001 | 0.37954 |
|  |  |  | 9 | AS |  |  |  |  |  |  |  |  |  |
| 8 | 0700661 | 98272550 |  | 2218304 | 9183018 | 6244189 | 5157133 | 2202557 | 9198765 | 4180807 | 7220515 | 5974519 | 5426803 |
|  |  |  | 9 | R |  |  |  |  |  |  |  |  |  |
|  | 4275 | 0392 |  | 4402 | 4148 | 2816 | 5733 | 8091 | 0459 | 336 | 519 | 4467 | 4082 |
|  |  |  | 7  1 |  |  |  |  |  |  |  |  |  |  |
|  | 0.36919 | 0.002391 |  | 0.36758 | 0.37080 | 0.36718 | 0.37120 | 0.36671 | 0.37167 | 0.36612 | 0.37225 | 0.36450 | 0.37388 |
|  |  |  | 9 | AS |  |  |  |  |  |  |  |  |  |
| 9 | 2642035 | 97674251 |  | 0449711 | 4834360 | 0989595 | 4294476 | 4554130 | 0729941 | 6127852 | 9156219 | 4367620 | 0916451 |
|  |  |  | 9 | R |  |  |  |  |  |  |  |  |  |
|  | 9695 | 8913 |  | 5118 | 4273 | 5111 | 4279 | 7199 | 2191 | 0603 | 8788 | 6324 | 3066 |
|  |  |  | 8  1 |  |  |  |  |  |  |  |  |  |  |
|  | 0.36242 | 0.002346 |  | 0.36084 | 0.36401 | 0.36045 | 0.36440 | 0.35999 | 0.36486 | 0.35942 | 0.36543 | 0.35783 | 0.36702 |
|  |  |  | 9 | AS |  |  |  |  |  |  |  |  |  |
| 10 | 9553122 | 66864649 |  | 7898454 | 1207790 | 6004790 | 3101454 | 8404404 | 0701840 | 1123917 | 7982327 | 0082575 | 9023669 |
|  |  |  | 9 | R |  |  |  |  |  |  |  |  |  |
|  | 5151 | 8138 |  | 7753 | 2548 | 8101 | 22 | 743 | 2871 | 7045 | 3257 | 3787 | 6514 |
|  |  |  | 9  2 |  |  |  |  |  |  |  |  |  |  |
|  | 0.35528 | 0.002299 |  | 0.35373 | 0.35683 | 0.35335 | 0.35721 | 0.35290 | 0.35766 | 0.35233 | 0.35823 | 0.35077 | 0.35979 |
|  |  |  | 0 | AS |  |  |  |  |  |  |  |  |  |
| 11 | 4213044 | 04401724 |  | 4657377 | 3768712 | 0717026 | 7709063 | 2403442 | 6022646 | 6838614 | 1587474 | 8086770 | 0339318 |
|  |  |  | 0 | R |  |  |  |  |  |  |  |  |  |
|  | 6655 | 0908 |  | 0451 | 2858 | 1658 | 1651 | 8039 | 527 | 5626 | 7683 | 8733 | 4576 |
|  |  |  | 0 |  |  |  |  |  |  |  |  |  |  |
|  | 0.34774 | 0.002250 | 2 | 0.34622 AS | 0.34925 | 0.34585 | 0.34963 | 0.34541 | 0.35007 | 0.34485 | 0.35062 | 0.34333 | 0.35215 |
| 12 | 3114554 | 25468949 | 0 | 6442893 R | 9786215 | 0650360 | 5578748 | 1850696 | 4378412 | 8288042 | 7941066 | 2615363 | 3613746 |
|  | 6271 | 672 | 0 | 9064 | 3479 | 7604 | 4939 | 3085 | 9458 | 6924 | 5619 | 2136 | 0407 |

13

14

15

16

17

18

19

0.34245 8223636 8086

0.33843 0660827 6952

0.33215 1977277 0799

0.32460 4673178 3892

0.31510 5130368 2438

0.30866 5662897 0612

0.30408

1

2 0.002210

0.34096 AS

8277140 R

3311

0.33696 AS

2270105 R

0488

0.33071 AS

2942536 R

5624

0.32319 AS

9608796 R

2568

0.31373 AS

9910205 R

8936

0.30733 AS

1626729 R

7262

0

60311050

0

0762

2

2 0.002178

0

62125021

0

7264

3

2 0.002135

0

06638058

0

9848

4

2 0.002084

0

66525538

0

9392

5

2 0.002025

0

54920229

0

9889

6

2

0 001979

. 0

28214738

0

1259

7

0.001940 2 AS 0.30277

0.34394 8170133 286

0.33989 9051550 3416

0.33359 1012017 5974

0.32600 9737560 5217

0.31647 0350530 5939

0.30999 9699064 3961

0.30539

0.34059 9106420 8774

0.33659 8440356 2625

0.33035 6386451 0038

0.32285 1469698 6067

0.31340 1643489 1096

0.30700 1086611 1135

0.30245

0.34431 7340852 7397

0.34026 2881299 1279

0.33394 7568103 156

0.32635 7876658 1717

0.31680 8617247 378

0.31033 0239183 0088

0.30571

0.34016 8038814 3298

0.33617 3609212 4701

0.32994 0048506 7888

0.32244 4959973 8058

0.31300 6661394 6611

0.30661 5126592 3742

0.30207

0.34474 8408459 2873

0.34068 7712442 9203

0.33436 3906047 371

0.32676 4386382 9726

0.31720 3599341 8265

0.31071 6199201 7481

0.30609

0.33962 4230449 1466

0.33563 7668384 9167

0.32941 4822177 1637

0.32193 2132320 98

0.31250 8376290 8953

0.30612 8223184 1184

0.30159

0.34529 2216824 4705

0.34122 3653270 4737

0.33488 9132376 9961

0.32727 7214035 7984

0.31770 1884445 5922

0.31120 3102610 0039

0.30657

0.33812 5441540 2271

0.33416 0563177 2693

0.32796 7247171 1238

0.32051 8729277 826

0.31113 5053931 736

0.30478 6269888 1939

0.30028

0.34679 1005733 3901

0.34270 0758478 121

0.33633 6707383 036

0.32869 0617078 9524

0.31907 5206804 7515

0.31254 5055905 9284

0.30788

|  | 5113748  2786 | 80654990  3487 | 0  0  8  2 | R 7010133  6436 | 3217362  9135 | 2895439  8097 | 7332056  7474 | 4438162  5786 | 5789333  9786 | 6999751  3023 | 3227745  2549 | 1132910  4677 | 9094586  0894 |
| --- | --- | --- | --- | --- | --- | --- | --- | --- | --- | --- | --- | --- | --- |
|  | 0.29869 | 0.001899 |  | 0.29741 | 0.29997 | 0.29709 | 0.30029 | 0.29672 | 0.30066 | 0.29626 | 0.30113 | 0.29497 | 0.30242 |
|  |  |  | 0 | AS |  |  |  |  |  |  |  |  |  |
| 20 | 6753570 | 93557721 |  | 6196991 | 7310149 | 8907749 | 4599390 | 8420312 | 5086828 | 1036160 | 2470980 | 2879838 | 0627301 |
|  |  |  | 0 | R |  |  |  |  |  |  |  |  |  |
|  | 2515 | 7805 |  | 2067 | 2963 | 8113 | 6916 | 2538 | 2492 | 2583 | 2447 | 9046 | 5984 |
|  |  |  | 9  2 |  |  |  |  |  |  |  |  |  |  |
|  | 0.29400 | 0.001862 |  | 0.29275 | 0.29526 | 0.29244 | 0.29557 | 0.29207 | 0.29593 | 0.29162 | 0.29639 | 0.29035 | 0.29765 |
|  |  |  | 0 | AS |  |  |  |  |  |  |  |  |  |
| 21 | 9007984 | 27588779 |  | 3834036 | 4181932 | 2833962 | 5182006 | 9690164 | 8325804 | 1570296 | 6445672 | 8947244 | 9068724 |
|  |  |  | 1 | R |  |  |  |  |  |  |  |  |  |
|  | 5303 | 176 |  | 1587 | 902 | 8974 | 1632 | 7781 | 2826 | 3813 | 6794 | 4585 | 6022 |
|  |  |  | 0  2 |  |  |  |  |  |  |  |  |  |  |
|  | 0.29049 | 0.001828 |  | 0.28926 | 0.29173 | 0.28896 | 0.29203 | 0.28860 | 0.29239 | 0.28815 | 0.29284 | 0.28691 | 0.29408 |
|  |  |  | 0 | AS |  |  |  |  |  |  |  |  |  |
| 22 | 7943242 | 54656023 |  | 5502860 | 0383623 | 0135585 | 5750899 | 3569005 | 2317478 | 3746551 | 2139932 | 3991984 | 1894500 |
|  |  |  | 1 | R |  |  |  |  |  |  |  |  |  |
|  | 1996 | 8046 |  | 5992 | 8 | 0394 | 3598 | 793 | 6062 | 9745 | 4248 | 1331 | 2662 |
|  |  |  | 1  2 |  |  |  |  |  |  |  |  |  |  |
|  | 0.28702 | 0.001795 |  | 0.28581 | 0.28823 | 0.28551 | 0.28853 | 0.28516 | 0.28888 | 0.28472 | 0.28932 | 0.28350 | 0.29054 |
|  |  |  | 0 | AS |  |  |  |  |  |  |  |  |  |
| 23 | 6207147 | 75783578 |  | 5866366 | 6547929 | 5974808 | 6439487 | 5802030 | 6612265 | 4045602 | 8368693 | 6521789 | 5892506 |
|  |  |  | 1 | R |  |  |  |  |  |  |  |  |  |
|  | 9395 | 7902 |  | 6185 | 2606 | 0419 | 8371 | 0633 | 8158 | 4595 | 4196 | 7952 | 0838 |
|  |  |  | 2  2 |  |  |  |  |  |  |  |  |  |  |
|  | 0.28609 | 0.001774 |  | 0.28490 | 0.28729 | 0.28460 | 0.28758 | 0.28425 | 0.28793 | 0.28382 | 0.28837 | 0.28262 | 0.28957 |
|  |  |  | 0 | AS |  |  |  |  |  |  |  |  |  |
| 24 | 7651165 | 07738659 |  | 1923006 | 3379323 | 5652083 | 9650247 | 9706992 | 5595337 | 3283955 | 2018374 | 0459487 | 4842843 |
|  |  |  | 1 | R |  |  |  |  |  |  |  |  |  |
|  | 3125 | 7514 |  | 7458 | 8792 | 184 | 441 | 7975 | 8275 | 6945 | 9305 | 5814 | 0436 |
|  |  |  | 3 |  |  |  |  |  |  |  |  |  |  |
|  | 0.28480 | 0.001754 | 2 | 0.28361 AS | 0.28598 | 0.28332 | 0.28627 | 0.28298 | 0.28661 | 0.28255 | 0.28705 | 0.28136 | 0.28823 |
| 25 | 0908669 | 60636510 | 0 | 8303979 R | 3513359 | 5284716 | 6532622 | 3136475 | 8680864 | 1503309 | 0314029 | 1880194 | 9937145 |
|  | 8839 | 9564 | 1 | 8 | 9677 | 8267 | 9411 | 6304 | 1374 | 8134 | 9543 | 2692 | 4986 |

26

27

28

29

30

31

32

0.28257 8898133 7592

0.27972 9627941 0736

0.27625 2840279 186

0.27268 0378904 5391

0.26710 7746288 9233

0.25134 4004579 493

0.24274

4

2 0.001728

0.28141 AS

3697316 R

2966

0.27858 AS

4754209 R

5082

0.27512 AS

9691577 R

5746

0.27157 AS

6522077 R

7842

0.26602 AS

3809387 R

3116

0.25030 AS

8029043 R

0606

0

78459564

1

1954

5

2 0.001698

0

62571448

1

8758

6

2 0.001666

0

39273236

1

1184

7

2 0.001637

0

76977263

1

3413

8

2 0.001608

0

21498755

1

4424

9

2

0 001537

. 0

05569203

2

6266

0

0.001605 2 AS 0.24166

0.28374 4098951 2219

0.28087 4501672 639

0.27737 5988980 7975

0.27378 4235731 294

0.26819 1683190 5349

0.25237 9980115 9254

0.24382

0.28112 4990288 8244

0.27830 1083715 1885

0.27485 1403991 2702

0.27130 3014525 7544

0.26575 5237484 39

0.25005 1340742 4905

0.24139

0.28403 2805978 6941

0.28115 8172166 9587

0.27765 4276567 1018

0.27405 7743283 3238

0.26846 0255093 4565

0.25263 6668416 4955

0.24409

0.28078 7877292 6742

0.27796 9851700 8632

0.27452 6457408 4598

0.27098 3649420 0909

0.26544 1635561 8169

0.24975 1614882 5434

0.24108

0.28436 9918974 8443

0.28148 9404181 2839

0.27797 9223149 9122

0.27437 7108388 9873

0.26877 3857016 0296

0.25293 6394276 4426

0.24440

0.28036 2596282 1463

0.27755 1989775 099

0.27411 6524796 2989

0.27058 0758056 0231

0.26504 6014674 8785

0.24937 3499182 3025

0.24068

0.28479 5199985 3722

0.28190 7266107 0482

0.27838 9155762 0731

0.27477 9999753 0552

0.26916 9477902 968

0.25331 4509976 6835

0.24480

0.27919 0480326 301

0.27640 0321540 6756

0.27298 6710523 7581

0.26947 0350150 1776

0.26395 5644913 3166

0.24833 1375423 1019

0.23960

0.28596 7315941 2175

0.28305 8934341 4716

0.27951 8970034 6139

0.27589 0407658 9006

0.27025 9847664 5299

0.25435 6633735 8841

0.24589

|  | 7066474  0563 | 06905826  5479 | 0  2  1  2 | R 5249928  7854 | 8883019  3273 | 7203396  0551 | 6929552  0576 | 4214929  6933 | 9918018  4194 | 9367941  36 | 4765006  7527 | 1131119  856 | 3001828  2567 |
| --- | --- | --- | --- | --- | --- | --- | --- | --- | --- | --- | --- | --- | --- |
|  | 0.23931 | 0.003870 |  | 0.23670 | 0.24192 | 0.23606 | 0.24257 | 0.23530 | 0.24332 | 0.23435 | 0.24427 | 0.23173 | 0.24690 |
|  |  |  | 0 | AS |  |  |  |  |  |  |  |  |  |
| 33 | 7456763 | 42820049 |  | 8788156 | 6125370 | 2426646 | 2486879 | 7693147 | 7220379 | 5567810 | 9345716 | 1417490 | 3496036 |
|  |  |  | 2 | R |  |  |  |  |  |  |  |  |  |
|  | 3701 | 6675 |  | 2353 | 5049 | 7524 | 9878 | 6555 | 0847 | 3334 | 4068 | 3966 | 3436 |
|  |  |  | 2  2 |  |  |  |  |  |  |  |  |  |  |
|  | 0.23501 | 0.005017 |  | 0.23163 | 0.23839 | 0.23079 | 0.23923 | 0.22981 | 0.24021 | 0.22858 | 0.24144 | 0.22517 | 0.24484 |
|  |  |  | 0 | AS |  |  |  |  |  |  |  |  |  |
| 34 | 3564806 | 57391456 |  | 1719988 | 5409625 | 3785144 | 3344468 | 5358231 | 1771382 | 1035048 | 6094565 | 9119934 | 8009679 |
|  |  |  | 2 | R |  |  |  |  |  |  |  |  |  |
|  | 5849 | 9727 |  | 1649 | 0049 | 4317 | 738 | 0906 | 0791 | 1065 | 0632 | 0282 | 1415 |
|  |  |  | 3  2 |  |  |  |  |  |  |  |  |  |  |
|  | 0.23082 | 0.005938 |  | 0.22682 | 0.23482 | 0.22583 | 0.23581 | 0.22467 | 0.23697 | 0.22321 | 0.23843 | 0.21918 | 0.24246 |
|  |  |  | 0 | AS |  |  |  |  |  |  |  |  |  |
| 35 | 4426134 | 59911493 |  | 1810330 | 7041937 | 0064278 | 8787989 | 2037451 | 6814817 | 1142068 | 7710199 | 4771869 | 4080399 |
|  |  |  | 2 | R |  |  |  |  |  |  |  |  |  |
|  | 3409 | 3251 |  | 8759 | 8059 | 682 | 9998 | 27 | 4118 | 9965 | 6853 | 0717 | 6101 |
|  |  |  | 4  2 |  |  |  |  |  |  |  |  |  |  |
|  | 0.22675 | 0.006725 |  | 0.22221 | 0.23128 | 0.22109 | 0.23240 | 0.21978 | 0.23372 | 0.21813 | 0.23537 | 0.21357 | 0.23993 |
|  |  |  | 0 | AS |  |  |  |  |  |  |  |  |  |
| 36 | 3046340 | 59235102 |  | 9997095 | 6095584 | 6823172 | 9269507 | 5332664 | 0760015 | 0836946 | 5255734 | 0885332 | 5207348 |
|  |  |  | 2 | R |  |  |  |  |  |  |  |  |  |
|  | 1795 | 8698 |  | 5862 | 7729 | 9644 | 3947 | 5138 | 8452 | 1607 | 1983 | 1633 | 1958 |
|  |  |  | 5  2 |  |  |  |  |  |  |  |  |  |  |
|  | 0.22282 | 0.007423 |  | 0.21782 | 0.22782 | 0.21658 | 0.22906 | 0.21513 | 0.23051 | 0.21330 | 0.23234 | 0.20827 | 0.23737 |
|  |  |  | 0 | AS |  |  |  |  |  |  |  |  |  |
| 37 | 5390489 | 36259102 |  | 2044103 | 8736875 | 2342550 | 8438428 | 4786845 | 5994133 | 8639647 | 2141331 | 5599811 | 5181168 |
|  |  |  | 2 | R |  |  |  |  |  |  |  |  |  |
|  | 6149 | 3747 |  | 2649 | 9649 | 564 | 6659 | 3143 | 9155 | 9225 | 3074 | 2084 | 0215 |
|  |  |  | 6 |  |  |  |  |  |  |  |  |  |  |
|  | 0.21907 | 0.008059 | 2 | 0.21364 AS | 0.22450 | 0.21229 | 0.22585 | 0.21072 | 0.22742 | 0.20874 | 0.22940 | 0.20327 | 0.23487 |
| 38 | 5234302 | 42065706 | 0 | 3184779 R | 7283824 | 7261529 | 3207074 | 5674501 | 4794102 | 3057019 | 7411584 | 8769814 | 1698789 |
|  | 1141 | 5688 | 2 | 2519 | 9764 | 5219 | 7064 | 3941 | 8342 | 7559 | 4724 | 2654 | 9629 |

39

40

41

42

43

44

45

0.21546 8515799 735

0.21199 9563112 4211

0.20862 0210982 9454

0.20537 9734646 6326

0.20225 7231033 9925

0.19929 0248078 3204

0.19644

7

2 0.008642

0.20964 AS

3156990 R

0472

0.20581 AS

0635762 R

5946

0.20209 AS

4505458 R

0296

0.19853 AS

7295166 R

4398

0.19511 AS

4444265 R

1638

0.19186 AS

1198817 R

85

0

96559300

2

8534

8

2 0.009182

0

38479202

2

7366

9

2 0.009682

0

05567494

3

924

0

2

0.010151

0

98735933

3

659

1

2

0.010597

0

60648194

3

17

2

2

0 011022

. 0

32827962

3

967

3

0.011425 2 AS 0.18874

0.22129 3874609 4227

0.21818 8490462 2476

0.21514 5916507 8612

0.21222 2174126 8255

0.20940 0017802 8212

0.20671 9297338 7907

0.20414

0.20819 9781736 0148

0.20427 7177502 3261

0.20047 7602160 3131

0.19684 1913277 4306

0.19334 4643982 6795

0.19002 0469995 1518

0.18683

0.22273 7249863 4551

0.21972 1948722 5161

0.21676 2819805 5778

0.21391 7556015 8347

0.21116 9818085 3054

0.20856 0026161 4889

0.20605

0.20651 4403445 3781

0.20248 6612467 8807

0.19858 9601303 698

0.19486 2275742 3599

0.19127 8110718 7009

0.18787 1115980 624

0.18460

0.22442 2628154 0918

0.22151 2513756 9615

0.21865 0820662 1929

0.21589 7193550 9054

0.21323 6351349 2841

0.21070 9380176 0167

0.20828

0.20438 8233909 498

0.20022 7745809 042

0.19620 7815607 6605

0.19236 4886851 9631

0.18867 1099524 1432

0.18515 9623223 8351

0.18179

0.22654 8797689 9719

0.22377 1380415 8002

0.22103 2606358 2304

0.21839 4582441 3022

0.21584 3362543 8417

0.21342 0872932 8056

0.21109

0.19852 8303237 4383

0.19400 2088920 0475

0.18964 3381860 0449

0.18548 1839422 3329

0.18148 5922329 3867

0.17768 6484650 2462

0.17404

0.23240 8728362 0317

0.22999 7037304 7947

0.22759 7040105 846

0.22527 7629870 9324

0.22302 8539738 5982

0.22089 4011506 3945

0.21883

|  | 2949892  891 | 83731964  087 | 0  3  4  2 | R 1935539  453 | 3964246  3289 | 3820707  073 | 2079078  7089 | 5782429  743 | 0117356  0389 | 5026449  1114 | 0873336  6706 | 8308746  3948 | 7591039  3871 |
| --- | --- | --- | --- | --- | --- | --- | --- | --- | --- | --- | --- | --- | --- |
|  | 0.19370 | 0.011810 |  | 0.18574 | 0.20166 | 0.18377 | 0.20363 | 0.18147 | 0.20594 | 0.17856 | 0.20884 | 0.17055 | 0.21685 |
|  |  |  | 0 | AS |  |  |  |  |  |  |  |  |  |
| 46 | 7331073 | 16398529 |  | 7280547 | 7381599 | 4983162 | 9678985 | 2001184 | 2660962 | 6700844 | 7961302 | 9409662 | 5252484 |
|  |  |  | 3 | R |  |  |  |  |  |  |  |  |  |
|  | 6349 | 473 |  | 5463 | 7236 | 0021 | 2678 | 8696 | 4003 | 4871 | 7828 | 4573 | 8126 |
|  |  |  | 5  2 |  |  |  |  |  |  |  |  |  |  |
|  | 0.19107 | 0.012178 |  | 0.18286 | 0.19928 | 0.18083 | 0.20131 | 0.17846 | 0.20369 | 0.17546 | 0.20669 | 0.16720 | 0.21494 |
|  |  |  | 0 | AS |  |  |  |  |  |  |  |  |  |
| 47 | 7293329 | 50670518 |  | 8979809 | 5606848 | 5169189 | 9417468 | 0360382 | 4226275 | 4447732 | 0138925 | 7420186 | 7166471 |
|  |  |  | 3 | R |  |  |  |  |  |  |  |  |  |
|  | 0037 | 849 |  | 7067 | 3008 | 9402 | 0673 | 4284 | 579 | 9521 | 0554 | 8343 | 1732 |
|  |  |  | 6  2 |  |  |  |  |  |  |  |  |  |  |
|  | 0.18857 | 0.012536 |  | 0.18012 | 0.19702 | 0.17803 | 0.19911 | 0.17558 | 0.20156 | 0.17250 | 0.20464 | 0.16400 | 0.21314 |
|  |  |  | 0 | AS |  |  |  |  |  |  |  |  |  |
| 48 | 6092932 | 49276665 |  | 6496807 | 5689057 | 2902515 | 9283349 | 8286426 | 3899438 | 4309205 | 7876659 | 4567109 | 7618755 |
|  |  |  | 3 | R |  |  |  |  |  |  |  |  |  |
|  | 3707 | 627 |  | 6444 | 0971 | 6128 | 1287 | 1148 | 6266 | 5174 | 2241 | 7244 | 017 |
|  |  |  | 7  2 |  |  |  |  |  |  |  |  |  |  |
|  | 0.18620 | 0.012883 |  | 0.17751 | 0.19488 | 0.17536 | 0.19703 | 0.17285 | 0.19955 | 0.16968 | 0.20271 | 0.16094 | 0.21145 |
|  |  |  | 0 | AS |  |  |  |  |  |  |  |  |  |
| 49 | 2453401 | 98056924 |  | 8650497 | 6256304 | 7025742 | 7881059 | 4649531 | 0257270 | 5190311 | 9716490 | 9851485 | 5055316 |
|  |  |  | 3 | R |  |  |  |  |  |  |  |  |  |
|  | 041 | 458 |  | 3701 | 7118 | 3063 | 7757 | 3036 | 7784 | 2694 | 8125 | 3216 | 7603 |
|  |  |  | 8  2 |  |  |  |  |  |  |  |  |  |  |
|  | 0.18395 | 0.013221 |  | 0.17504 | 0.19286 | 0.17283 | 0.19507 | 0.17025 | 0.19764 | 0.16700 | 0.20090 | 0.15803 | 0.20986 |
|  |  |  | 0 | AS |  |  |  |  |  |  |  |  |  |
| 50 | 2138321 | 69034837 |  | 0719026 | 3557616 | 2696738 | 1579904 | 4467120 | 9809522 | 1931294 | 2345347 | 7625238 | 6651404 |
|  |  |  | 3 | R |  |  |  |  |  |  |  |  |  |
|  | 2181 | 075 |  | 4162 | 0199 | 2383 | 1979 | 306 | 1302 | 6068 | 8294 | 4114 | 0247 |
|  |  |  | 9 |  |  |  |  |  |  |  |  |  |  |
|  | 0.18174 | 0.013545 | 2 | 0.17262 AS | 0.19087 | 0.17035 | 0.19314 | 0.16771 | 0.19578 | 0.16438 | 0.19911 | 0.15520 | 0.20829 |
| 51 | 9884452 | 08143617 | 0 | 0499564 R | 9269340 | 8470964 | 1297940 | 7180084 | 2588820 | 5090051 | 4678853 | 1524837 | 8244067 |
|  | 5516 | 581 | 4 | 5691 | 534 | 7277 | 3754 | 6734 | 4297 | 3742 | 7289 | 647 | 4561 |

52

53

54

55

56

57

58

0.17961 9388294 9387

0.17755 0775969 7508

0.17559 0940242 8329

0.17370 0442296 3099

0.17183 8834715 3058

0.16999 6057819 3016

0.16820

2 0.013858

0.17027 AS

8811815 R

7085

0.16800 AS

4709096 R

7945

0.16584 AS

2576471 R

8534

0.16375 AS

5059793 R

1359

0.16170 AS

3607646 R

7691

0.15967 AS

7855393 R

2652

0

42207600

4

923

1

2

0.014163

0

30396581

4

052

2

2

0.014463

0

44773142

4

36

3

2

0.014755

0

76039046

4

585

4

2

0.015037

0

42888506

4

928

5

2

0.015308

0

90567661

4

192

6

0.015575 2 AS 0.15771

0.18895 9964774 169

0.18709 6842842 7071

0.18533 9304013 8124

0.18364 5824799 4839

0.18197 4061783 8425

0.18031 4260245 338

0.17870

0.16796 4455329 015

0.16563 9437334 5042

0.16342 7180700 7056

0.16129 0847807 9281

0.15919 2357022 9626

0.15712 1268145 2709

0.15511

0.19127 4321260 8625

0.18946 2114604 9975

0.18775 4699784 9601

0.18611 0036784 6917

0.18448 5312407 6491

0.18287 0847493 3322

0.18130

0.16526 2063024 1932

0.16287 7593061 1711

0.16060 6808393 078

0.15841 3474531 7873

0.15626 0058390 374

0.15413 6031538 3316

0.15207

0.19397 6713565 6843

0.19222 3958878 3305

0.19057 5072092 5877

0.18898 7410060 8325

0.18741 7611040 2376

0.18585 6084100 2715

0.18434

0.16185 2891193 4949

0.15939 3420285 5817

0.15704 8800251 1478

0.15478 3557475 7327

0.15256 0850884 647

0.15037 0040741 8851

0.14824

0.19738 5885396 3826

0.19570 8131653 9199

0.19413 3080234 5179

0.19261 7327116 8871

0.19111 6818545 9646

0.18962 2074896 7181

0.18817

0.15245 6881025 9606

0.14979 0700196 7622

0.14724 2582689 2426

0.14477 9151930 9969

0.14236 5474100 57

0.13999 0602693 1422

0.13768

0.20678 1895563 9168

0.20531 0851742 7394

0.20393 9297796 4231

0.20262 1732661 6229

0.20131 2195330 0416

0.20000 1512945 4609

0.19873

|  | 9312068  9388 | 49565008  601 | 0  4  7 | R | 1428000  7808 | 7196137  0968 | 0320227  2165 | 8303910  6611 | 3098575  4497 | 5525562  4279 | 1526645  5285 | 7097492  3491 | 1340594  7702 | 7283543  1074 |
| --- | --- | --- | --- | --- | --- | --- | --- | --- | --- | --- | --- | --- | --- | --- |
| 59 | 0.16648  8026861  7844 | 0.015837  68252351  16 | 2  0  4  8 | AS  R | 0.15581  3428840  9376 | 0.17716  2624882  6313 | 0.15316  8535859  5112 | 0.17980  7517864  0577 | 0.15008  0187767  4264 | 0.18289  5865956  1425 | 0.14618  4117866  6426 | 0.18679  1935856  9263 | 0.13544  6169115  7017 | 0.19752  9884607  8672 |
| 60 | 0.16482  6227240  162 | 0.016095  15118701  017 | 2  0  4  9 | AS  R | 0.15397  8095340  1172 | 0.17567  4359140  2069 | 0.15129  0205091  8865 | 0.17836  2249388  4376 | 0.14815  1650610  4195 | 0.18150  0803869  9046 | 0.14419  2243418  415 | 0.18546  0211061  9091 | 0.13327  9730913  6221 | 0.19637  2723566  702 |
| 61 | 0.16312  9929624  4385 | 0.016338  45061223  66 | 2  0  5  0 | AS  R | 0.15211  7813911  7911 | 0.17414  2045337  086 | 0.14938  9292659  5476 | 0.17687  0566589  3295 | 0.14620  3294790  1614 | 0.18005  6564458  7157 | 0.14218  4035939  5512 | 0.18407  5823309  3259 | 0.13110  6566424  4548 | 0.19515  3292824  4223 |

Note: ASR, age-standardized rates


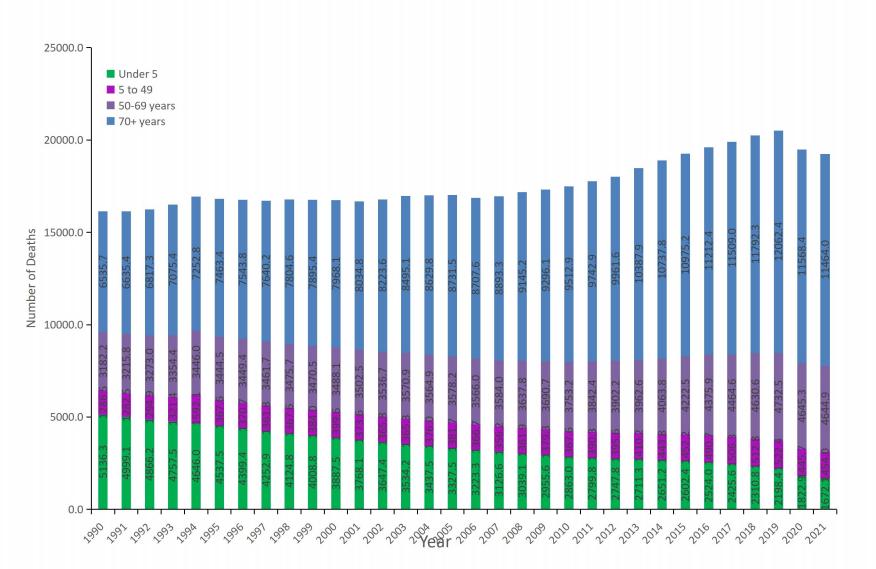


Figure S1:Global time trend of sepsis,by age, 1990-2021


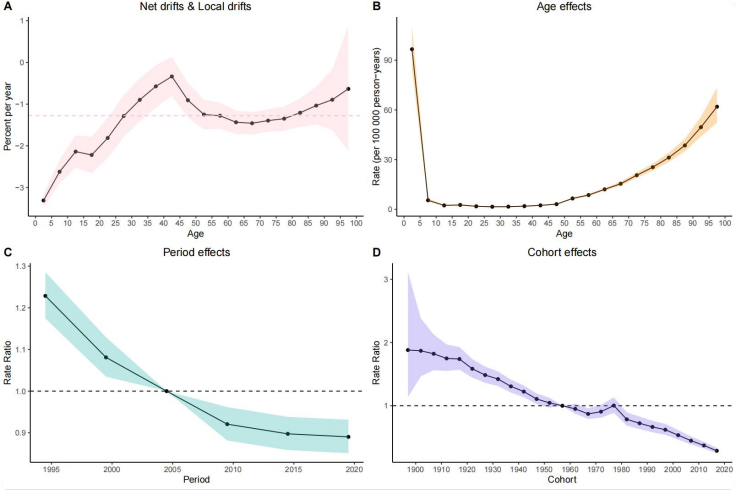


Figure S2: Age, Period, and Cohort Effects on global LRI caused by CMV DALYs : Relative Risk Analysis.(A): Net drifts and Local drifts on on DALYs relative risk.

(B): Age effects on incidence relative risk; (C): Period effects on incidence relative risk; (D): Cohort effects on incidence relative risk;


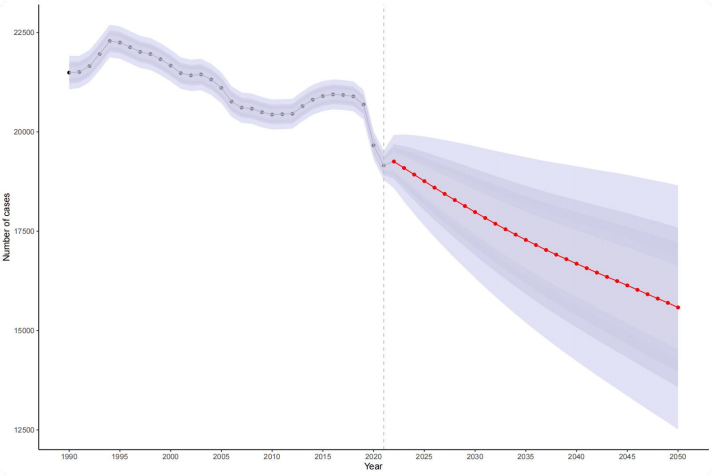


Figure S3:The temporal trend and forecast of LRI caused by CMV globally from 1990 to 2021:Projected age-standardized mortality number;Red dot lines and shaded regions represent the predicted trend and 95% CI.
